# Coral and Coral-Associated Microorganisms: A Prolific Source of Potential Bioactive Natural Products

**DOI:** 10.3390/md17080468

**Published:** 2019-08-11

**Authors:** Vo Thanh Sang, Ton That Huu Dat, Le Ba Vinh, Le Canh Viet Cuong, Phung Thi Thuy Oanh, Hoang Ha, Young Ho Kim, Hoang Le Tuan Anh, Seo Young Yang

**Affiliations:** 1 NTT Hi-Tech Institute, Nguyen Tat Thanh University, 300A Nguyen Tat Thanh, District 5, Ho Chi Minh City 748000, Vietnam; 2 Mientrung Institute for Scientific Research, Vietnam Academy of Science and Technology, 321 Huynh Thuc Khang, Hue City, Thua Thien Hue 531600, Vietnam; 3 College of Pharmacy, Chungnam National University, Daejeon 34134, Korea; 4 Institute of Marine Biochemistry, Vietnam Academy of Science and Technology, 18 Hoang Quoc Viet, Cau Giay, Ha Noi 100000, Vietnam; 5 Institute of Biotechnology, Vietnam Academy of Science and Technology, 18 Hoang Quoc Viet, Cau Giay, Ha Noi 122300, Vietnam; 6 Graduated University of Science and Technology, VAST, 18 Hoang Quoc Viet, Cau Giay, Ha Noi 122300, Vietnam

**Keywords:** bioactive compounds, coral, coral-associated microorganisms, natural products

## Abstract

Marine invertebrates and their associated microorganisms are rich sources of bioactive compounds. Among them, coral and its associated microorganisms are promising providers of marine bioactive compounds. The present review provides an overview of bioactive compounds that are produced by corals and coral-associated microorganisms, covering the literature from 2010 to March 2019. Accordingly, 245 natural products that possess a wide range of potent bioactivities, such as anti-inflammatory, cytotoxic, antimicrobial, antivirus, and antifouling activities, among others, are described in this review.

## 1. Introduction

Marine environments are among the richest and most diverse ecosystems, with an enormous diversity of different life forms. Harsh chemical and physical conditions in the marine environment are important drivers for the production of a wide range of bioactive natural products with structurally unique features [1,2,3]. These marine natural products exhibit a variety of bioactivities that have potential applications in the pharmaceutical and medical fields. 

Marine natural products, especially those from marine invertebrates, are used naturally as a chemical defense to protect organisms from predators, stressful environments, and the encroachment of competitors [4,5,6,7,8]. Due to bioactive and structural diversity, the natural products from marine organisms are considered to be an extraordinary source of new therapeutics that exhibit structural and chemical features not found in the terrestrial environment [9,10,11,12,13]. More than 20,000 novel compounds have been isolated and identified from marine organisms over the past 50 years [14], and more than 300 patents have been approved [15]. In particular, several current drugs and drug candidates in preclinical or clinical trials have been developed from marine natural products [10,12,14,16]. 

Among marine organisms, soft corals are promising providers of marine bioactive compounds and have received considerable attention. Many bioactive natural products with structurally diverse features including terpenoids, diterpenoids, sesquiterpenoids, steroids, and other chemical compounds have been produced by various species of soft corals [17,18,19,20,21]. It has been estimated that the new compounds isolated and identified from soft corals represent more than 20% of the total new marine natural products reported from 2010 to 2011 [17,18]. Importantly, the natural compounds isolated from soft corals exhibit a spectrum of biological activities, such as antimicrobial, anticancer, anti-inflammatory, antiviral, and antifouling activities [17,18,19,21,22].

Corals are also known to harbor diverse and highly abundant microbial communities [23,24,25,26,27,28]. It has been reported that coral’s microbial communities are partially species-specific [25,29,30] and that shifts in the composition of the coral microbiome are related to changes in coral health, the appearance of signs of diseases and/or bleaching, resistance to stressors, and the stability of reef ecosystems [31,32,33,34,35,36]. Past investigations have revealed that the coral microbiome plays an important role in the protection of the coral hosts by producing antimicrobial compounds, inhibiting pathogenic catabolic enzymes, disrupting cell-to-cell communication in pathogens, and competitively excluding pathogens from host surfaces [31,35,37,38,39,40]. Coral-associated bacteria can produce many structurally diverse compounds with a wide range of bioactivities, including antimicrobial compounds, against a broad spectrum of pathogens of corals [38,40]. Thus, not only coral, but also coral-associated microorganisms, are considered to be extraordinary sources of bioactive natural products.

In the past few decades, repeated isolation of known secondary metabolites and a decrease of novel compounds discovered from terrestrial environments have limited the development of new drugs for treating increasing diseases [41]. Particularly, arising drug resistance presents an urgent requirement for the discovery of new bioactive compounds from the marine environment. Hence, the discovery of novel pharmaceutical compounds from the marine environment such as coral and its associated microorganisms is a promising strategy. There have been several reviews on natural products from coral and coral-derived microorganisms over the past few decades. However, these reviews have reported partially on natural products from several specific coral groups/specific structure classes [20,42,43,44,45] or were part of annual reviews about marine natural products [46]. In the present review, we provide a comprehensive overview of bioactive compounds produced by corals and coral-associated microorganisms from 2010 to March 2019, focusing on structure and the bioactivity of potent bioactive compounds with the half maximal inhibitory concentration (IC_50_) (/half maximal effective concentration (EC_50_)/median effective dose (ED_50_)/growth inhibitory dose 50% of cells (GI_50_)) values less than 10 μM (/10 µg/mL).

## 2. Bioactive Compounds from Coral

### 2.1. Anti-Inflammatory Compounds from Coral

Inflammation is a defense response by organisms to hazardous stimuli such as allergens and/or injury to the tissues. In general, inflammation is indispensable in the protection of the body from pathogens and for the repair of damaged tissue. However, an exaggerated and persistent inflammatory response is also detrimental to human health. Uncontrolled inflammatory responses are also involved in the onset and maintenance of many severe disorders such as rheumatoid arthritis, asthma, diabetes, chronic inflammatory bowel diseases, neurodegenerative diseases, and cancer [47]. The high prevalence of inflammation requires the discovery and development of new anti-inflammatory drugs. Currently, the available approved anti-inflammatory drugs mainly consist of nonsteroidal anti-inflammatory drugs, glucocorticoids, and immunosuppressant drugs. However, these therapies are often not effective enough or are hampered by unwanted side effects [48]. Effective anti-inflammatory agents should be able to inhibit the development of inflammation without interfering in normal homeostasis. Thus, the discovery of safe and efficient new anti-inflammatory agents is still a great demand for scientists in academia and industry.

The bioactive investigation of compounds isolated from corals has revealed coral-derived compounds as a potential source of anti-inflammatory drugs. Numerous anti-inflammatory compounds have been isolated from coral with different mechanisms (Supplementary Table S1), of which many compounds exhibit potent anti-inflammatory activities.

**Figure 1:**
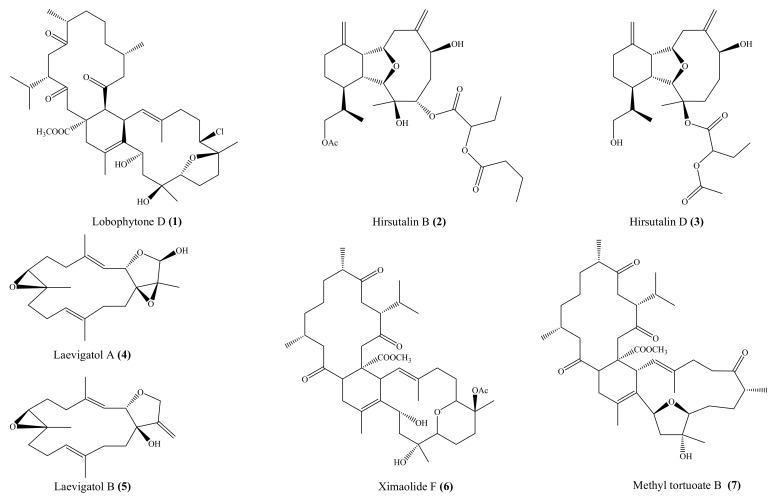
Chemical structures of **1**–**7**.

From the coral *Lobophytum pauciflorum*, seven new biscembranoids, lobophytones A–G, together with three known biscembranes, were isolated. Among them, lobophytone D (**1**) (Figure 1) showed potent inhibition toward lipopolysaccharide (LPS)-induced nitric oxide (NO) in mouse peritoneal macrophages with IC_50_ = 4.70 μM [49]. Chen et al. [50] isolated eight new eunicellin-based diterpenoids, hirsutalins A–H, from the coral *Cladiella hirsuta*. All compounds were evaluated for their anti-inflammatory activity based on inhibiting the upregulation of proinflammatory inducible nitric oxide synthase (iNOS) and cyclooxygenase-2 (COX-2) proteins of LPS-stimulated RAW264.7 macrophage cells. Notably, hirsutalins B (**2**) and D (**3**) (Figure 1) were found to potently reduce the levels of iNOS protein to 6.8% ± 0.6% and 3.3% ± 0.1%, respectively, whereas hirsutalins C and H also effectively reduced the levels of iNOS protein to 43.6% ± 8.7% and 32.3% ± 6.1%, respectively, relative to the control cells stimulated with LPS only. In addition, hirsutalin B also effectively reduced the level of COX-2 protein (49.0% ± 2.3%) [50]. Chemical investigation of the coral *Lobophytum laevigatum* also led to the isolation of four new cembranoids, laevigatols A–D, and six known metabolites. Interestingly, laevigatols A and B (**4**, **5)**, ximaolide F (**6**), and methyl tortuoate B (**7**) (Figure 1) showed dose-dependent inhibitory effects on the tumor necrosis factor α (TNF*α*)-induced (nuclear factor) NF-κB transcriptional activity in Hep-G2 cells, with IC_50_ = 6.7–9.7 µM. Moreover, these compounds effectively inhibited the induction of COX-2 and iNOS mRNA dose-dependently [51].

**Figure 2:**
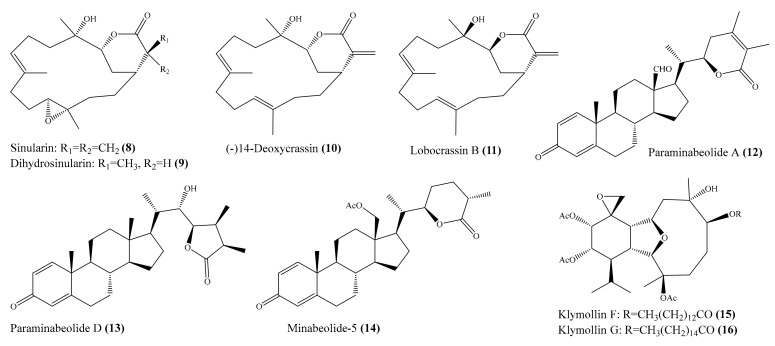
Chemical structures of **8**–**16**.

Two new cembrane-type diterpenoids, triangulenes A and B, along with three known metabolites, were isolated from the coral *Sinularia triangular*. Among them, sinularin (**8**), dihydrosinularin (**9**), and (−)14-deoxycrassin (**10**) (Figure 2) could significantly reduce the levels of the iNOS protein to 1.2% ± 0.3%, 5.1% ± 1.6%, and 0.9% ± 0.7%, respectively, at a concentration of 10 μM. At the same concentration, dihydrosinularin and (-)14-deoxycrassin markedly reduced the levels of COX-2 to 24.9% ± 7.4% and 5.9% ± 1.0%, respectively [52]. In another study, Kao et al. [53] reported the isolation of five new cembrane-type diterpenoids, lobocrassins A–E, from the coral *Lobophytum crissum*. However, only lobocrassin B (**11**) (Figure 2) displayed significant inhibitory effects on the generation of the superoxide anion and the release of elastase by human neutrophils, with IC_50_ = 4.8 ± 0.7 and 4.9 ± 0.4 µg/mL (15.1 ± 2.2 and 15.4 ± 1.3 μM). From the coral *Paraminabea acronocephala*, six new withanolides, paraminabeolides A–F, along with five known compounds, were also isolated. Compound paraminabeolides A–D were found to inhibit the accumulation of the proinflammatory iNOS protein, whereas compound minabeolides-1, -2, -4, and -5 could effectively reduce the expression of both the iNOS and COX-2 protein. Particularly, compound paraminabeolide A (**12**), paraminabeolide D (**13**), and minabeolide-5 (**14**) (Figure 2) potently reduced the levels of iNOS protein to <10% at a concentration of 10 μM [54]. Additionally, Hsu et al. [55] isolated eight new eunicellin-based diterpenoids, klymollins A–H, from the coral *Klyxum molle*. Biological tests indicated that compound klymollins C–H displayed in vitro anti-inflammatory activity by inhibiting the expression of the iNOS protein, whereas compound klymollins A, B, F, G, and H could also reduce the accumulation of COX-2 protein in LPS-stimulated RAW264.7 macrophage cells. Especially, klymollin F (**15**) and klymollin G (**16**) (Figure 2) significantly reduced the levels of the iNOS and COX-2 proteins to <10% at a concentration of 10 μM.

**Figure 3:**
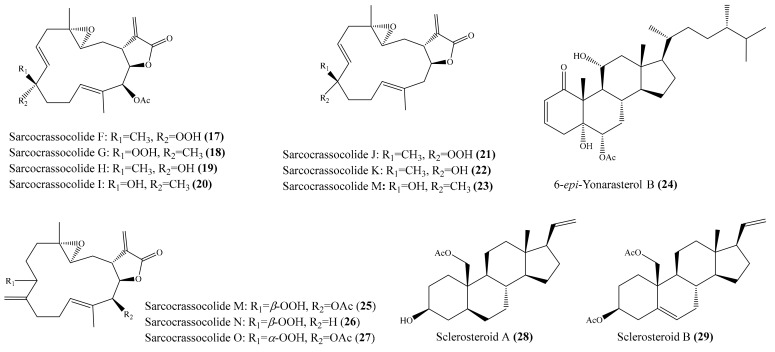
Chemical structures of **17**–**29**.

A chemical study of the coral *Sarcophyton crassocaule* afforded seven new cembranoids, sarcocrassocolides F–L (**17**–**23**) (Figure 3). Interestingly, all of these compounds were found to display potent anti-inflammatory activity by reducing the expression of the iNOS protein to <10% at a concentration of 10 μM. Furthermore, compound **20** was also found to effectively reduce the level of COX-2 protein to <60% [56]. Chung et al. [57] reported the isolation of a new labdane-type diterpenoid, echinolabdane, and a new sterol, 6-*epi*-yonarasterol B, from the coral *Echinomuricea* sp. It is notable that both compounds displayed inhibitory effects on the generation of superoxide anions and the release of elastase by human neutrophils, of which 6-*epi*-yonarasterol B (**24**) (Figure 3) showed potent effects on the generation of superoxide anions and the release of elastase, with IC_50_ = 2.98 ± 0.29 and 1.13 ± 0.55 µg/mL (6.48 ± 0.63 and 2.46 ± 1.20 μM). The activity of **24** was comparable to that of the positive control diphenylene iodonium (DPI), with IC_50_ = 0.82 ± 0.31 µg/mL (2.59 ± 0.98 μM) for the generation of superoxide anions, and elastatinal, with IC_50_ = 31.82 ± 5.92 µg/mL (100.51 ± 18.70 μM) for elastase release. Chemical examination of the coral *Sarcophyton crassocaule* also resulted in the isolation of three new cembranoids, sarcocrassocolides M–O (**25–27**) (Figure 3). Bioassay results revealed that all of these compounds potently inhibited the expression of the iNOS protein and effectively inhibited the expression of the COX-2 protein [58]. In another study, nine new steroids, sclerosteroids A–I, along with 18 known metabolites, were isolated from the coral *Scleronephthya gracillimum*. Among them, compound sclerosteroids A, B, and E displayed in vitro anti-inflammatory activity in LPS-stimulated RAW264.7 macrophage cells by inhibiting the expression of the iNOS protein and COX-2 protein. Especially, at a concentration of 10 μM, sclerosteroids A and B (**28**, **29**) (Figure 3) significantly reduced the levels of COX-2 protein to 5.4% ± 1.3% and 6.7% ± 3.5%, respectively [59].

**Figure 4:**
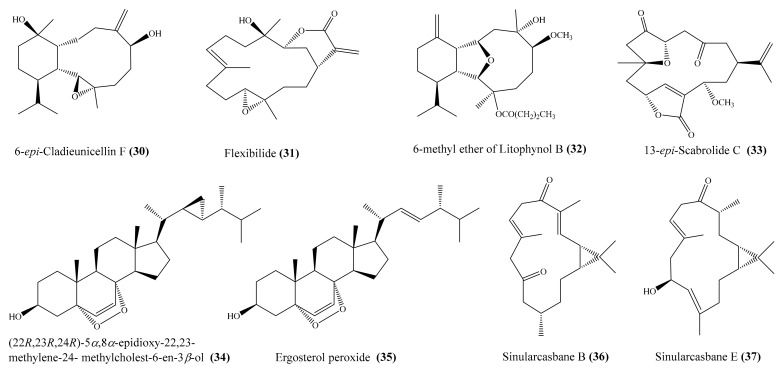
Chemical structures of **30**–**37**.

From the coral *Cladiella* sp., Chen et al. [60] yielded two new 6-hydroxyeunicellin diterpenoids, cladieunicellin G and 6-*epi*-cladieunicellin F, of which 6-*epi*-cladieunicellin F (**30**) (Figure 4) displayed significant inhibitory effects on the generation of the superoxide anion and the release of elastase by human neutrophils, with IC_50_ values of 6.57 ± 0.85 and 41.08 ± 3.26 μg/mL (20.40 ± 2.64 and 127.58 ± 10.12 μM). DPI and elastatinal were used as positive controls, with IC_50_ = 0.82 ± 0.31 µg/mL (2.59 ± 0.98 μM) for the generation of superoxide anions and with IC_50_ = 31.82 ± 5.92 µg/mL (100.51 ± 18.70 μM) for elastase release. Yang et al. [61] reported the isolation of five new cembrane diterpenoids, sinuflexibilins A–E, along with nine other known diterpenoids from the coral *Sinularia* sp. However, bioassays indicated that only the compound flexibilide (**31**) (Figure 4) exhibited significant inhibitory activity of NF-κB activation, with IC_50_ = 5.30 μg/mL (15.87 μM). From the coral *Cladiella krempfi*, five new eunicellin-based diterpenoids, krempfielins E–I, together with seven known compounds, were isolated. At a concentration of 10 μM, three compounds (krempfielin F, 6-methyl ether of litophynol B (**32**) (Figure 4), and 6-acetoxy litophynin E could significantly inhibit the accumulation of the proinflammatory iNOS protein. Among them, 6-methyl ether of litophynol B (**32**) potently reduced the levels of iNOS protein to 6.4% ± 0.8%. In addition, 6-methyl ether of litophynol B (**32**) and litophynin F could also significantly reduce the expression of COX-2 protein in LPS-stimulated RAW264.7 macrophage cells [62]. Thao et al. [63] isolated two new norditerpenoids along with five other known norditerpenoids from the coral *Sinularia maxima*. Among them, compound 13-epi-scabrolide C (**33**) (Figure 4) potently inhibited interleukin (IL)-12 and IL-6 production in LPS-stimulated bone marrow-derived dendritic cells (BMDCs), with IC_50_ values of 5.30 ± 0.21 and 13.12 ± 0.64 µM, respectively, whereas compound scabrolide A also exhibited moderate inhibitory activity against IL-12 and IL-6 production, with IC_50_ values of 23.52 ± 1.37 and 69.85 ± 4.11 µM, respectively. The positive control SB203580, an inhibitor of cytokine-suppressive binding protein/p38 kinase, inhibited IL-12 and IL-6, with IC_50_ of 5.67 ± 0.24 and 3.56 ± 0.11 µM, respectively. Chemical investigation of the coral *Lobophytum crassum* led to the isolation of one new compound, crassumsterol, along with three known compounds. Interestingly, (22*R*,23*R*,24*R*)-5*α*,8*α*-epidioxy-22,23-methylene-24-methylcholest-6-en-3β-ol (**34**) and ergosterol peroxide (**35**) (Figure 4) had potent inhibitory effects on TNFα-induced NF-κB transcriptional activation in Hep-G2 cells, with IC_50_ values of 3.90 and 7.05 µM, respectively [64]. Yin et al. [65] afforded six new casbane diterpenoids, sinularcasbanes A–F, along with six known analogues, from the coral *Sinularia* sp. Among them, compound sinularcasbanes B and E (**36**, **37**) (Figure 4) showed potent inhibition of lipopolysaccharide LPS-induced NO production in mouse peritoneal macrophages, with IC_50_ values of 8.3 and 5.4 μM, respectively. 

**Figure 5:**
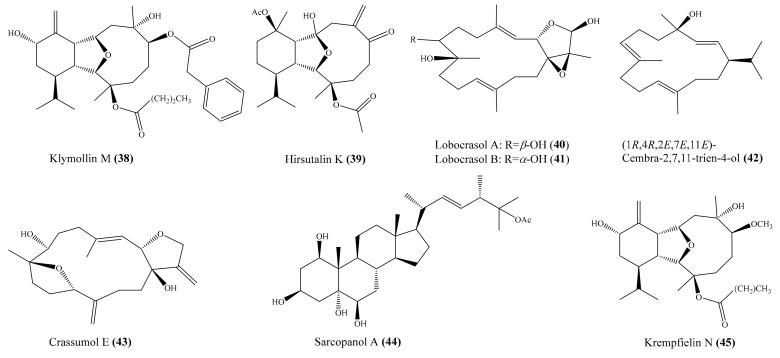
Chemical structures of **38**–**45**.

Chemical examination of the coral *Klyxum molle* yielded 11 new eunicellin-based diterpenoids, klymollins I–S. Among them, compound klymollin M (**38**) (Figure 5) was the first eunicellin-based metabolite bearing a phenyl group and displayed significant inhibition of both superoxide anion generation and elastase release in *N-*formyl-L-methionyl-L-leucyl-Lphenylalanine/cytochalasin B (fMLP/ CB)-induced human neutrophils, with IC_50_ values of 3.13 ± 0.39 μM and 2.92 ± 0.27 μM, respectively [66]. From the soft coral *Cladiella hirsuta*, Chen et al. [67] obtained five new eunicellin-based diterpenoids, hirsutalins I–M. Interestingly, compound hirsutalin K (**39**) (Figure 5) was found to possess the strongest NO inhibitory activity, with an IC_50_ value of 9.8 µg/mL (22.5 μM), while the positive control curcumin exhibited NO inhibitory activity, with an IC_50_ value of 10 µg/mL (27.1 μM). Furthermore, **39** effectively reduced the expression of iNOS protein in the same cells. From another coral, *Lobophytum crassum*, four new cembranoid diterpenes, lobocrasols A–D, were isolated. Bioassays showed that lobocrasols A and B (**40**, **41**) (Figure 5) potently inhibited TNF*α*-induced NF-*κ*B transcriptional activity in HepG2 cells in a dose-dependent manner, with IC_50_ values of 6.30 ± 0.42 and 6.63 ± 0.11 μM, respectively. Besides, the transcriptional inhibition of these compounds was confirmed by a decrease in COX-2 and iNOS expression levels in HepG2 cells [68]. From the same coral species, Cuong et al. [69] isolated four new cembranoid diterpenes, crassumols D–G, along with five known compounds. Interestingly, compounds (1*R*,4*R*,2*E*,7*E*,11*E*)-cembra-2,7,11-trien-4-ol (**42**) and crassumol E (**43**) (Figure 5) exhibited a potent inhibitory effect on TNF*α*-induced NF-*κ*B transcriptional activation in HepG2 cells, with IC_50_ values of 1.65 ± 0.2 and 9.23 ± 1.66 µM, respectively. Furthermore, compound **42** effectively inhibited the expression of COX-2 and iNOS protein in a dose-dependent manner, indicating that this compound reduced the transcription of these genes. From the coral *Sarcophyton pauciplicatum*, a new steroid, sarcopanol A, along with two known ones, were isolated. Interestingly, compound sarcopanol A (**44**) (Figure 5) exhibited significant inhibitory effects on TNF*α*-induced NF-*κ*B transcriptional activity, with an EC_50_ value of 8.27 ± 3.28 µM, whereas compounds (24*S*)-ergost-1b,3b,5a,6b-tetraol-25-monoacetate and (24*S*)-ergost-25-ene-1β,3β,5*α*,6β-tetraol exhibited moderate effects, with EC_50_ values of 26.07 ± 5.59 and 50 µM, respectively. The positive control apigenin exhibited activity, with EC_50_ = 4.51 ± 0.53 µM. Furthermore, the transcriptional inhibition of these compounds was confirmed by a decrease in cyclooxygenase-2 (COX-2), inducible nitric oxide synthase (iNOS), and intercellular adhesion molecule-1 (ICAM-1) gene expression levels in HaCaT cells [70]. From the coral *Cladiella krempfi,* Lee et al. [71] afforded three new eunicellin-type diterpenoids, krempfielins N–P. Compound krempfielin N (**45**) (Figure 5) exhibited potent activity in inhibiting elastase release in fMLP/CB-induced human neutrophils, with IC_50_ of 4.94 ± 1.68 µM, where the remaining two compounds showed weak activity in inhibiting elastase release, with IC_50_ > 10 µM. The activity of **45** was comparable to that of the positive control LY294002 (a phosphatidylinositol-3-kinase inhibitor), with IC_50_ values of 4.12 ± 0.92 µM. Additionally, compound krempfielin P inhibited 23.32% ± 5.88% generation of the superoxide anion. 

**Figure 6:**
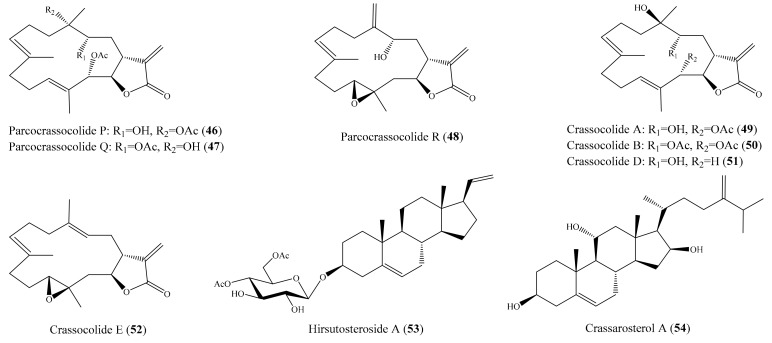
Chemical structure of **46**–**54**.

Chemical investigation of the coral *Sarcophyton crassocaule* afforded three new cembranoids, sarcocrassocolides P–R (**46**–**48**), and four known compound crassocolides: A, B, D, and E (**49**–**52**) (Figure 6). All of these compounds displayed potent in vitro anti-inflammatory activity in lipopolysaccharide (LPS)-stimulated RAW264.7 macrophage cells by inhibiting the expression of iNOS protein to 3.5% at a concentration of 10 µM, whereas compounds **47**, **49**, and **52** also showed moderate activity in reducing the accumulation of COX-2 protein at the same concentration [72]. From the coral *Cladiella hirsuta*, two new pregnane glycosides, hirsutosterosides A and B, along with two new atocopherylhydroquinone glycosides, cladophenol glycosides A and B, were isolated. In addition, a new steroidal glycoside, lobatasteroside A, along with six known steroids, were isolated from the coral *Sinularia nanolobata*. Compound hirsutosteroside A (**53**) (Figure 6) did not exhibit inhibition activity toward superoxide anion generation (IC_50_ > 10 µM) but potently inhibited fMLP/CB-induced elastase release, with IC_50_ values of 4.1 ± 0.1 µM. Compound 5β,6β-epoxy-3β,11-dihydroxy-24-methylene-9,11-secocholestan-9-one showed moderate activities toward superoxide anion generation and elastase release, with IC_50_ values of 18.6 ± 1.5 and 10.1 ± 0.8 µM, respectively, while compound crassarosterol A (**54**) (Figure 6) significantly inhibited superoxide anion generation and elastase release, with IC_50_ values of 6.6 ± 0.6 and 2.9 ± 0.5 µM, respectively [73]. LY294002 was used as a positive control for the inhibition of superoxide anion generation and elastase release, with IC_50_ values of 0.6 ± 0.1 and 1.2 ± 0.3 µg/mL (1.95 ± 0.33 and 3.90 ± 0.98 µM), respectively.

**Figure 7:**
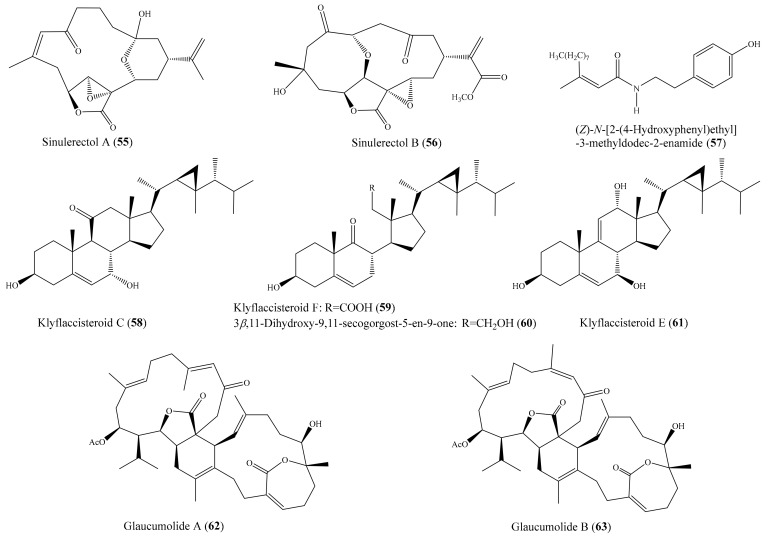
Chemical structures of **55**–**63**.

Chemical investigation of the coral *Sinularia erecta* yielded four new isoprenoids and four known compounds. Biological tests indicated that three compounds, sinulerectols A and B (**55**, **56**) and (*Z*)-*N*-[2-(4 hydroxyphenyl)ethyl]-3-methyldodec-2-enamide (**57**) (Figure 7), displayed potent anti-inflammatory activity in fMLP/CB-stimulated human neutrophils, with IC_50_ values from 0.9 ± 0.1 to 8.5 ± 0.3 µM [74]. Tsai et al. [75] reported the isolation of six new steroids, klyflaccisteroids A–E; a new 9,11-secogorgosteroid, klyflaccisteroid F; two known steroids; and a known eunicellin-based diterpenoid from the coral *Klyxum flaccidum*. It is notable that compounds klyflaccisteroid C (**58**), klyflaccisteroid F (**59**), and 3β,11-dihydroxy-9,11-secogorgost-5-en-9-one (**60**) (Figure 7) exhibited potent anti-inflammatory activity in inhibiting both superoxide generation and elastase release in fMLP/CB-induced neutrophils, with IC_50_ values from 0.34 ± 0.01 to 4.78 ± 0.87 µM, whereas klyflaccisteroid E (**61**) (Figure 7) only exhibited potent activity in inhibiting elastase release, with an IC_50_ value of 5.37 ± 0.20 µM. From another coral, *Sarcophyton glaucum,* two novel biscembranes, glaucumolides A and B (**62**, **63**) (Figure 7), were obtained. Both compounds displayed strong inhibition of superoxide anion generation and elastase release in fMLP/CB-induced human neutrophils, with IC_50_ values from 2.79 ± 0.32 to 3.97 ± 0.10 µM. Furthermore, these compounds significantly inhibited the accumulation of the proinflammatory inducible nitric oxide synthase protein and the expression of COX-2 protein in lipopolysaccharide-stimulated RAW264.7 macrophage cells [76].

**Figure 8:**
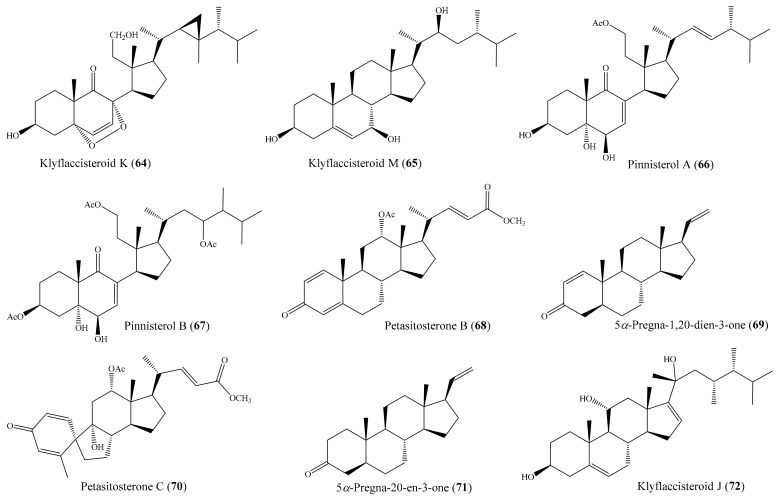
Chemical structures of **64**–**72**.

From the coral *Klyxum flaccidum,* three new steroids, klyflaccisteroids K–M, were isolated. Compound klyflaccisteroid K (**64**) (Figure 8) was found to display significant anti-inflammatory activity in suppressing superoxide anion generation and elastase release, with IC_50_ values of 5.83 ± 0.62 and 1.55 ± 0.21 µM, respectively, whereas compound klyflaccisteroid M (**65**) (Figure 8) was also found to show notable anti-inflammatory activity toward elastase release, with an IC_50_ value of 5.84 ± 0.33 µM [77]. A chemical study of the coral *Pinnigorgia* sp. resulted in the isolation of three new 9,11-secosterols, pinnisterols A–C, of which two compounds (pinnisterols A and C (**66** and **67**) (Figure 8) displayed remarkable inhibitory effects on the generation of superoxide anions and the release of elastase in human neutrophils, with IC_50_ values from 2.33 to 3.32 µM [78]. From the coral *Umbellulifera petasites*, Huang et al. [79] reported the isolation of three new steroids, petasitosterones A–C, along with eight known steroids. The compound petasitosterone B (**68**) (Figure 8) effectively reduced NO production to 16.9% at 10 µg/mL (22.7 µM), whereas 5*α*-pregna-1,20-dien-3-one (**69**) (Figure 8) showed potent inhibition of NO production to 0.3% at the same concentration (10 µg/mL (33.6 µM)). Interestingly, both of these compounds exhibited better inhibitory activity than the positive control aminoguanidine at the same concentration (10 µg/mL (135.0 µM)). Furthermore, compound **68** and petasitosterone C (**70**) (Figure 8) showed potent inhibition toward superoxide anion generation, with IC_50_ values of 4.43 ± 0.23 and 2.76 ± 0.92 µM, respectively, whereas compound 5*α*-pregna-20-en-3-one (**71**) (Figure 8) significantly inhibited fMLP/CB-induced elastase release, with an IC_50_ value of 6.80 ± 0.18 µM. A chemical investigation of the coral *Klyxum flaccidum* afforded four new steroids, klyflaccisteroids G–J. Interestingly, compound klyflaccisteroid J (**72**) (Figure 8) was found to display significant anti-inflammatory activity in suppressing superoxide anion generation and elastase release, with IC_50_ values of 5.64 ± 0.41 and 4.40 ± 0.19 µM, respectively, whereas compound klyflaccisteroids G and H showed weak inhibition of superoxide anion generation and elastase release, with IC_50_ > 10 µM. Notably, the anti-inflammatory activity of compound **72** toward suppressing superoxide anion generation and elastase release was comparable to that of the positive control LY294002 (IC_50_ = 3.65 ± 1.14 and 5.15 ± 1.17 µM for superoxide anion and elastase, respectively) [80]. 

**Figure 9:**
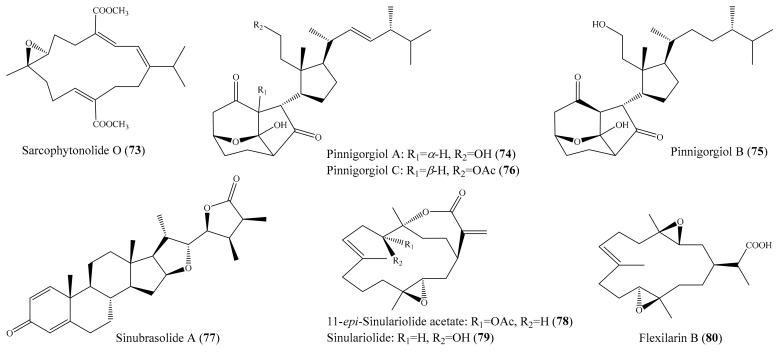
Chemical structures of **66**–**80**.

Ten new cembrane-based diterpenes, locrassumins A–G, (−)-laevigatol B, (−)-isosarcophine, and (−)-7*R*,8*S*-dihydroxydeepoxysarcophytoxide, together with eight known analogues, were isolated from the coral *Lobophytum crassum*. Compound sarcophytonolide O (**73**) (Figure 9) exhibited significant inhibition against LPS-induced NO production, with an IC_50_ value of 8 µM, whereas compounds locrassumin A, locrassumin G, *ent*-sarcophine, and ketoemblide also displayed effective inhibition against LPS-induced NO production, with IC_50_ values of 12–24 µM [81]. In another investigation, Chang et al. [82] yielded three novel 9,11-secosterols with a rare carbon skeleton arrangement, pinnigorgiols A–C (**74**–**76**) (Figure 9), from the coral *Pinnigorgia* sp. Interestingly, all three compounds displayed remarkable inhibition effects on the generation of superoxide anions and the release of elastase by human neutrophils, with IC_50_ values from 2.7–5.3 µM. Chemical investigation of the coral *Sinularia brassica* obtained five new withanolides, sinubrasolides H–L, together with the known compound sinubrasolide A. Although all six isolated compounds displayed inhibitory effects against superoxide anion generation and elastase release in fMLP/CB-stimulated cells, only compound sinubrasolide A (**77**) (Figure 9) showed potent inhibitory effects against superoxide anion generation and elastase release, with IC_50_ values of 3.5 ± 0.9 and 1.4 ± 0.1 µM, respectively [83]. From the coral *Sinularia flexibilis,* three new flexibilide-like cembranoids, xidaosinularides A–C, along with six known flexibilides, were obtained. Among them, compounds 11-*epi*-sinulariolide acetate (**78**), sinulariolide (**79**), and flexilarin B (**80**) (Figure 9) exhibited strong anti-inflammatory effects through the accumulation of proinflammatory TNF-α, with IC_50_ values of 2.7, 4.7, and 4.2 μM, respectively. However, it is of interest that compound 5-dehydrosinulariolide, with a carbonyl group at the C-5 position, displayed no activity (IC_50_ > 50 μM), whereas **78** and **79**, whose structures only differed from that of 5-dehydrosinulariolide in the C-5 position, exhibited significant anti-inflammatory activity, suggesting that the carbonyl at the C-5 position could reduce the inhibition of the accumulation of proinflammatory TNF-α [84].

### 2.2. Cytotoxic Compounds from Coral

Cancers are known as “a group of diseases characterized by the uncontrolled growth and spread of abnormal cell” and are a major cause of death worldwide. Currently, the main treatments for cancer involve the development of surgical procedures, the use of radiotherapy and/or chemotherapeutic agents, as well as a combination of chemotherapy and hormone therapy with immunotherapy [85,86]. Despite their severe toxicity, chemotherapy, radiotherapy, and immunotherapy are the gold standard approaches to the treatment of cancer. As they are less toxic, the discovery and use of natural products is being tried as an alternative in the treatment of cancer [87]. It is noted that several factors should be accounted for in the development of effective therapies for cancer. One of the main problems that needs to be of concern in cancer treatment is to minimize the side effects of the therapies [88]. Another problem that needs to be considered is the development of resistant phenotypes, which include cytotoxic resistance to proapoptotic stimuli and/or anticancer agents. Despite all attempts to prevent oncological disorders and develop new therapies, cancer remains a serious public health problem. Thus, there is increasing attention on strategies to effectively control tumors, prolong survival, minimize chemotherapy side effects, and improve the quality of life for patients [89]. From this point of view, a demand for the discovery of safe and effective new cytotoxic agents for the treatment of cancer is needed.

Apart from anti-inflammatory activity, many cytotoxic compounds have been produced by coral (Supplementary Table S2), of which many compounds show potent cytotoxicity against different cancer cell lines, with IC_50_ values less than 10 μM or 10 µg/mL. 

**Figure 10:**
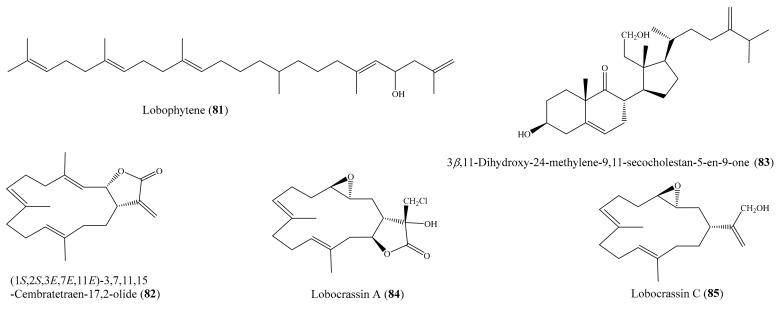
Chemical structures of **81**–**85**.

Chemical study of the coral *Lobophytum* sp. resulted in the isolation of one new squalene derivative, lobophytene, along with three cembranoid diterpenes and two sterols. The compounds lobophytene (**81**) and (1*S*,2*S*,3*E*,7*E*,11*E*)-3,7,11,15-cembratetraen-17,2-olide (**82**) (Figure 10) showed significant cytotoxic activities against lung (A549) and colon (HT-29) cell lines, with IC_50_ values from 1.8 to 8.2 µM. The cytotoxic activities of **81** and **82** were comparable to those of the positive control mitoxantrone. Mitoxantrone showed cytotoxicity against the A549 and HT-29 cell lines, with IC_50_ values of 6.1 and 6.5 µM, respectively [90]. From *Lobophytum compactum*, two new diterpenes, lobocompactols A and B, along with five known compounds, were isolated. Subsequent bioassays indicated that compound 3β,11-dihydroxy-24-methylene-9,11-secocholestan-5-en-9-one (**83**) (Figure 10) exhibited strong cytotoxic activity against the A549 cell line, with an IC_50_ of 4.97 ± 0.06 µM, whereas compounds lobatrienolide, lobatriene, and (24*S*)-ergostane-3β,5*α*,6β,25-tetraol 25-monoacetate showed moderate activity against the A549 cell line. Furthermore, all of these compounds exhibited moderate cytotoxicity against the HL-60 cell line. Interestingly, the cytotoxic activity of **83** against the A549 cells was comparable to that of the positive control mitoxantrone (IC_50_ = 7.83 ± 0.04 µM) [91]. Kao et al. [53] reported the isolation of five new cembrane-type diterpenoids from the coral *Lobophytum crissum* (see Section 2.1). Interestingly, four compounds, lobocrassins A–D exhibited cytotoxicity toward different tumor cell lines (K562, CCRF-CEM, Molt4, HepG2, Huh7). Notably, compound lobocrassin A (**84**) (Figure 10) displayed strong cytotoxicity toward the CCRF-CEM cell line, with an IC_50_ value of 5.33 µg/mL (15.1 µM); compound lobocrassin B (**11**) (Figure 2) displayed strong cytotoxicity toward all of the above cell lines, with IC_50_ values from 0.34 to 8.17 µg/mL (1.07 to 25.7 µM); whereas lobocrassin C (**85**) (Figure 10) displayed strong cytotoxicity toward the MOLT-4 cell line, with an IC_50_ value of 9.51 µg/mL (32.8 µM). Doxorubicin was used as a reference compound, with IC_50_ values ranging from 0.07 to 0.71 µg/mL (0.13 to 1.31 µM).

**Figure 11:**
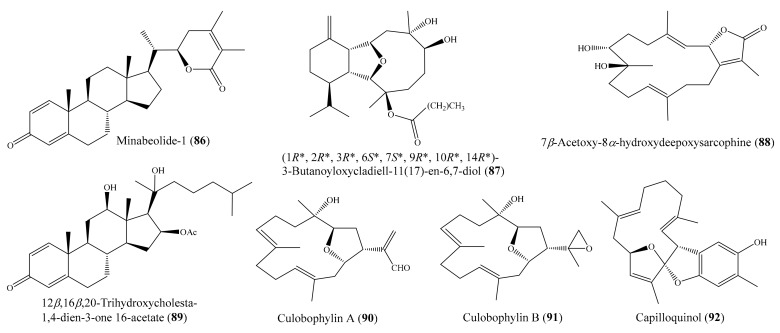
Chemical structures of **86**–**92**.

In another study, six new withanolides, along with five known compounds, were obtained from the coral *Paraminabea acronocephala* (see Section 2.1). Notably, the compounds paraminabeolide A (**12**) (Figure 2) and minabeolide-1 (**86**) (Figure 11) were found to exhibit remarkable cytotoxic activity toward HepG2 cancer cells, with IC_50_ values of 8.0 and 5.2 µM, respectively [54]. From the coral *Cladiella krempfi*, four new eunicellin-based diterpenoids, krempfielins A–D, along with two known compounds, were isolated. Although two compounds, litophynol B and (1R*, 2R*, 3R*, 6S*, 7S*, 9R*, 10R*, 14R*)-3-butanoyloxycladiell-11(17)-en-6,7-diol (**87**) (Figure 11), exhibited cytotoxicity against a limited panel of cancer cell lines (e.g., A549, BT483, MCF-7, SAS, H1299), only compound **87** showed potent cytotoxicity toward BT483 cell lines, with IC_50_ values of 8.5 ± 1.0 μg/mL (21.6 ± 2.5 µM). It is worthwhile to mention that two of these compounds were found to be noncytotoxic toward the normal cell line BEAS2B [92]. From the coral *Sarcophyton auritum*, Hegazy et al. [93] afforded one new cembrane diterpene and three known compounds. However, only compound 7β-acetoxy-8*α*-hydroxydeepoxysarcophine (**88**) (Figure 11) was found to display high cytotoxicity against HepG2, HTC-116, and HeLa cell lines, with IC_50_ values from 2.3 ± 1.5 to 6.7 ± 0.8 µg/mL (6.9 ± 4.5 to 20.1 ± 2.4 µM). Chemical investigation of the coral *Sinularia* sp. yielded the isolation of two new polyhydroxysteroids, 12β,16β,20-trihydroxycholesta-1,4-dien-3-one 16-acetate (**89**) (Figure 11) and 24-methyl-12β,16β,20-trihydroxycholesta-1,4-dien-3-one. Two of these compounds showed cytotoxic effects against the MCF-7, Bel-7402, and HeLa cell lines. However, only compound **89** exhibited potent cytotoxicity toward MCF-7 cells, with an IC_50_ value of 3.82 μg/mL (8.1 µM) [94]. From the coral *Lobophytum crassum,* three new cembranoids, culobophylins A–C, along with two known compounds, were isolated. Interestingly, compound culobophylin A (**90**) (Figure 11) exhibited significant cytotoxic activity against MDA-MB-231, DLD-1, and HCT-116 cancer cell lines, with IC_50_ values of 3.0, 16.8, 4.6, and 16.3 μg/mL (9.4, 52.8, 14.5, and 51.3 µM), respectively. Compound culobophylin B (**91**) (Figure 11) also exhibited cytotoxicity against HL60, DLD-1, and HCT-116 cell lines, with IC_50_ values of 6.8, 16.2, and 16.7 μg/mL (21.3, 50.6, and 52.2 µM), respectively. The cytotoxic activity of **90** against DLD-1 (colon) cells was comparable to that of the positive control doxorubicin C (IC_50_ = 5.7 μg/mL or 10.5 µM) [95]. Chemical investigation of the coral *Sinularia capillosa* led to the isolation of capilloquinol (**92**) (Figure 11), which possessed an unprecedented farnesyl quinoid skeleton. Compound **92** displayed cytotoxicity against the P-388 cell line, with an median effective dose (ED_50_) value of 3.8 μg/mL (10.8 µM) [96].

**Figure 12:**
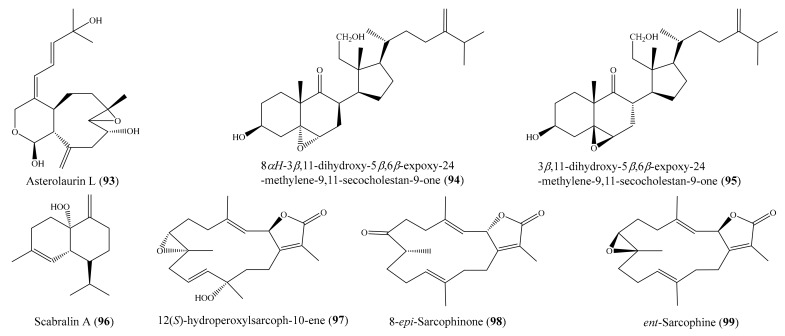
Chemical structures of **93**–**99**.

In another study, four new cembranoids and six known metabolites were isolated from the coral *Lobophytum laevigatum* (see Section 2.1). Compounds (+)-sarcophine, emblide, ximaolide F (**6**), methyl tortuoate B (**7**) (Figure 1), and nyalolide exhibited cytotoxic activity against selected human cancer cell lines (HL-60, A549, HCT-116, and MCF-7), with IC_50_ values ranging from 9.0 to 38.8 µM [51]. Notably, the cytotoxic activity of **6** against the cell line HL-60 was comparable to that of the positive control mitoxantrone (IC_50_ = 7.9 ± 0.3 µM). Lin et al. [56] isolated seven new cembranoids, sarcocrassocolides F–L, from the soft coral *Sarcophyton crassocaule*. All of these compounds exhibited significant cytotoxic activity against at least one cancer cell line (Daoy, HEp-2, MCF-7, and WiDr). Notably, compound sarcocrassocolide I (**20**) (Figure 3) displayed significant cytotoxicity against all of the above cell lines, with ED_50_ values of 5.1 ± 1.2, 5.8 ± 0.5, 8.4 ± 1.5, and 6.4 ± 2.0 μM, respectively, suggesting that the acetoxy group of C-13 is important for the cytotoxicity of compound sarcocrassocolides F–L. It was also found that the hydroxyl group at C-8 could enhance the cytotoxicity of cembranoid sarcocrassocolides F–L in comparison to C-8 hydroperoxy-bearing analogues [56]. In another chemical investigation, Lin et al. [97] obtained three new xenicane diterpenoids, asterolaurins K–M, from the coral *Asterospicularia laurae*. However, only compound asterolaurin L (**93**) (Figure 12) exhibited cytotoxic activity against HEp-2, Daoy, MCF-7, and WiDr tumor cells, with IC_50_ values of 4.12, 6.23, 40.9, and 6.08 μg/mL (11.8, 17.8, 116.9, and 17.4 μM), respectively. From *Sinularia granosa*, a new 9,11-secosteroid, 8*α*H-3β,11-dihydroxy-5*α*,6*α*-expoxy-24-methylene-9,11-secocholestan-9-one (**94**), along with one known steroid, 3β,11-dihydroxy-5β,6β-expoxy-24-methylene-9,11-secocholestan-9-one (**95**) (Figure 12), were isolated. Interestingly, compound **94** exhibited strong cytotoxicity against HeLa, HEp 2, Daoy, and MCF-7 cancer cell lines, with ED_50_ values of 8.21 ± 1.61, 6.21 ± 1.38, 5.53 ± 1.58, and 4.99 ± 0.70 µg/mL (18.4 ± 3.61, 13.9 ± 3.09, 12.4 ± 3.54, and 11.2 ± 1.57 μM), respectively, whereas compound **95** was found to be cytotoxic against Daoy and MCF-7 cancer cell lines, with ED_50_ values of 7.07 ± 0.71 and 9.98 ± 0.32 µg/mL (15.9 ± 1.59 and 22.4 ± 0.72 μM), respectively [98]. From the coral *Sinularia scabra*, two new cadinane-type sesquiterpenoids, scabralins A and B, were obtained. However, only compound scabralin A (**96**) (Figure 12) exhibited cytotoxicity against MCF-7, WiDr, Daoy, and HEp 2 cancer cell lines, with ED_50_ values ranging from 7.6 to 13.8 μg/mL (or 32.2 to 58.5 μM) [99]. Two new hydroperoxyl cembranolides, a cembrene derivative (8-*epi*-sarcophinone), and two known cembranolides were isolated from the coral *Sarcophyton glaucum*. Among them, compounds 12(*S*)-hydroperoxylsarcoph-10-ene (**97**), 8-*epi*-sarcophinone (**98**), and *ent*-sarcophine (**99**) (Figure 12) were found to be potent inhibitors of cytochrome P450 1A activity, with IC_50_ values of 2.7, 3.7, and 3.4 nM, respectively. Furthermore, compounds **97** and **98** were promising inducers of glutathione *S*-transferase (GST) and quinone reductase (QR) activity in in vitro assays [100]. 

**Figure 13:**
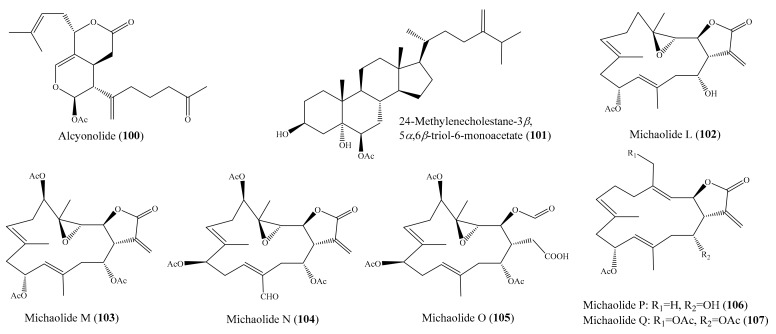
Chemical structures of **100**–**107**.

From the coral *Cespitularia* sp., five new diterpenoids, together with the known diterpenoid alcyonolide (**100**) (Figure 13), were also isolated. Interestingly, **100** exhibited strong cytotoxicity against HCT-116 cells, with an IC_50_ value of 5.85 μM, while new diterpenoids were much less active, with IC_50_ values ranging from 28.2 to 91.4 μM. It is likely that the lactone moiety (C-6–C-5–C-4a–C-4–C-12) and/or the acetal at C-1 are necessary for cytotoxicity [101]. In another study, three new polyoxygenated sterols, together with three known sterols, were isolated from the coral *Sinularia* sp. Among them, compound 24-methylenecholestane-3β,5*α*,6β-triol-6-monoacetate (**101**) (Figure 13) exhibited cytotoxicity against the K562 cell line, with an IC_50_ value of 3.18 μM, but it also displayed strong lethality toward the brine shrimp *Artemia salina*, with an LC_50_ value of 0.96 μM [102]. Chemical investigation of the coral *Lobophytum michaelae* resulted in the isolation of six new cembranolides, michaolides L–Q (**102**–**107**) (Figure 1). Interestingly, all compounds, except for **105,** exhibited remarkable cytotoxicity against P-388, HT-29, A-549, and HEL cell lines, with IC_50_ values ranging from 0.4 to 4.9 μM. It was suggested that the α-exo-methylene-γ-lactone moiety was important for cytotoxicity after a comparison of the cytotoxicity of **105** to **102**–**104**, **106**, and **107** [103].

**Figure 14:**
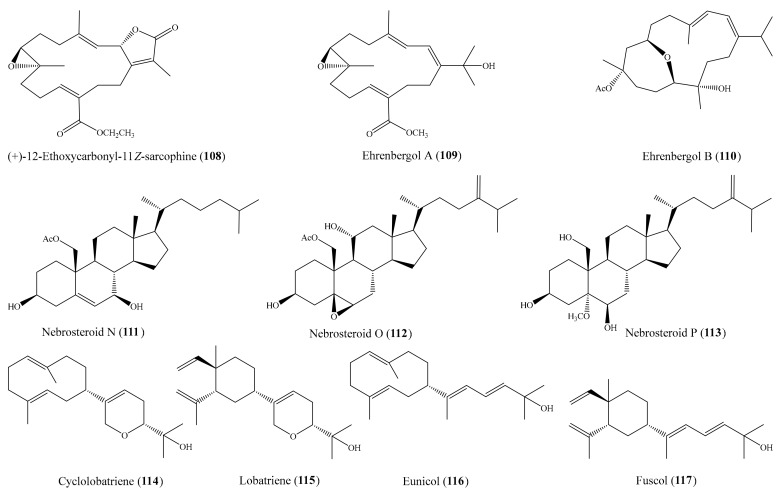
Chemical structures of **108**–**117**.

From the coral *Sarcophyton ehrenbergi,* three new cembranoids ((+)-12-ethoxycarbonyl-11*Z*-sarcophine (**108**) and ehrenbergols A and B (**109** and **110**)) (Figure 14) were isolated. All of these new compounds displayed strong cytotoxicity against the P-388 cell line, with ED_50_ values of 5.8, 7.4, and 4.7 μg/mL (15.5, 21.3, 12.4 µM), respectively, whereas **108** and **110** showed moderate activity toward the A549 cell line, with ED_50_ values of 20.8 and 10.2 μg/mL (55.6 and 27.0 µM), respectively [104]. In another study, three new 19-oxygenated steroids, nebrosteroids N–P (**111**–**113**) (Figure 14), were also isolated from the coral *Nephthea chabrolii.* Interestingly, three new steroids exhibited potent cytotoxicity against the P-388 cell line, with ED_50_ values of 0.9, 1.2, and 1.7 μg/mL, respectively (1.96, 2.46, and 3.68 µM). The positive control mithramycin showed cytotoxicity against the A549, HT-29, and P-388 cancer cell lines, with IC_50_ values of 0.18, 0.21, and 0.15 μg/mL (0.17, 0.19, and 0.14 µM), respectively, but no cytotoxicity against HeLa cells [105]. Chemical investigation of the coral *Lobophytum pauciflorum* afforded a new 10-membered-ring diterpene, cyclolobatriene (**114**), along with three other known diterpenes: lobatriene (**115**), eunicol (**116**), and fuscol (**117**) (Figure 14). All four compounds showed potent cytotoxic effects against human epidermoid carcinoma A431 cells, with IC_50_ values of 0.64, 0.41, 0.35, and 0.52 µM, respectively [106]. From the coral *Sarcophyton crassocaule*, three new cembranoids, sarcocrassocolides M–O (**25**–**27**), were isolated (see Section 2.1). All of the new cembranoids were found to exhibit cytotoxicity toward the Daoy, HEp-2, MCF-7, and WiDr cancer cell lines. Notably, all three compounds displayed strong cytotoxicity against the Daoy cell line, with ED_50_ values of 6.6 ± 0.8, 5.2 ± 0.6, and 5.0 ± 0.7 µM, respectively, whereas compound **27** showed remarkable cytotoxicity against the MCF-7 cell line, with an ED_50_ value of 6.4 ± 0.5 µM [58].

**Figure 15:**
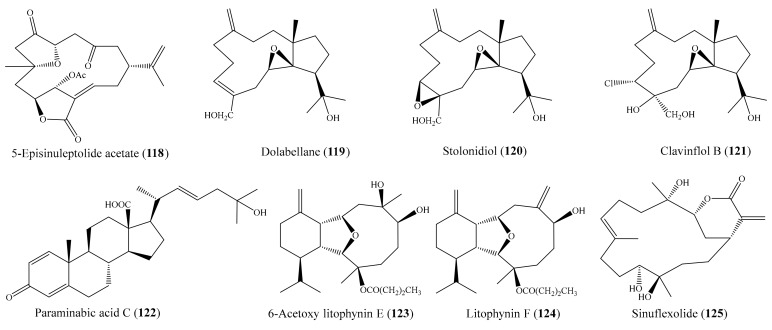
Chemical structures of **118**–**125**.

Chemical investigation of the coral *Sinularia* sp. led to the isolation of two norcembranoidal diterpenes, 5-episinuleptolide acetate (**118**) (Figure 15) and scabrolide D. However, only compound **118** was found to exhibit cytotoxicity toward K562, MOLT-4, HTC-11, DLD-1, T-47D, and MDA-MB-231 tumor cells, with IC_50_ values ranging from 0.59 to 4.09 µg/mL (1.51 to 10.49 µM). The positive control doxorubicin showed cytotoxicity against the K562, MOLT-4, HTC-11, DLD-1, T-47D, and MDA-MB-231 cancer cell lines, with IC_50_ values of 0.15, 0.01, 1.11, 0.22, 0.40, and 1.30 μg/mL, respectively (0.28, 0.02, 2.04, 0.40, 0.74, 2.39 µM) [107]. In another chemical investigation, one new dolabellane (**119**) and two known diterpenoids (stolonidiol (**120**) and clavinflol B (**121**)) (Figure 15) were isolated from the coral *Anthelia* sp. Among them, compound **119** exhibited cytotoxicity against NBT-T2 cells at 10 μg/mL (31.25 µM), while known compounds **120** and **121** showed stronger cytotoxicity at 1.0 and 0.5 μg/mL (2.98 and 1.34 µM), respectively [108]. Chao et al. [109] isolated three new steroidal carboxylic acids, paraminabic acids A–C, from the coral *Paraminabea acronocephala*. However, only compound paraminabic acid C (**122**) (Figure 15) showed potent cytotoxicity toward the Hep3B, MDA-MB-231, MCF-7, and A-549 cancer cell lines, with IC_50_ values ranging from 2.05 to 2.83 μg/mL (4.81 to 6.64 µM). The positive control doxorubicin showed cytotoxicity against the Hep G2, Hep 3B, MDA-MB-231, MCF-7, and A549 cancer cell lines, with IC_50_ values of 0.31, 0.40, 1.32, 0.68, and 1.33 μg/mL (0.57, 0.74, 2.43, 1.25, and 2.45 µM), respectively. From the coral *Cladiella krempfi*, five new eunicellin-based diterpenoids and seven known compounds were isolated. Among them, 6-acetoxy litophynin E (**123**) and litophynin F (**124**) (Figure 15) exhibited effective cytotoxicity against the A549, BT483, H1299, HepG2, SAS, and BEAS2B cell lines, with IC_50_ values ranging from 4.8 ± 0.7 to 13.6 ± 0.5 μg/mL (11.8 ± 6.6 to 13.9 ± 1.3 µM), whereas krempfielin I and 6-methyl ether of litophynol B were less active [62]. In another study, two new cembrane-based diterpenoids, 11-acetylsinuflexolide and 11-acetyldihydrosinuflexolide, along with three known cembranoids (sinuflexolide, sinularin, and dihydrosinularin), were isolated from the coral *Sinularia flexibilis*. Interestingly, compound sinuflexolide (**125**) (Figure 15) exhibited cytotoxicity against the HeLa, HEp-2, MCF-7, and MDA-MB-231 cancer cell lines, with IC_50_ values of 8.6, 8.2, 16.0, and 11.3 μg/mL (24.4, 23.3, 45.5, and 32.1 µM), respectively, whereas the compounds 11-acetylsinuflexolide and sinularin were found to exhibit weak cytotoxicity toward some of the cell lines. According to a structure–activity relationship analysis of the five described cembrane-based diterpenoids, it seems that the α-exomethylenic–δ-lactone ring group in the compounds 11-acetylsinuflexolide, sinuflexolide, and sinularin is critical to the cytotoxic activity of the cembrane-based diterpenoids [110]. 

**Figure 16:**
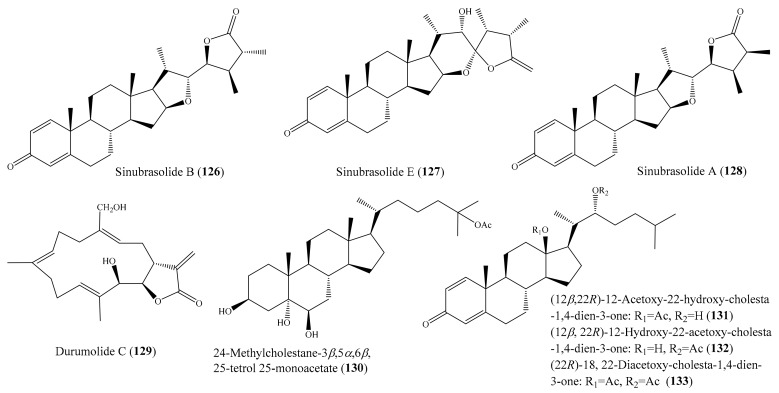
Chemical structures of **126**–**133**.

From the coral *Sinularia brassica*, seven novel withanolides, sinubrasolides A–G, were isolated. Among them, compound sinubrasolide B (**126**) (Figure 16) exhibited cytotoxicity toward the P388, MOLT 4, and HT-29 cancer cell lines, with ED_50_ values of 9.1 ± 1.4, 4.8 ± 0.9, and 4.8 ± 0.7 μM, respectively, while sinubrasolide E (**127**) (Figure 16) was found to show cytotoxicity toward MOLT-4 and HT-29, with ED_50_ values of 9.9 ± 1.8 and 7.5 ± 1.5 μM. In addition, sinubrasolide A (**128**) (Figure 16) showed cytotoxicity toward the K562 cell line, with an ED_50_ value of 8.7 ± 1.4 μM. The cytotoxicity of **126**–**128** was comparable to that of the positive control 5-fluorouracil toward P388, MOLT 4, K-562, and HT-29 cells, with ED_50_ values of 6.2 ± 0.8, 7.7 ± 0.8, 21 ± 2.0, and 7.7 ± 0.8 μM, respectively [111]. From the coral *Sinularia polydactyla*, three known terpenoides were isolated. Among them, the compound durumolide C (**129**) (Figure 16) showed strong cytotoxicity against the HepG2 cancer cell line, with an IC_50_ value of 1.0 µg/mL (3.0 μM), whereas the compound 24-methylcholestane-3β,5*α*,6β,25-tetrol 25-monoacetate (**130**) (Figure 16) showed strong cytotoxicity against the Hep2 and HCT-116 cancer cell lines, with IC_50_ values of 6.1 and 8.2 µg/mL (12.8 and 17.2 μM), respectively. The positive control doxorubicin exhibited cytotoxicity toward HepG2 cancer cells, with an IC_50_ value of 1.2 µg/mL (2.2 μM), whereas the positive control vinblastine exhibited cytotoxicity toward the HCT and Hep2 cancer cell lines, with IC_50_ values of 4.6 and 2.6 µg/mL (5.67 and 3.2 μM), respectively [112]. In another study, five new steroids ((12β, 22*R*)-12-acetoxy-22-hydroxy-cholesta-1,4-dien-3-one (**131**), (12β, 22*R*)-12-hydroxy-22-acetoxy-cholesta-1, 4-dien-3-one (**132**), (12β, 22*R*)-12, 22-diacetoxy-cholesta-1, 4-dien-3-one, (22*R*)-18, 22-diacetoxy-cholesta-1, 4-dien-3-one (**133**) (Figure 16), and (20*R*, 22*R*)-20-hydroxy-22-acetoxy-cholesta-1,4-dien-3-one), along with one known steroid (astrogorgol N), were isolated from the coral *Nephthea* sp. Although all of these compounds exhibited cytotoxic activity against the HeLa cell line, compounds **131**–**133** showed stronger activities, with IC_50_ values of 7.51 ± 0.22, 7.50 ± 0.31, and 8.29 ± 0.42 µg/mL (17.00 ± 0.50, 16.97 ± 0.70, and 18.50 ± 0.94 μM), respectively. After a comparison of the IC_50_ values of the compounds, it was suggested that the presence of a free hydroxyl group either at C-12 or C-22 was important for the enhancement of cytotoxic activity against HeLa cells. Moreover, it was observed that the introduction of a hydroxyl group at C-20 could decrease the inhibitory potency against HeLa cells, whereas the presence of an acetoxy group at C-18 seemed to enhance the cytotoxic activity [113].

**Figure 17:**
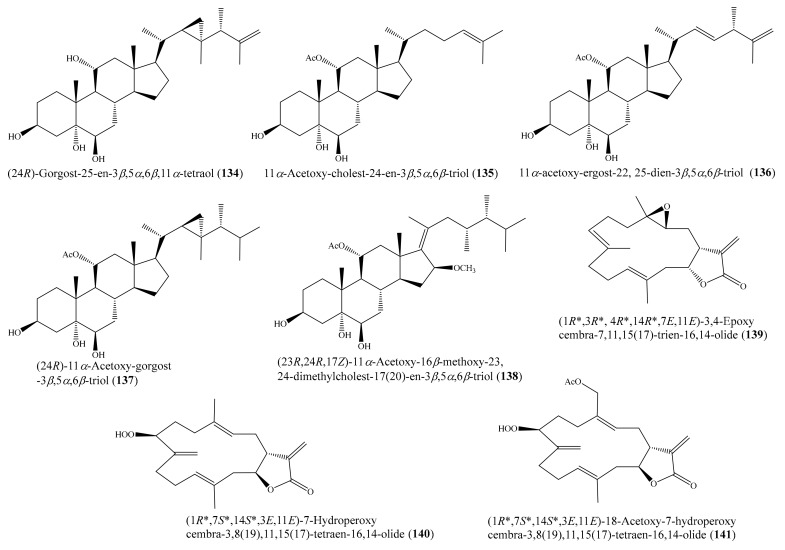
Chemical structures of **134**–**141**.

Chemical investigation of the coral *Sarcophyton* sp. led to the isolation of three new polyhydroxylated steroids, together with seven known steroids. Among them, the compounds (24*R*)-gorgost-25-en-3β,5*α*,6β,11*α*-tetraol (**134**), 11*α*-acetoxy-cholest-24-en-3β,5*α*,6β-triol (**135**), (22*E*,24*S*)-11*α*-acetoxy-ergost-22, 25-dien-3β,5*α*,6β-triol (**136**), and (24*R*)-11*α*-acetoxy-gorgost-3β,5*α*,6β-triol (**137**) (Figure 17) exhibited good cytotoxicity against the K562 cell line, with IC_50_ values of less than 10.0 μM, whereas the compound (23*R*,24*R*,17*Z*)-11*α*-acetoxy-16β-methoxy-23,24-dimethylcholest-17(20)-en-3β,5*α*,6β-triol (**138**) (Figure 17) displayed effective cytotoxicity toward the HL-60 tumor cell line, with an IC_50_ value of 9.3 μM. A comparative analysis of the structure–activity relationship of the isolated steroids suggested that the 11-OAc group is a very important pharmacophore. Furthermore, it was observed that the OH substitution at C-11 could improve the selectivity but decrease activity [114]. Zhao et al. [115] isolated three new *α*-methylene-*γ*-lactone-containing cembranoids ((1R*,3R*, 4R*,14R*,7*E*,11*E*)-3,4-epoxycembra-7,11,15(17)-trien-16,14-olide (**139**), (1R*,7S*,14S*,3*E*, 11*E*)-7-hydroperoxycembra-3,8(19),11,15(17)-tetraen-16,14-olide (**140**), and (1R*,7S*,14S*, 3*E*,11*E*)-18-acetoxy-7-hydroperoxycembra-3,8(19),11,15(17)-tetraen-16,14-olide (**141**)) (Figure 17) from the coral *Lobophytum* sp. Interestingly, the three new compounds exhibited remarkable cytotoxicity against the selected tumor cell lines (SGC7901, A549, MCF7, HCT-116, and B16), with IC_50_ values ranging from 1.2 to 8.6 μg/mL (4.0 to 27.2 μM).

**Figure 18:**
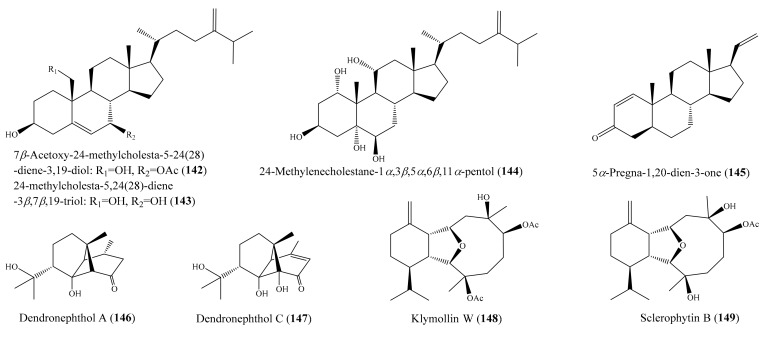
Chemical structures of **142**–**149**.

Chemical investigation of the coral *Klyxum molle* afforded 11 new eunicellin-based diterpenoids (see Section 2.1). However, only compound klymollin M (**38**) (Figure 5) exhibited strong cytotoxicity toward the K562, MOLT-4, and T47D cancer cell lines, with ED_50_ values of 7.97 ± 2.55, 4.35 ± 0.63, and 8.58 ± 1.72 μM, respectively, suggesting that eunicellin- based metabolites bearing a phenylacetate, as in **38**, may have enhanced cytotoxicity. Compound **38** exhibited stronger activity than the positive control 5-fluorouracil, which exhibited cytotoxic activity toward the K562, Molt-4, and T47D cancer cell lines, with ED_50_ values of 16.22 ± 1.77, 15.07 ± 1.61, and 50.20 ± 13.22 μM, respectively [66]. Ellithey et al. [116] reported the isolation of nine compounds from the coral *Litophyton arboreum*, of which the compounds 7β-acetoxy-24-methylcholesta-5-24 (28)-diene-3,19-diol (**142**) and 24-methylcholesta-5,24(28)-diene-3β,7β,19-triol (**143**) (Figure 18) were found to exhibit strong cytotoxicity against HeLa cells, with IC_50_ values of 4.3 ± 0.75 and 8 ± 0.5 μM, respectively. The cytotoxicity of **142** and **143** toward Hela cells was comparable to that of the positive control actinomycin D, with IC_50_ values of 5.1 ± 0.1 μM. In another study, Yen et al. [117] isolated a new sterol and two known sterols from the coral *Sinularia gaweli*. Notably, the compound 24-methylenecholestane-1*α*,3β,5*α*,6β,11*α*-pentol (**144**) (Figure 18) exhibited strong cytotoxicity toward the K562, MOLT-4, and HL-60 cell lines, with IC_50_ values of 9.71, 6.91, and 3.39 μg/mL, respectively (20.9, 14.9, and 7.3 μM). Three pregnane-type steroids, including a new metabolite along with two known analogues, were isolated from the coral *Scleronephthya flexilis*. Among them, only the compound 5*α*-pregna-1,20-dien-3-one (**145**) (Figure 18) exhibited potent activity against MOLT-4, HL-60, and K562 tumor cells, with IC_50_ values of 2.15, 3.14, and 8.32 μg/mL, respectively (7.21, 10.54, and 27.92 μM) [118]. From the coral *Dendronephthya* sp., three new ylangene-type sesquiterpenoids, dendronephthols A–C, were isolated. Interestingly, the compounds dendronephthol A (**146**) and dendronephthol C (**147**) (Figure 18) showed significant cytotoxic activity against the murine lymphoma L5187Y cancer cell line, with ED_50_ values of 8.4 and 6.8 µg/mL (33.3 and 25.6 μM), respectively [119]. In 2014, Chang et al. [120] isolated five new eunicellin-based diterpenoids, klymollins T–X, along with two known eunicellin-based diterpenoids, sclerophytin A and sclerophytin B, from the coral *Klyxum molle*. The compound klymollin W (**148**) (Figure 18) exhibited cytotoxicity toward the CCRF-CEM, MOLT-4, and T47D cancer cell lines, with ED_50_ values of 9.6, 8.5, and 19.9 μg/mL (22.7, 20.1, and 47.2 μM), respectively, whereas sclerophytin B (**149**) (Figure 18) showed cytotoxicity against the CCRF-CEM, K562, MOLT-4, and T47D cell lines, with ED_50_ values of 4.2, 15.0, 16.5, and 12.4 μg/mL (11.1, 39.5, 43.4, and 32.6 μM), respectively.

**Figure 19:**
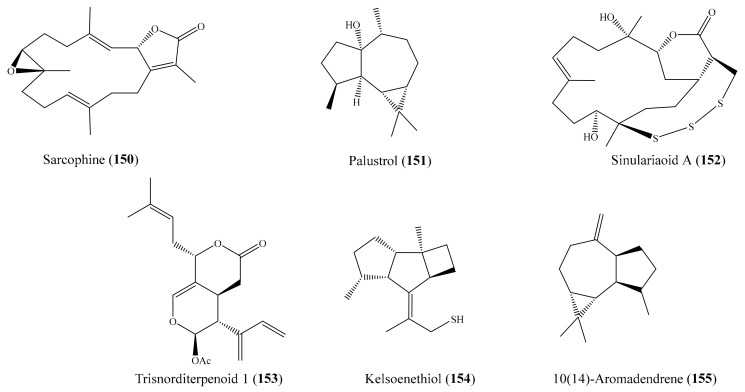
Chemical structures of **150**–**155**.

A subsequent investigation effort of the coral *Sarcophyton trocheliophorum* led to the isolation of two new rare pyrane-based cembranoids (sarcotrocheliol acetate and sarcotrocheliol), along with two known cembranoids (cembrene-C and sarcophine) and the aromadendrene sesquiterpenoid palustrol. Bioassays indicated that sarcophine (**150**) and palustrol (**151**) (Figure 19) recorded antitumor activities against lymphoma and Ehrlich cell lines, with an median lethal dose (LD_50_) in the range of 2.5–3.79 µM [121]. A chemical study of the coral *Sinularia* sp. yielded one novel nine-membered macrocyclic polysulfur cembranoid lactone, sinulariaoid A, and three new multioxygenated cembranoids, sinulariaoids B–D, together with four known cembranoids. Of these, sinulariaoid A (**152**) (Figure 19) exhibited the most potent in vitro anticancer activity, and its cytotoxicity in HepG2/ADM was more potent than in the other three cell lines (HepG2, MCF-7, and MCF-7/ADM), with an IC_50_ value of 9.70 ± 1.77 µM. Interestingly, the cytotoxic activity of **8** and **152** against the multidrug-resistant cell lines HepG2/ADM and MCF-7/ADM was comparable to that of the positive control doxorubicin. Furthermore, it was found that compound **152** induced apoptosis, and its selective toxicity toward HepG2/ADM cells was not related to P-glycoproteins [122]. In another chemical investigation, Roy et al. [123] reported the isolation of two new alcyonolide congeners, trisnorditerpenoid 1 and diterpenoid 2, from the coral *Cespitularia* sp. Interestingly, both compounds showed cytotoxicity against HCT-116 cells; however, trisnorditerpenoid 1 (**153**) (Figure 19) showed stronger activity, with an IC_50_ value of 6.04 µM. From another coral, *Nephthea erecta*, Cheng et al. [124] obtained two new kelsoane-type sesquiterpenes, kelsoenethiol and dikelsoenyl ether. Notably, only the compound kelsoenethiol (**154**) (Figure 19) showed cytotoxicity against P-388 and HT-29 cells, with ED_50_ values of 1.3 and 1.8 µg/mL (5.5 and 7.6 μM), respectively. It was noted that the mercaptan group at C-12 exhibited cytotoxic activity against P-388 and HT-29 cells more potently than kelsoene did, suggesting that the presence of the C-12 mercaptan group is important for cytotoxicity against P-388 and HT-29 cells. In another effort, Al-Lihaibi et al. [125] isolated three new cembranoids (sarcophytolol, sarcophytolide B, and sarcophytolide C) along with three known metabolites (10(14)aromadendrene, deoxosarcophine, and sarcophine) from the coral *Sarcophyton glaucum*. Among them, compound 10(14)-aromadendrene (**155**) (Figure 19) showed potent cytotoxicity activity toward PC-3, with an IC_50_ value of 9.3 ± 0.164 µM, whereas the compounds sarcophytolol and sarcophytolide C had similar moderate cytotoxic effects toward the HepG2 cell line, with an IC_50_ value of 20 µM. Furthermore, the compounds sarcophytolide B and C showed moderate activity against the MCF-7 cell line, with IC_50_ values of 25 ± 0.0164 and 29 ± 0.030 µM, respectively. The antiproliferative activity of sarcophytolol, sarcophytolide C, and **155** could be attributed, at least partly, to their ability to induce cellular apoptosis. Interestingly, all of the metabolites showed no cytotoxicity against normal adult African green monkey kidney (VERO) cells even at 100 mM. From the coral *Sarcophyton crassocaule*, three new cembranoids (**46**–**48**) and four known compounds (**49**–**52**) (Figure 6) were isolated (see Section 2.1). Of these, compounds **48**–**50** and **52** were found to exhibit strong cytotoxicity toward all or part of the carcinoma cell lines DLD-1, CCRF-CEM, and HL-60, with ED_50_ values ranging from 3.8 to 11.1 µM [72].

**Figure 20:**
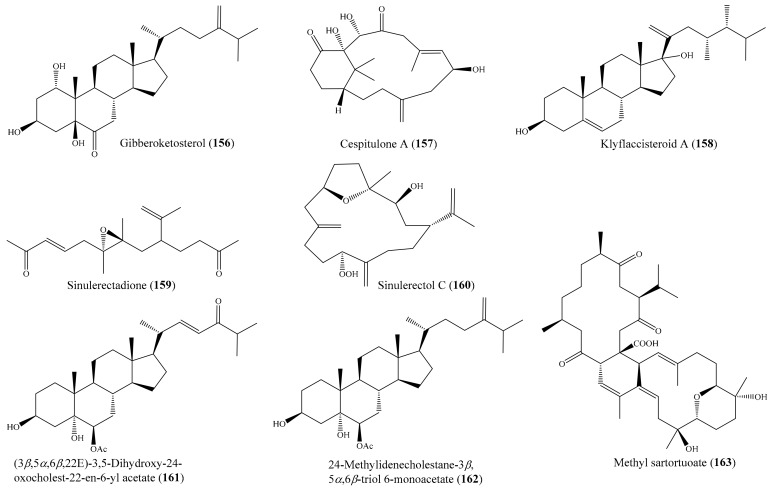
Chemical structures of **156**–**163**.

From the coral *Sinularia numerosa,* four new cembrane-type diterpenes, numerosols A–D, along with a known steroid, gibberoketosterol (**156**) (Figure 20), were isolated. Interestingly, only **156** exhibited cytotoxicity against the P-388 cell line, with an ED_50_ of 6.9 μM [126]. In another study, Lin et al. [127] isolated two novel diterpenoids, cespitulones A and B, which possess an unprecedented bicyclo ring system with C-C bond connections between C-10 and C-20 and between C-20 and C-11, from the coral *Cespitularia taeniata*. However, only the compound cespitulone A (**157**) (Figure 20) exhibited significant cytotoxicity against the Daoy and WiDr cancer cell lines, with IC_50_ values of 8.7 and 6.7 µM, respectively. Chemical investigation of the coral *Klyxum flaccidum* afforded six new steroids, klyflaccisteroids A–E, and a new 9,11-secogorgosteroid, klyflaccisteroid F, along with two known steroids and a known eunicellin-based diterpenoid (see Section 2.1). The compound klyflaccisteroid A (**158**) (Figure 20) could selectively inhibit the growth of A549 cancer cells, with an ED_50_ value of 7.7 µg/mL (18.0 µM), whereas the compound klyflaccisteroid E (**61**) exhibited the strongest cytotoxicity toward the HT-29 cancer cell line, with an ED_50_ of 6.9 µg/mL (15.1 µM). In addition, the klyflaccisteroids C and E (**58** and **61**) (Figure 7) were found to be the most toxic toward A549 and P388 cells, with ED_50_ values of 6.1 and 3.7 µg/mL (13.4 and 8.1 µM), respectively. Interestingly, compounds **58** and **158** showed stronger activity against the A549 cell line than the positive control 5-fluorouracil (ED_50_ = 14.3 µg/mL or 109.9 µM) did [75]. From the coral *Sinularia erecta*, four new isoprenoids, along with three known isoprenoids and a known nitrogen-containing compound, were isolated (see Section 2.1). The cytotoxic assay showed that the compound sinulerectadione (**159**) (Figure 20) exhibited cytotoxicity toward the K-562 and MOLT-4 cancer cell lines, with IC_50_ values of 8.6 ± 1.1 and 9.7 ± 2.9 μM, respectively, while sinulerectol C (**160**) (Figure 20) showed cytotoxicity toward the K-562 cell line, with an IC_50_ value of 9.2 ± 3.3 μM. In addition, (*Z*)-*N*-[2-(4-hydroxyphenyl)ethyl]-3-methyldodec-2-enamide (**57**) was found to show cytotoxicity toward CCRF-CEM and MOLT-4, with IC_50_ values of 6.3 ± 1.5 and 9.7 ± 3.6 μM, respectively. Notably, the cytotoxic activity of **159** and **160** against the K-562 cancer cell line was stronger than that of the positive control 5-fluorouracil (IC_50_ = 33 ± 9 μM), and the cytotoxic activity of **157** against the CCRF-CEM (leukemia) cancer cell line was stronger than that of the positive control (IC_50_ = 17 ± 5 μM) [74]. Furthermore, from the coral *Sarcophyton glaucum,* two new biscembranes, glaucumolides A and B (**62**, **63**) (Figure 7), along with two known metabolites, were isolated (see Section 2.1), of which both new compounds, **62** and **63**, exhibited cytotoxicity against the HL-60, CCRF-CEM, MOLT-4, and K562 cancer cell lines, with ED_50_ values ranging from 3.8 to 19.2 µg/mL (5.5 to 27.8 µM). The cytotoxic activity of **62** and **63** against the HL-60 cancer cell line was stronger than that of the control 5-fluorouracil (IC_50_ = 10.7 ± 0.5 µg/mL or 82.3 ± 3.8 µM) [76]. In another study, four new polyhydroxylated steroids together with six known compounds were isolated from the coral *Sinularia acuta*. The compounds (3β,5*α*,6β,22*E*)-3,5-dihydroxy-24-oxocholest-22-en-6-yl acetate (**161**) and 24-methylidenecholestane-3β,5*α*,6β-triol 6-monoacetate (**162**) (Figure 20) showed potent cytotoxicity against HL-60 cell lines, with IC_50_ values of 7.3 and 9.9 µM, respectively, whereas the compounds 24-methylidenecholestane-3β,5*α*,6β-triol 6-monoacetate and (24*S*)-methylidenecholestane-3β,5*α*,6β-triol 6-monoacetate showed moderate activity against K562 cell lines, with IC_50_ values of 10.9 and 11.7 µM, respectively. Furthermore, **161** and (24*S*)-methylidenecholestane-3β,5*α*,6β-triol 6-monoacetate showed weak activity against HeLa cell lines, with respective IC_50_ values of 44.8, 27.1, and 18.2 µM [128]. The structure–activity comparison suggested that the 3b,5a,6b-triol pattern could be a critical pharmacophore for steroids and that the side chain also plays an important role. This structure–activity relationship assumption was consistent with a recent study on the polyhydroxylated steroids from another soft coral, *Sarcophyton* sp. [114]. In addition, from the coral *Sarcophyton pauciplicatum*, two new biscembranoids, sarcophytolides M and N, together with eight known ones, were isolated. Among them, sarcophytolide M, sarcophytolide I, sarcophytolide J, lobophytone U, methyl tortuoate B, and methyl sartortuoate exhibited cytotoxic effects against all or part of eight human cancer cell lines, including HepG2, HL-60, KB, LU-1, MCF7, SK-Mel2, and SW480. Notably, methyl sartortuoate (**163**) (Figure 20) exhibited strong cytotoxicity toward the HL-60 cell line, with an IC_50_ value of 7.93 ± 2.08 µM [129].

**Figure 21:**
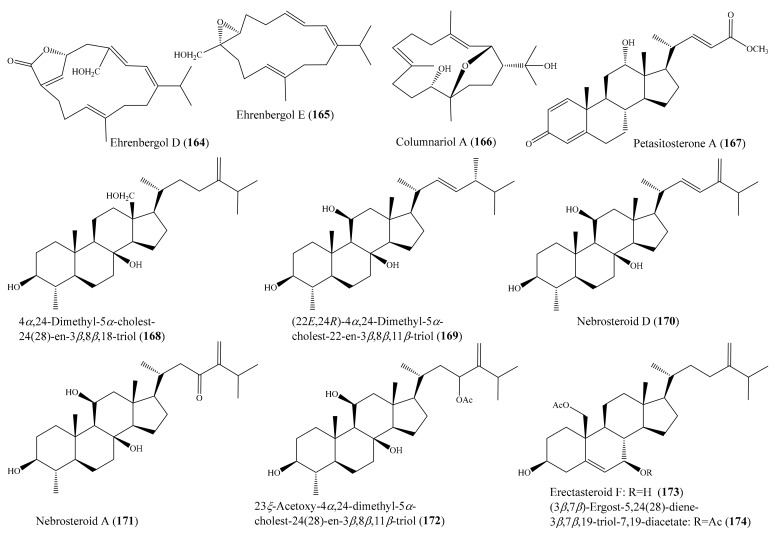
Chemical structures of **164**–**174**.

From the coral *Sarcophyton ehrenbergi*, five new polyoxygenated cembranoids were obtained, of which compounds ehrenbergols D (**164**) and E (**165**) (Figure 21) showed significant cytotoxicity against the P-388 cancer cell line, with EC_50_ values of 2.0 and 3.0 μM, respectively, whereas three remaining polyoxygenated cembranoids showed no cytotoxicity. The structure–activity comparison of the cembranoids suggested that the conjugated double bonds at C-1/C-2 and C-3/C-4 were important for cytotoxicity against P-388 cells [130]. In another study, two new cembranes, columnariols A and B, were isolated from the coral *Nephthea columnaris*; however, only compound columnariol A (**166**) (Figure 21) exhibited moderate cytotoxicity toward LNCaP cells, with an IC_50_ value of 9.80 μg/mL (30.4 µM) [131]. Chemical investigation of the coral *Umbellulifera petasites* led to the isolation of three new steroids, along with eight known steroids (see Section 2.1). Among them, compound petasitosterones A–C (**167**, **68**, **70**) (Figure 8 and Figure 21) and 5*α*-pregna-1,20-dien-3-one (**69**) (Figure 8) displayed inhibitory activity against the proliferation of a limited panel of cancer cell lines (K-562, MOLT-4, and DLD-1), with IC_50_ values ranging from 5.8 ± 1.7 to 15.2 ± 3.5 µg/mL (19.5 ± 5.7 to 33.3 ± 7.7 µM) [79]. From the coral *Litophyton mollis*, seven new 4*α*-methylated steroids and three known steroids were isolated. Of these, 4*α*,24-dimethyl-5*α*-cholest-24(28)-en-3β,8β,18-triol (**168**), (22*E*,24*R*)-4*α*,24-dimethyl-5*α*-cholest-22-en-3β,8β,11β-triol (**169**), nebrosteroid D (**170**), nebrosteroid A (**171**), and 23*ξ*-acetoxy-4*α*,24-dimethyl-5*α*-cholest-24(28)-en-3β,8β,11βtriol (**172**) (Figure 21) exhibited strong cytotoxic activity against K562 human chronic myelogenous leukemia cells, with IC_50_ values less than 10 µM, while at the same time they displayed low toxicity against healthy peripheral blood mononuclear cells. Furthermore, these compounds displayed moderate levels of toxicity against A549 cells, with IC_50_ values above 10 µM [132]. In another study, Tsai et al. [133] isolated a new 10-demethylated steroid, a new 19-oxygenated steroid, and five known steroids from the coral *Nephthea erecta*. Interestingly, the compounds erectasteroid F (**173**) and (3β,7β)-ergost-5,24(28)-diene-3β,7β,19-triol 7,19-diacetate (**174**) (Figure 21) exhibited effective cytotoxicity against K562, Molt-4, Sup-T1, and U937 cell lines, with IC_50_ values of 6.5–14.0 μM, while 24-methyl-cholesta-5,24(28)-diene-3β,19-diol-7β-monoacetate showed moderate cytotoxicity against K562, MOLT-4, and Sup-T1 cell lines, with IC_50_ values of 11.2–19.9 μM. A preliminary structure–activity relationship of oxygenated steroids could be deduced from cytotoxicity bioassays. It was found that the presence of the acetoxy or hydroxy functional group at C-7 was critical for the cytotoxicity action of this class of compounds.

**Figure 22:**
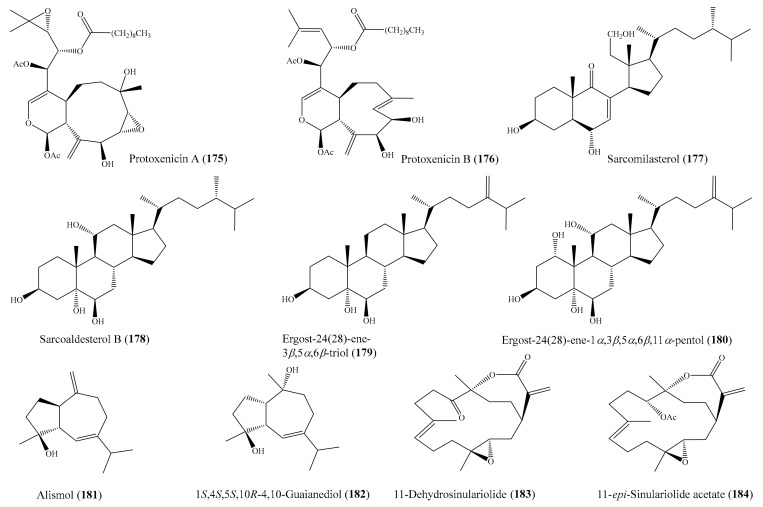
Chemical structures of **175**–**184**.

From the coral *Pinnigorgia* sp., three novel 9,11-secosterols, pinnigorgiols A–C (**74**-**76**) (Figure 9), were isolated (see Section 2.1), of which **74** and **75** decreased the cell viability of the HSC-T6 cell line, with IC_50_ values of 5.77 ± 0.27 and 7.89 ± 0.52 µM, respectively. However, compound **76** displayed no effect on the same cell line, suggesting that the configuration of C-7 influenced the bioactivity [82]. Urda et al. [134] isolated two new xenicanes, protoxenicins A (**175**) and B (**176**) (Figure 22), from the coral *Protodendron repens*. Interestingly, both of the **175** and **176** xenicanes displayed significant cytotoxic activity against the NSLC A-549, HT-29, and MDA-MB-231 (breast) cancer cell lines, with GI_50_ values of 0.6–6.3 μM. Doxorubicin was used as a positive control, with GI_50_ values of 0.2, 0.3, and 0.2 μM, against MDA-MB-231, HT-29, and NSLC A549, respectively. Chemical investigation of the coral *Sarcophyton glaucum* afforded 5 new isoprenoids along with 10 known compounds. Among them, the compound sarcomilasterol (**177**) (Figure 22) exhibited cytotoxicity effects against the MDA-MB-231, MOLT-4, SUP-T, and U-937 cell lines, with IC_50_ values of 6.7–17.7 µg/mL (15.0–39.5 µM), while the compound sarcoaldesterol B (**178**) (Figure 22) also showed cytotoxicity against the HepG2, MDA-MB-231, and A-549 cell lines, with IC_50_ values of 9.7–15.8 µg/mL (21.6–35.1 µM) [135]. In another study, Mohammed et al. [136] isolated five sterols and three sesquiterpenes from the coral *Sinularia terspilli*, of which the compounds ergost-24(28)-ene-3β,5*α*,6β-triol (**179**), ergost-24(28)-ene-1*α*,3β,5*α*,6β,11*α* pentol (**180**), alismol (**181**), and (1*S*,4*S*,5*S*,10*R*)-4,10-guaianediol (**182**) (Figure 22) exhibited potent cytotoxic activity against the HL60 and K562 cell lines, with IC_50_ values ranging from 0.002 ± 0.001 to 0.60 ± 0.08 μM, respectively. It was noted that the compounds **179** and **180** exhibited strong cytotoxic activity against HL60 and K562 cells, with IC_50_ values of 0.002–0.004 and 0.003–0.005 µM, respectively, compared to the positive control taxol, which showed IC_50_ values of 0.0005 and 0.0023 µM against HL60 and K562, respectively. From the coral *Sinularia flexibilis*, five new cembranoid-related diterpenoids, along with nine known compounds, were isolated. A bioassay showed that the compounds 11-dehydrosinulariolide (**183**), 11-*epi*-sinulariolide acetate (**184**) (Figure 22), 14-deoxycrassin, and sinulariolide exhibited cytotoxic activity toward the P-388 and K-562 cancer cell lines, with IC_50_ values ranging from 6.9 µM to 26.7 µM. Notably, compound **183** showed selective cytotoxicity toward P-388, with an IC_50_ value of 9.3 µM, while compound **184** was found to show potent activity and selectivity toward the P-388 and HT-29 cancer cell lines, with IC_50_ values of 6.9 and 9.6 µM, respectively [137]. Compound 5-dehydrosinulariolide (=11-dehydrosinulariolide) (**183**) was also found to display cytotoxicity against the A549, HT-29, SNU-398, and Capan-1 cancer cell lines, with IC_50_ values ranging from 8.9 to 27.4 μM [84]. It was noted that among all of the nine tested cembranoids (Wu et al. [84]), only those containing a seven-membered lactone exhibited cytotoxicity, suggesting that the *α*, β-unsaturated seven-membered lactone might have been responsible for the activity. Moreover, compound **183** (Figure 22), with a carbonyl group at the C-5 position, displayed obviously stronger activity than **78** and **79** (Figure 9), whose structures only differed from **183** at the C-5 position, suggesting that the carbonyl at C-5 instead of the hydroxyl or acetoxyl group improved the cytotoxic activity [84].

### 2.3. Antimicrobial Compounds from Coral

The emergence of antimicrobial resistance to existing antibiotics, the prevalence of multidrug-resistant bacteria, and the rapid development of cross-resistance with new drugs have become a serious public health problem. Antibiotic resistance is able to lead to high mortality and consequently impose huge healthcare costs [138,139]. Antibiotic-resistant pathogens can be acquired through mutation or horizontal transfer of a resistant gene [140], therefore compromising the effect of antibiotics [141]. Attempts to modify existing drugs are often not effective enough to overcome the mutation rate of pathogens and do not result in the introduction of new classes of antimicrobial agents [142]. In addition, the isolation of replicated known compounds significantly reduces the discovery rate of new antimicrobial compounds. Hence, efforts are aimed at discovering and producing efficient novel antibiotics [143]. The exploration of unexplored habitats and uncommon environments has become important for discovering novel therapeutic agents.

**Figure 23:**
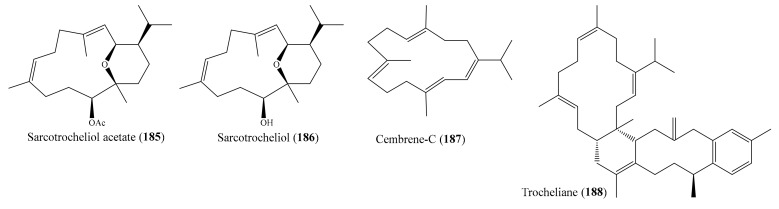
Chemical structures of **185**–**188**.

Antimicrobial resistance around the globe has led to an emerging demand for searches for novel antimicrobial drugs from marine environments. Recent investigations of corals have shown that many antimicrobial compounds have been isolated from coral (Supplementary Table S3): in particular, some compounds have exhibited strong activity against multiple pathogenic microorganisms. For example, two new rare pyrane-based cembranoids along with two known cembranoids and the aromadendrene sesquiterpenoid palustrol were isolated from the coral *Sarcophyton trocheliophorum*. Of these, the compounds sarcotrocheliol acetate (**185**) and sarcotrocheliol (**186**) (Figure 23) displayed potent antibacterial activity, especially against *Staphylococcus aureus*, *Acinetobacter* spp., and methicillin-resistant *Staphylococcus aureus* (MRSA), with minimal inhibitory concentrations (MICs) ranging from 1.53 to 4.34 µM, while cembrene-C (**187**) (Figure 23) exhibited potent antifungal activity against *Aspergillus flavus* and *Candida albicans*, with an MIC value of 0.68 µM. Furthermore, the compounds sarcophine (**150**) and palustrol (**151**) (Figure 19) showed lower antibacterial and antifungal activities compared to compounds **185**–**187**. The antibacterial activity of **185** and **186** was comparable to that of the positive control ampicillin, whereas the antifungal activity of **187** against *Aspergillus flavus* and *Candida albicans* was stronger than that of the positive control amphotericin B (MIC = 4.6 µM) [121]. From the coral *Sarcophyton trocheliophorum*, Zubair et al. [144] also isolated a new tetracyclic biscembrane hydrocarbon, trocheliane, along with two new cembranoid diterpenes, sarcotrocheldiols A and B. All of these new compounds showed antimicrobial activity, of which trocheliane (**188**) (Figure 23) exhibited strong antimicrobial activity against multiple reference microorganisms (*Acinetobacter baumannii*, *Escherichia coli*, *Klebsiella pneumonia*, *Pseudomonas aeruginosa*, *Staphylococcus aureus*, *Staphylococcus epidermidis*, and *Streptococcus pneumoniae*), with MIC values ranging from 4 to 6 µM. The antimicrobial activity of **188** was comparable to that of the positive control ampicillin, with MIC values ranging from 2.6 to 13 µM.

### 2.4. Antivirus Compounds from Coral

Viruses are defined as a prodigious group of microorganisms causing assorted infectious diseases. Recent studies have reported the discovery of new antiviral agents, which generally inhibit the virus replication cycle by affecting the host cell factors for virus replication and/or viral elements [145]. Despite the development of antiviral agents over the past decades, patients suffering from viral infections are seriously influenced by treatment failure due to the emergence of recombinant viruses, drug resistance, and cell toxicity [146,147,148]. The high virulence of viruses and the lack of effective therapies have become a serious threat to public health. Moreover, the widespread occurrence of chronic viral infectious diseases and the rapid evolution and resistance of viruses to existing pharmaceuticals require ongoing exploration of new antivirus agents with higher efficiency and fewer side effects.

**Figure 24:**
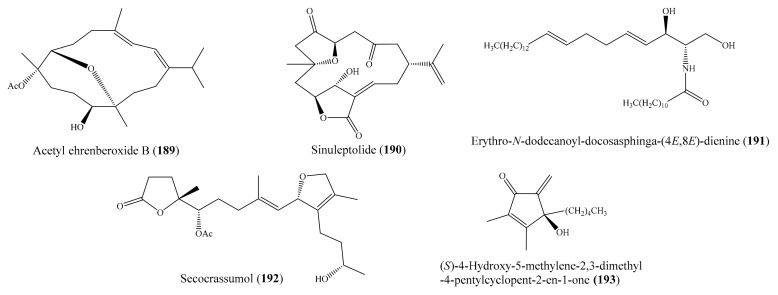
Chemical structures of **189**–**193**.

Besides antimicrobial activity, many compounds isolated from coral possess antivirus activity against different viruses (e.g., HCMV, H1N1, HIV-1) (Supplementary Table S4). Interestingly, several compounds show potent antiviral activity, indicating them as potential antiviral drugs. For example, three new cembranoids (**108**–**110**) (Figure 14) were isolated from the coral *Sarcophyton ehrenbergi* (see Section 2.2). Interestingly, ehrenbergol B (**110**) displayed strong antiviral activity against the human cytomegalovirus (HCMV), with an IC_50_ value of 5 μg/mL (13.2 µM), while the two remaining compounds, **108** and **109**, displayed weak antiviral activity against human cytomegalovirus, with IC_50_ values of 60 and 46 μg/mL (160.4 and 132.2 µM), respectively [104]. From the same coral species*,* two new diterpenoids, ehrenbergol C and acetyl ehrenberoxide B (**189**) (Figure 24), were also isolated. Notably, both of these compounds displayed strong antiviral activity toward the human cytomegalovirus, with an EC_50_ of 20 and 8.0 μg/mL (54.9 and 22.0 µM), respectively [149]. In another study, Tseng et al. [150] reported the isolation of two new 9,11-secosteroids and two known norcembranoids from the coral *Sinularia nanolobata*; however, only sinuleptolide (**190**) (Figure 24) showed anti-HCMV activity, with an ED_50_ value of 1.92 μg/mL (5.5 µM). Ganciclovir was used as a positive control, with an ED_50_ of 0.12 μg/mL (0.47 µM). From the coral *Litophyton arboreum,* Ellithey et al. [116] also isolated nine compounds (see Section 2.2). Of these, the compounds alismol (**181**) (Figure 22), 7β-acetoxy-24-methylcholesta-5-24(28)-diene-3,19-diol (**142**) (Figure 18), and erythro-*N*-dodecanoyl-docosasphinga-(4*E*,8*E*)-dienine (**191**) (Figure 24) showed strong inhibitory activity against the HIV-1 PR virus, with IC_50_ values of 7.20 ± 0.7, 4.85 ± 0.18, and 4.80 ± 0.92 μM, respectively. Chemical investigation of the coral *Lobophytum crassum* led to the isolation of a new *seco*-cembranoid, secocrassumol (**192**) (Figure 24). Interestingly, this compound showed strong antiviral activity against the human cytomegalovirus (HCMV), with an IC_50_ value of 5.0 μg/mL (12.7 µM) [151]. From the coral *Sinularia verruca,* 19 metabolites with diverse structures were isolated. Among them, compound (*S*)-4-hydroxy-5-methylene-2,3-dimethyl-4-pentylcyclopent-2-en-1-one (**193**) (Figure 24) showed effective protection against the cytopathic effects of HIV-1 infection, with EC_50_ values of 5.8–34 μM [152].

### 2.5. Antifouling Compounds from Coral

Biofouling is defined as the undesirable growth of microorganisms (e.g., bacteria, algae, and protozoa) and macroorganisms (e.g., barnacles, bryozoans, and tubeworms) on submerged surfaces [153]. Biofouling not only causes huge losses in marine technical and economic fields but also results in a series of environmental problems, such as the spread of invasive species [153,154,155]. The main strategies for the control of biofouling include the use of paints containing materials (copper, lead, mercury, arsenic, and organotins) or antifouling compounds to coat the substrata [156,157]. However, these antifouling compounds have raised many environmental issues because of their toxicities toward nontarget organisms [158]. For example, organotin compounds and alternative antifouling biocides such as Irgarol 1051 and diuron have been prohibited by the International Maritime Organization and many European countries because of the increasing evidence of their environmental risks [159,160]. Thus, there is urgent demand for a search for novel antifouling compounds with high efficiency and low/nontoxicity.

**Figure 25:**
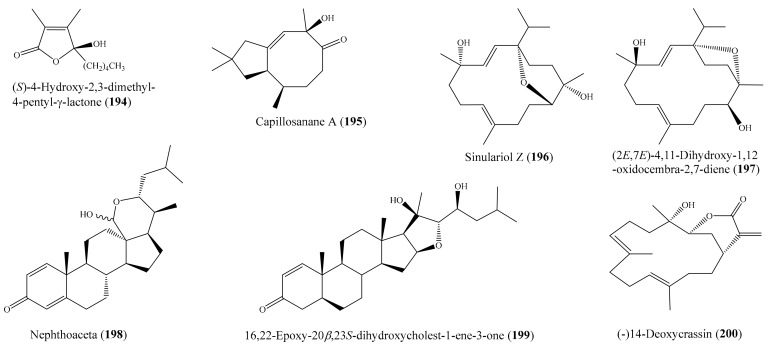
Chemical structures of **194**–**200**.

The chemical and bioactive investigation of coral has resulted in the isolation of antifouling compounds (Supplementary Table S5), of which several compounds exhibit potent activities. From the coral *Sinularia* sp., six new cyclopentenone derivatives, sinularones A–F, and three new furanones, sinularones G–I, together with a known butanolide, were isolated. Compound (*S*)-4-hydroxy-2,3-dimethyl-4-pentyl-*γ*-lactone (**194**) (Figure 25) showed potent antifouling activities against the barnacle *Balanus amphitrite*, with an EC_50_ of 3.84 μg/mL (19.4 µM), whereas the compound sinularones A, B, and F–I showed moderate antifouling activities against the barnacle *Balanus amphitrite*, with EC_50_ values > 10 μg/mL. It was noted that the compounds sinularone A and B and sinularones G–I showed potent inhibition, with EC_50_ values lower than the standard requirement (EC_50_ < 25 μg/mL), with regard to the efficacy level of natural antifouling agents as established by the U.S. Navy program [161]. A primary discussion of the structure–activity relationship suggested that α,β-unsaturated 2,3-dimethyl-γ-lactone is a functional unit for anti-barnacle [162]. In another study, 14 new asteriscane-type sesquiterpenoids, capillosananes A–N; four new *seco*-asteriscanes, capillosananes O–R; and (-)-sinularone A and sinularone A were obtained from the coral *Sinularia capillosa*. Interestingly, capillosanane A (**195**) (Figure 25) exhibited potent antifouling activity against *Balanus amphitrite*, with an IC_50_ value of 9.70 μM. In addition, it was found that the configuration at C-9 may play a key role in antifouling activity [163]. A subsequent chemical investigation of the coral *Sinularia rigida* afforded 12 new cembranoids, sinulariols T–Z and Z_1_–Z_5_, together with a known analogue. Among them, the compounds sinulariol Z (**196**) and (2*E*,7*E*)-4,11-dihydroxy-1,12-oxidocembra-2,7-diene (**197**) (Figure 25) showed potent antifouling activity against the barnacle *Balanus amphitrite*, with IC_50_ values of 4.57 and 4.86 μg/mL (14.2 and 15.1 µM), and moderate inhibition against *Bugula neritina*, with IC_50_ values of 12.34 and 13.48 μg/mL (38.3 and 41.9 µM), respectively. Analyses of the primary structure–activity relationship revealed functional groups and olefinic geometry affecting antifouling activity. Active compound **197** was a C-19 dehydroxylated sinulariol D, whereas sinulariol D and its C-4 epimer sinulariol F exhibited weak inhibitory activity against *B. amphitrite* and *Bu. neritina*, suggesting that OH-19 dramatically decreased the inhibition in a 1,12-epoxy pattern. The similar data were observed in comparison to the antifouling activity of **196** and sinulariols H and J. With 11,12-epoxy analogues, sinulariol P exhibited stronger inhibitory activity against *B. neritina* than sinulariols L and O did, indicating that the 7E geometry was better than 7Z [164]. A novel unusual pentacyclic hemiacetal sterol, nephthoaceta, along with two unusual pentacyclic steroids, were also isolated from the coral *Nephthea* sp. A biological examination indicated that the compound nephthoaceta (**198**) (Figure 25) exhibited significant antifouling against *Bugula neritina* larvae, with an EC_50_ value of 2.5 µg/mL (6.3 µM). Compared to **198**, its two acetyl derivatives possessed an additional acetyl group on the tetrahydropyran ring, and their huge differences in antifouling activity compared to **198** revealed that the free hydroxyl group at the acetal carbon C-18 may have been very important in the observed antifouling activity [165]. In another study, Zhang et al. [166] isolated two new unusual cholestane derivatives along with two new pregnane derivatives from the coral *Subergorgia suberosa*. Interestingly, compounds 16,22-epoxy-20β,23*S*-dihydroxycholest-1-ene-3-one (**199**) (Figure 25) and 20β,23*S*-dihydroxycholest-1-ene-3,22-dione exhibited antifouling activity against *Balanus amphitrite* larvae settlement, with EC_50_ values of 5.3 and 14.5 µg/mL (12.3 and 33.7 µM), respectively, whereas two new pregnane derivatives, 15b,17a-dihydroxypregna-4,6-diene-3,20-dione and 11a-hydroxypregna-4-ene-3,6,20-trione, showed no antifouling activity at a concentration of 25 µg/mL, an efficacy level for natural antifoulants set by the U.S. Navy program. Compared to compound 20β,23*S*-dihydroxycholest-1-ene-3,22-dione, compound **199** possesses a tetrahydrofuran moiety, which might be important for its potent inhibitory activity against the settlement of *B. amphitrite* larvae. Interestingly, this assumption is in good agreement with a previous report, where the furan moiety was indicated to be an important functional group responsible for antifouling activity [167]. Wang et al. [168] also isolated seven cembrane diterpenes from the coral *Sinularia flexibilis*. It is notable that all compounds except sinularin showed antifouling activity. In particular, compound (−)14-deoxycrassin (**200**) (Figure 25) exhibited remarkable antifouling activity against the two biofoulers *B. amphitrite* and *B. albicostatus*, with EC_50_ values of 3.90 and 21.26 µg/mL (12.3 and 66.9 µM), respectively.

### 2.6. Other Bioactive Compounds from Coral

**Figure 26:**
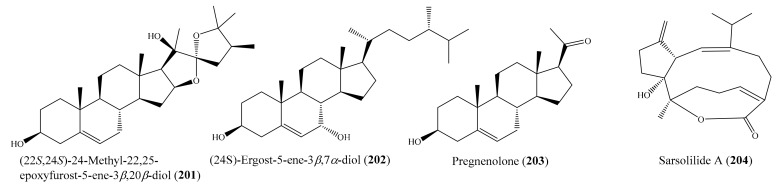
Chemical structure of **201**–**204**.

Besides the above biological activities mentioned, coral-derived compounds show other bioactivities, such as acetylcholinesterase inhibitory, polymerase proofreading-associated polyposis (PPAP) transcriptional, heme polymerization inhibitory, PTP 1B inhibitory, and DPPH radical scavenging activities (Supplementary Table S6). For example, from the coral *Lobophytum laevigatum*, a new unusual sterol, lobophytosterol, and five known metabolites were isolated. Among them, compounds (22*S*,24*S*)-24-methyl-22,25-epoxyfurost-5-ene-3β,20β-diol (**201**), (24*S*)-ergost-5-ene-3β,7*α*-diol (**202**), and pregnenolone (**203**) (Figure 26) significantly upregulated PPAR transcriptional activity dose-dependently in Hep-G2 cells. At a concentration of 1 μM, compounds **201**–**203** enhanced PPAR transcriptional activity by 1.4-, 1.2-, and 1.6-fold, respectively [169]. Chemical investigation of the coral *Sarcophyton trocheliophorum* Marenzeller yielded two new sarsolenane diterpenes and two new capnosane diterpenes, together with the known analogue. Of these, compounds sarsolilide A (**204**) (Figure 26) and sarsolilide B exhibited inhibitory activity against protein tyrosine phosphatase 1B (PTP1B), with IC_50_ values of 6.8 ± 0.96 and 27.1 ± 2.6 µM, respectively, representing the first report of PTP1B inhibitory activity for capnosane diterpenes. Interestingly, compound **204** showed remarkable inhibitory activity similar to that of the positive control (oleanolic acid), with an IC_50_ of 2.6 ± 0.4 µM [170].

## 3. Bioactive Compounds from Coral-Associated Microorganisms

### 3.1. Anti-Inflammatory and Cytotoxic Compounds from Coral-Associated Microorganisms

Coral harbors a high diversity of associated microorganisms, and many coral-associated microorganisms are found to produce anti-inflammatory and cytotoxic compounds (Supplementary Table S7). Several of them show potent activity, presenting as promising compounds for the development of new anti-inflammatory and cytotoxic agents.

**Figure 27:**
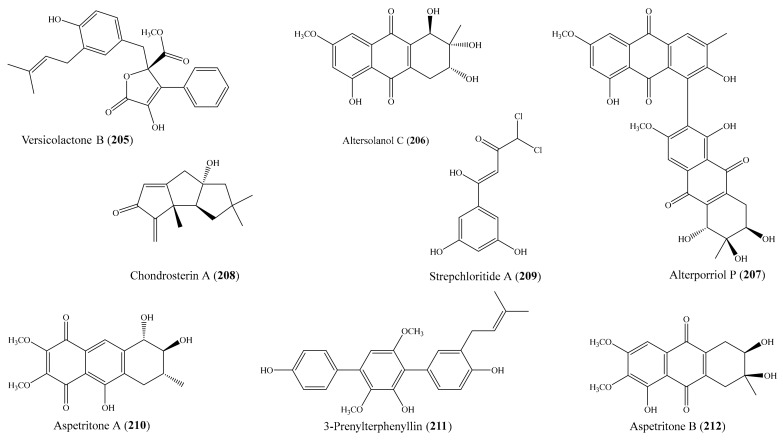
Chemical structures of **205**–**212**.

From the coral-derived fungus *Aspergillus terreus*, four new butenolide derivatives and six known ones were obtained. Of these, compounds versicolactone B (**205**) (Figure 27), 3′-isoamylene butyrolactone IV, and butyrolactone I showed anti-inflammatory activity by inhibiting NO production in LPS-induced RAW 264.7 mouse macrophages. In particular, **205** exhibited an even stronger inhibition of NO production than the positive control indomethacin, presenting itself as a promising lead compound for the development of new anti-inflammatory agents [171].

From *Alternaria* sp. ZJ-2008003, a fungus obtained from soft coral *Sarcophyton* sp., five new hydroanthraquinone derivatives, tetrahydroaltersolanols C–F and dihydroaltersolanol A; five new alterporriol-type anthranoid dimers, alterporriols N–R; along with seven known analogues were isolated. Interestingly, the anthraquinone derivative altersolanol C (**206**) (Figure 27) exhibited strong cytotoxicity against five cancer cell lines (HCT-116, MCF-7/ADR, PC-3, HepG2, and Hep3B), with IC_50_ values between 2.2 and 8.9 μM, whereas the hydroanthraquinone derivatives, tetrahydroaltersolanols C–F, dihydroaltersolanols A and B, altersolanol B, altersolanol L, and ampelanol, with an oxidized C-10 and a reduced C-9 fragment, were inactive. These results suggested that the paraquinone moiety was important for cytotoxic activity. The cytotoxic activity of **206** against HCT-16 and MCF-7/ADR cells was comparable to that of the positive control epirubicin (IC_50_ values of 0.82 and 1.65 μM, respectively). Furthermore, among the alterporriol-type dimers, alterporriol P (**207**) (Figure 27) was found to possess cytotoxicity against PC-3 and HCT-116 cell lines, with IC_50_ values of 6.4 and 8.6 μM, respectively [172]. 

From the fungus *Chondrostereum* sp., which was isolated from the soft coral *Sarcophyton tortuosum,* Li et al. [173] afforded five new triquinane-type sesquiterpenoids, chondrosterins A–E, and a known sesquiterpenoid, hirsutanol C. Among them, chondrosterin A (**208**) (Figure 27) showed significant cytotoxic activities against cancer lines A549, CNE2, and LoVo, with IC_50_ values of 2.45, 4.95, and 5.47 μM, respectively. In another study, two new chlorinated polyketides, strepchloritides A and B; three thiazole derivatives, watasemycin A, pulicatin G, and aerugine; and pyrrole-2-carboxamide, furan-2-carboxamide, and 1-(3,5-dihydroxyphenyl)ethanone were isolated from the soft coral-associated actinomycetes strain *Streptomyces* sp. OUCMDZ-1703. Interestingly, the new compounds strepchloritide A (**209**) (Figure 27) and strepchloritide B displayed cytotoxicity against the MCF-7 cells, with IC_50_ values of 9.9 and 20.2 μM, respectively [174]. Chemical investigation of the coral-derived fungus *Aspergillus tritici* SP2-8-1 obtained three novel compounds, 4-methyl-candidusin A, aspetritone A, and aspetritone B, together with 15 known compounds. Among them, compounds aspetritone A (**210**) and 3-prenylterphenyllin (**211**) (Figure 27) exhibited potent cytotoxic activities against human cancer cell lines HeLa, A549, and HepG2, with IC_50_ values from 2.10 ± 0.20 to 3.87 ± 0.74 µM, whereas aspetritone B (**212**) (Figure 27) also showed significant cytotoxicity toward cancer cell lines A549 and HepG2, with IC_50_ values of 4.67 ± 0.60 and 8.57 ± 0.83 µM, respectively. It was noted that the cytotoxicity of **210** and **212** against the Hep G2 cells was comparable to that of the positive control doxorubicin. Preliminary structure–activity relationship studies implied that the prenylation of terphenyllin or candidusin and the tetrahydrobenzene moiety in anthraquinone derivatives may be important for their bioactivity [175].

### 3.2. Antimicrobial Compounds from Coral-Associated Microorganisms

**Figure 28:**
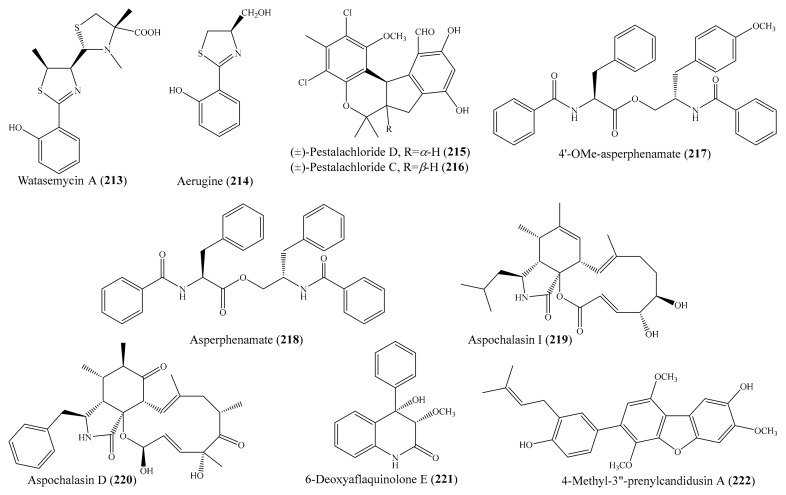
Chemical structures of **213**–**222**.

Many compounds from coral-associated microorganisms have been found to exhibit antimicrobial activity (Supplementary Table S8). Several of them possess potent activity and present as potential antimicrobial drugs. From a coral-associated actinomycetes strain, *Streptomyces* sp. OUCMDZ-1703, two new chlorinated polyketides and three thiazole derivatives, along with pyrrole-2-carboxamide, furan-2-carboxamide, and 1-(3,5-dihydroxyphenyl)ethanone, were isolated. Notably, compounds watasemycin A (**213**) and aerugine (**214**) (Figure 28) were first found to exhibit strong antimicrobial activity against *S. aureus* (ATCC25923) and three clinical strains of methicillin-resistant *Staphylococcus aureus* (MRSA082, MRSA111, and MRSA234), with MIC values of 1.95–7.81 μg/mL (5.5–37.4 µM) [174]. In another study, a new chlorinated benzophenone derivative, (±)-pestalachloride D (**215**), along with a related analogue, (±)-pestalachloride C (**216**) (Figure 28), were isolated from the fungus *Pestalotiopsis* sp., which is associated with the soft coral *Sarcophyton* sp. Bioassay results indicated that both compounds **215** and **216** exhibited antibacterial activity against *Escherichia coli*, *Vibrio anguillarum*, and *Vibrio parahaemolyticus*, with MIC values of 5.0, 10.0, and 20.0 μM, respectively [176]. Zheng et al. [177] reported the isolation of one new phenylalanine derivative, 4′-OMe-asperphenamate, along with one known phenylalanine derivative, asperphenamate, and two new cytochalasins, aspochalasin A1 and cytochalasin Z24, as well as eight known cytochalasin analogues from *Aspergillus elegans* ZJ-2008010, a fungus obtained from soft coral *Sarcophyton* sp. Compounds 4′-OMe-asperphenamate (**217**) and asperphenamate (**218**) (Figure 28) showed selective antibacterial activity against *S. epidermidis*, with MIC values of 10 μM for each, whereas compound aspochalasin I (**219**) (Figure 28) showed antibacterial activity against *S. epidermidis* and *S. aureus*, with MIC values of 20 and 10 μM, respectively: aspochalasin D (**220**) (Figure 28) showed antibacterial activity toward a broad spectrum of pathogenic bacteria (*S. epidermidis*, *S. aureus*, *E. coli*, and *B. cereus*), with MIC values of 10 μM. Chemical investigation of the coral-derived fungus *Scopulariopsis* sp. afforded six known dihydroquinolin-2-one-containing alkaloids. Notably, compound 6-deoxyaflaquinolone E (**221**) (Figure 28) exhibited extensive antibacterial activity against the tested bacteria, especially *S. aureus, B. cereus, V. parahaemolyticus, N. brasiliensis*, and *P. putida*, with MIC values of 0.78, 1.56, 6.25, 0.78, and 1.56 μM, respectively [178]. The antimicrobial activity of **221** against *S. aureus* and *N. brasiliensis* was comparable to that of the positive control ciprofloxacin, with MIC values of 0.625 and 0.625 μM, respectively. In 2017, Wang et al. [175] isolated three novel compounds together with 15 known compounds from a coral-derived microorganism, *Aspergillus tritici* SP2-8-1. Of these, the compounds aspetritone A (**210**) (Figure 27) and 4-methyl-3″-prenylcandidusin A (**222**) (Figure 28) exhibited better activities against the methicillin-resistant strains of *S. aureus* (MRSA) ATCC 43300 and MRSA CGMCC 1.12409 than the positive control chloramphenicol did. Compound **222** displayed stronger anti-MRSA than **210** and showed stronger antibacterial activities against strains of *Vibrio vulnificus, Vibrio rotiferianus,* and *Vibrio campbellii* than the other compounds did. From *Alternaria* sp. ZJ-2008003, a fungus obtained from soft coral *Sarcophyton* sp., three antimicrobial compounds (**206** (Figure 27), macrosporin, and alterporriol C) were isolated (Section 3.1). Interestingly, the antimicrobial activity of compound **206** against *E. coli* was comparable to that of the positive control ciprofloxacin (MIC = 0.62 μM) [172].

### 3.3. Antivirus Compounds from Coral-Associated Microorganisms

In addition to antimicrobial activity, several antivirus metabolites have been isolated from coral-derived microorganisms (Supplementary Table S9); however, these compounds exhibited moderate activity. For example, from *Alternaria* sp. ZJ-2008003, obtained from the soft coral *Sarcophyton* sp., five new hydroanthraquinone derivatives and five new alterporriol-type anthranoid dimers, along with seven known analogues, were isolated. Among them, only three compounds (tetrahydroaltersolanol C, alterporriol Q, and alterporriol C) exhibited antiviral activity against the porcine reproductive and respiratory syndrome virus (PRRSV), with IC_50_ values of 65, 22, and 39 μM, respectively [172]. In another study, Jia et al. [179] isolated a new phthalide derivative, pestalotiolide A, along with three known analogues and 5′-O-acetyl uridine from fungus *Pestalotiopsis* sp. ZJ-2009-7-6 isolated from a soft coral *Sarcophyton* sp. Compared to the positive control ribavirin (IC_50_ = 418.0 µM), pestalotiolide A exhibited anti-EV71 activity, with an IC_50_ value of 27.7 μM, whereas compound 7-hydroxy-5-methoxy-4,6-dimethyl-7-*O*-β-D-glucopyranosyl-phthalide showed strong antiviral activities against EV71, RSV, and HSV-1, with IC_50_ values of 51.6, 25.6, and 63.9 μM, respectively. Furthermore, compound 7-hydroxy-5-methoxy-4,6-dimethylphthalide displayed pronounced antiviral activities against Cox-B3 and RSV, with IC_50_ values of 19.6 μM and 21.0 μM, which were stronger than those of the positive control ribavirin, with IC_50_ values of 39.0 and 78.0 μM, respectively. Compound 5′-*O*-acetyl uridine and 7-hydroxy-5-methoxy-4,6-dimethyl-7-*O*-*α*-L-rhamnosyl-phthalide shared similar antiviral activities, with pronounced antiviral activities against EV71. Analyses of the structure–activity relationship of the phthalide derivatives revealed that the glycosidation of 7-OH significantly increased anti-EV71 activity and that the acetylation of 6′-OH increased anti-EV71 activity. Furthermore, the acetoxy group at C-6′ had a positive contribution to anti-EV71 activity.

### 3.4. Antifouling Compounds from Coral-Associated Microorganisms

**Figure 29:**
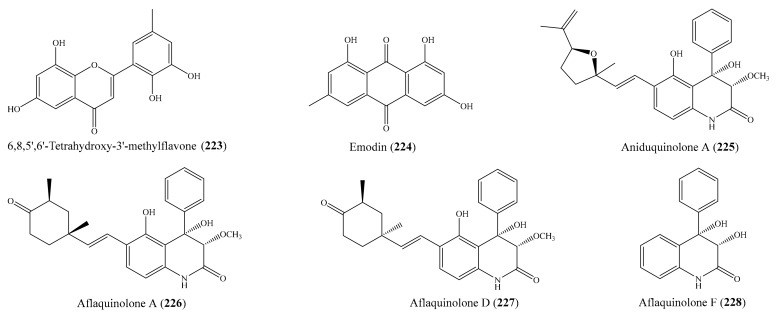
Chemical structures of **223**–**228**.

Several antifouling secondary metabolites have been isolated and identified from coral-derived microorganisms (Supplementary Table S10), including some potent active compounds. From *Aspergillus elegans* ZJ-2008010, a fungus obtained from soft coral *Sarcophyton* sp., Zheng et al. [177] isolated one new phenylalanine derivative, one known phenylalanine derivative, two new cytochalasins, as well as eight known cytochalasin analogues. Among them, aspochalasin D (**220**) (Figure 28) and H–J showed antifouling activity against a larval settlement of the barnacle *Balanus amphitrite*, with EC_50_ values ranging from 6.2 to 37 μM. Compound **220**, bearing an α,β-unsaturated ketone moiety, displayed considerably stronger activity than aspochalasin J, with an α,β-unsaturated lactone moiety, implying the importance of an electrophilic α,β-unsaturated carbonyl moiety for the antifouling activity of these cytochalasins. In addition, compound **220** exhibited more activity than aspochalasin H, suggesting that the double bond at C-19 and C-20 may be important in the antifouling activity of cytochalasins. A chemical investigation of the coral-associated fungus *Penicillium* sp. SCSGAF 0023, isolated from the coral *Dichotella gemmacea*, led to the isolation of two new polyketides, together with six known analogues. Notably, compounds 6,8,5′6′-tetrahydroxy-3′-methylflavone (**223**), emodin (**224**) (Figure 29), citreorosein, and isorhodoptilometrin had significant antifouling activity against a *Balanus amphitrite* larvae settlement, with EC_50_ values of 6.7, 6.1, 17.9, and 13.7 µg/ml (22.3, 22.6, 62.5, and 43.6 µM), respectively. Besides, compounds **223** and **224** had low cytotoxicity, with an LC_50_/EC_50_ ratio >14.9 and >16.5, respectively [180]. It is noted that the standard requirement of an efficacious EC_50_ level for a natural antifoulant is 25 µg/ml, which was established by the U.S. Navy program [161], and a compound with an LC_50_/EC_50_ ratio >15 is often considered to be a nontoxic antifouling compound [157]. The results indicated that **222** and **223** are promising natural antifoulants. In another study, Shao et al. [178] isolated six known dihydroquinolin-2-one-containing alkaloids from fungus *Scopulariopsis* sp. obtained from the coral *Carijoa* sp. Interestingly, five out of the six isolated compounds, aniduquinolone A (**225**), aflaquinolone A (**226**) (Figure 29), aflaquinolone D (**227**), 6-deoxyaflaquinolone E (**221**) (Figure 28), and aflaquinolone F (**228**) (Figure 29), showed potent antifouling activity against a larval settlement of the barnacle *Balanus amphitrite*, with IC_50_ values from 17.5 pM to 1.04 μM. Compounds **225** and **226** were found to be the most promising nontoxic antilarval settlement candidates. In particular, compound **225** has been the strongest antifouling compound in nature up to now, showing highly potent activity at a picomolar level (EC_50_ = 17.5 pM) and a very safe and high therapeutic ratio (LC_50_/EC_50_ = 1200). Analyses of the structure–activity relationship of **225**–**227** indicated that the tetrahydrofuran ring at C-18 was more important for antifouling activity than the tetrahydrofuran ring at C-18. It was found that compounds **221** and **228**, with α configurations of OH or OCH_3_ at C-3, exhibited 100-fold greater efficacy than aflaquinolone G, with a β configuration of OH at C-3, thus suggesting that the configuration at C-3 played an apparent role in the antifouling activity. However, there was no appreciable change in the antifouling efficacy between **221** and **228**, implying that the methylation of OH at C-3 in **228** and **221** had very little effect on antifouling activity.

### 3.5. Other Bioactive Compounds from Coral-Associated Microorganisms

**Figure 30:**
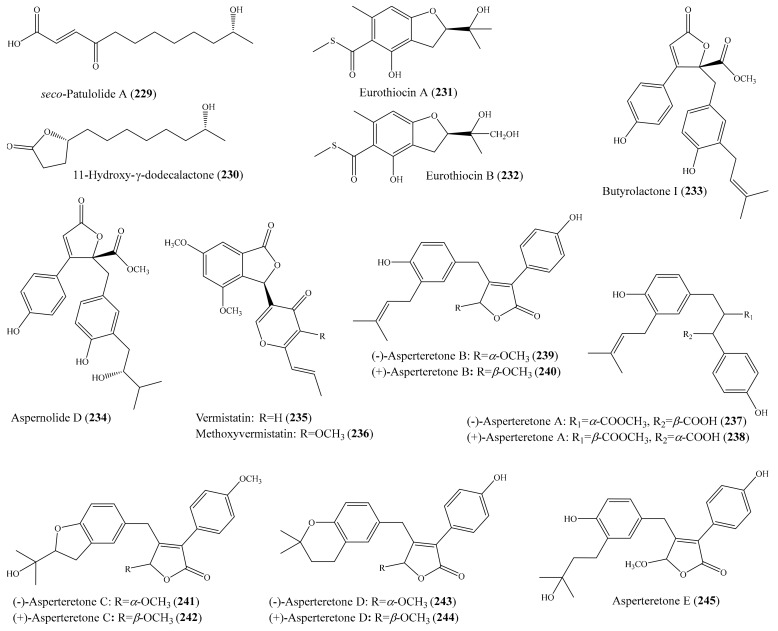
Chemical structures of **229**–**245**.

Apart from anti-inflammatory, cytotoxic, antimicrobial, antivirus, and antifouling activities, coral-derived microorganisms could produce protease inhibitory, lipid-lowering, α-glucosidase, and antioxidant compounds (Supplementary Table S11). For example, from the fermentation broth of the coral-associated fungus *Aspergillus versicolor* LCJ-5-4, which was isolated from soft coral *Cladiella* sp., three new cyclopentapeptides, versicoloritides A–C; a new orcinol tetramer, tetraorcinol A; two new lactones, versicolactones A and B; and three known metabolites were isolated. Among them, the compound tetraorcinol A showed moderate radical scavenging activity against the DPPH radical, with an IC_50_ value of 67 µM [181]. In another study, Hawasa et al. [182] isolated five metabolites from the ethyl acetate extract of the coral-associated fungus *Alternaria alternate*, which was isolated from the coral *Litophyton arboretum*. Among them, the compounds alternar-iol-9-methyl ether, alternariol, and alternariol-9-methyl ether-3-*O*-sulphate showed inhibitory activity against HCV NS3-NS4A, with IC_50_ values of 32.2, 12.0, and 52.0 μg/mL (118.3, 46.5, and 148 µM), respectively. A chemical investigation of the coral-derived fungus *Cladosporium* sp. TZP-29 led to the isolation of two new C12 polyketides, cladospolides E and F, together with four known derivatives. Interestingly, *seco*-patulolide A (**229**) and 11-hydroxy-*γ*-dodecalactone (**230**) (Figure 30) exhibited strong lipid-lowering activity in HepG2 hepatocytes, with IC_50_ values of 8.4 and 7.1 µM, respectively, while the compounds cladospolide E and *seco*-patulolide C showed less activity, with IC_50_ values of 12.1 and 13.1 µM, respectively. The lipid-lowering activity of **229** and **230** was comparable to that of the positive control lovastatin, with an IC_50_ value of 8.1 µM [183]. 

Besides, several *α*-glucosidase inhibitory compounds have been isolated from coral-derived microorganisms. Liu et al. [184] isolated two new sulfur-containing benzofuran derivatives, eurothiocins A and B, along with five known compounds, from cultures of *Eurotium rubrum* SH-823, a fungus obtained from soft coral *Sarcophyton* sp. Notably, six compounds (eurothiocins A (**231**) and B (**232**), butyrolactone I (**233**), aspernolide D (**234**), vermistatin (**235**), and methoxyvermistatin (**236**) (Figure 30)) exhibited more potent inhibitory effects against *α*-glucosidase activity than the clinical *α*-glucosidase inhibitor acarbose (IC_50_ = 376.7 ± 5.2 µM). In addition, a mechanistic analysis showed that **231** and **232** exhibited competitive inhibition characteristics [184]. In another study, Liu et al. [185] also isolated nine novel butenolide derivatives, including four pairs of enantiomers, (±)-asperteretones A–D (**237**–**244**), and a racemate, asperteretone E (**245**) (Figure 30), from the fungus *Aspergillus terreus*, which was isolated from the soft coral *Sarcophyton subviride*. Interestingly, all of these compounds exhibited potent inhibitory activity against *α*-glucosidase, with IC_50_ values ranging from 15.7 ± 1.1 to 53.1 ± 1.4 μM, which was much lower than those of the positive control acarbose (IC_50_ = 154.7 ± 8.1 μM), suggesting them as promising leading compounds for the discovery of new *α*-glucosidase inhibitors for type-2 diabetes mellitus treatment. Interestingly, it was observed that all enantiomers displayed nearly similar IC_50_ values against α-glucosidase inhibitory activity, suggesting that the difference of chirality may have a negligible influence on their activity.

## 4. Comprehensive Overview and Outlook

The present review revealed that coral and its associated microorganisms are a prolific source of structurally diverse secondary metabolites with different biological activities (Supplementary Tables S1–S11). Particularly, our review highlighted 245 highly bioactive compounds as a promising source for therapeutic agents. These compounds possess a wide range of bioactivities, such as anti-inflammatory, cytotoxic, antimicrobial, antivirus, and anti-fouling activities, among others. Beyond the current in vitro bioassays, further in vivo and preclinical and clinical studies of these bioactive compounds are required to determine their potential therapeutic applications.

**Figure 31:**
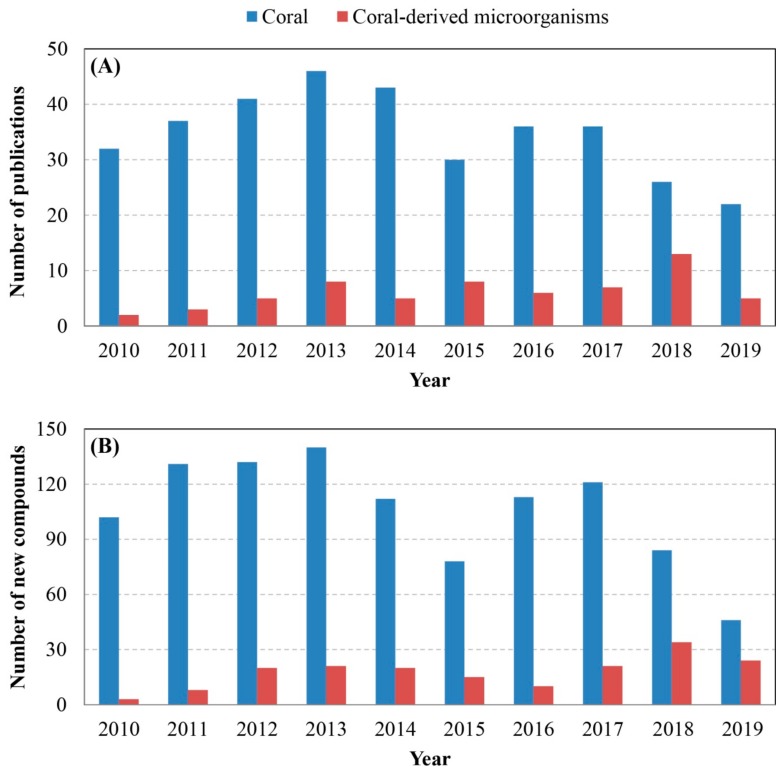
Number of PubMed publications describing natural products from coral and coral-derived microorganisms (**A**); number of new compounds produced by coral and coral-derived microorganisms (**B**) covering 2010–March 2019.

In general, there has been a slight decrease in the number of publications and the number of new compounds from coral over the last decade, whereas the number of publications and new compounds from coral-derived microorganisms slightly increased over the same period (Figure 31). Although coral, as well as other marine invertebrate organisms, can produce rich sources of bioactive natural products with a wide range of bioactivities, their pharmaceutical potentials are often challenged by a supply problem [11,186,187,188]. Marine organisms often produce minute quantities of natural products: therefore, the collection of large amounts of marine organisms for drug discovery and development is not a sustainable strategy. In other words, microorganism-associated hosts (e.g., sponge, coral) may also produce diverse natural products and offer potential for the exploitation of bioactive metabolites [189,190,191,192]. Hence, the discovery of novel bioactive natural products for drug development has been gradually shifted to host-associated microbes.

**Figure 32:**
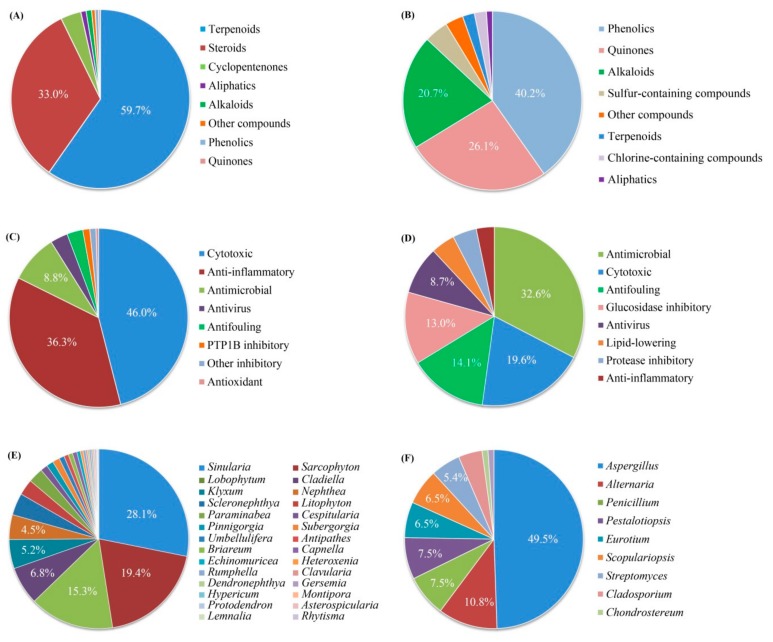
Distribution of the bioactive compounds according to chemical structure for coral (**A**) and coral-derived microorganisms (**B**); according to activity type in coral (**C**) and coral-derived microorganisms (**D**); according to genera of coral (**E**) and coral-derived microorganisms (**F**).

According to structural type, bioactive natural products produced by coral are dominated by terpenoids (59.7%) and steroids (33.0%), followed by cyclopentenones, aliphatics, alkaloids, phenolics, quinones, and other compounds (Figure 32A), whereas bioactive compounds produced by coral-derived microorganisms are dominated by three main classes: phenolics (40.2%), quinones (26.1%), and alkaloids (20.7%). Other compounds account for less than 5% for each (Figure 32B). Based on dominating structure classes, the structure–activity relationship should be analyzed to facilitate the design and synthesis of interesting compounds and their analogues.

As seen in Figure 32C, the biological activities of compounds from coral are mainly focused in the areas of cytotoxic (46.0%), anti-inflammatory (36.3%), and antimicrobial activity (8.8%); whereas other selective activities, including antivirus, antifouling, antioxidant, PTP1B inhibitory, and other inhibitory activities, account for less than 5% for each. For coral-associated microorganisms, the bioactivity of compounds is mainly focused in the areas of antimicrobial (32.6%), cytotoxic (19.6%), antifouling (14.1%), and α-glucosidase inhibitory activity (13.0%). Furthermore, other activities such as antiviral, lipid-lowering, protease inhibitory, and anti-inflammatory activities are found in compounds produced by coral-associated microorganisms (Figure 32D).

It was observed that coral-derived bioactive compounds are found in nearly 30 genera. The majority of coral-derived bioactive compounds are produced by three main genera: *Simularia* (28.1%), *Sarcophyton* (19.4%), and *Lobophytum* (15.3%) (Figure 32E), suggesting that these genera should be subjected to advanced aquaculture studies in order to obtain enough biomass for further preclinical and clinical trials and commercialization. Several corals (e.g., *Sinularia* sp., *S. gaweli, S. flexilis*) have begun to be transplanted into culturing tanks with a flow-through seawater system located in the National Museum of Marine Biology and Aquarium, Taiwan, for the extraction of additional natural products in order to establish a stable supply of bioactive material [107,117,118]. For coral-associated microorganisms (Figure 32F), fungi and bacteria are two groups that have been found to produce bioactive compounds; however, the large majority of bioactive compounds are produced by fungi (93.5%), whereas bacteria account for only 6.5%. Particularly, approximately 50% of bioactive compounds are produced by the genus *Aspergillus*, implying that *Aspergillus* is one of the key producers of natural products from coral-derived microorganisms. Further studies on the chemical and biological activities of this genus should be investigated. Specifically, advanced approaches that allow for the activation of the expression and production of novel/cryptic compounds of *Aspergillus* should be developed.

Although bioactive compounds from coral and its associated microorganisms show interesting levels for a number of clinically relevant targets, they are not well represented in the pipelines of drugs and none of them has been successfully marketed as a pharmaceutical. While the exploitation of bioactive natural products from coral for drug development is still limited by a supply problem and the sustainable conservation of coral reefs [11,186,187,188], drug discovery from coral-derived microorganisms is more sustainable and can overcome the supply issue through the large-scale fermentation of microorganisms. However, drug development from coral-derived microorganisms has been hampered due to only a minor number of microbes being able to be maintained in laboratory conditions [24,193,194]. Furthermore, most of the biosynthetic gene clusters expressing novel bioactive metabolites remain silent or cryptic under laboratory culture conditions [195,196,197]. Therefore, in order to unravel and exploit novel bioactive compounds from coral for drug development, the development of advanced aquacultures is required to obtain enough biomass for preclinical and clinical trials and commercialization [198,199,200,201]. For coral-associated microorganisms, new methods and technologies are warranted to activate the expression of the novel/cryptic secondary metabolome of microbes. In addition, new cultivation methods enhancing the cultivation of novel microbes and providing suitable culture conditions for the activation of silent genes of novel/cryptic biosynthetic gene clusters should be developed (e.g., cocultivation, in situ culture or culture stimulated in natural conditions, and the use of “omics”-based culture media). In particular, with the development of next-generation sequencing technologies, cultivation-independent methods (e.g., genomics, metagenomics, proteomics) should be applied to screen and identify gene clusters involved in the biosynthesis of promising secondary metabolites from yet uncultivable coral-derived microbes. Then, the expression of the biosynthetic gene clusters of interesting metabolites through appropriate heterologous expression systems can be required to produce the metabolites [202]. Combination of “omics”-based approaches and molecular approaches are expected to open up an unexplored reservoir of bioactive natural products from uncultivable coral-derived microbes.

It is noted that the supply problem of natural products from coral and coral-derived microorganisms can also be overcome by synthesis and/or semisynthesis approaches. The present review found that several compounds from coral and coral-derived microorganisms exhibited very strong activity (IC_50_ < 1 µM), stronger activity than the positive controls (e.g., **97**–**99**, **114**–**117**, **179**–**182**, **221**), indicating they are potential lead compounds. Therefore, obtaining such compounds and derivatives in sufficient amounts for further studies of their biological properties is of interest. Previous studies have revealed that several potential compounds and their analogues from coral have been synthesized successfully (e.g., acerosolide, bipinnatin J, rubifolide, deoxypukalide, pseudopterosins, fuscosides) [203,204,205,206,207,208,209]. However, a number of synthesized metabolites remain modest compared to discovered natural products, and the synthetic yield is low. Despite promising developments, many limitations should be considered in synthesis studies. The complex structural features of natural products often require an enormous number of tedious steps, resulting in low yields and small quantities of the target compounds. Furthermore, due to the large number of tedious steps that are required, the amount of waste products (e.g., toxic solvents, expensive and corrosive reagents) increases. Hence, the development and application of advanced synthesis strategies is needed to enhance the efficiency of syntheses of interesting compounds from coral and coral-derived microorganisms.

## 5. Conclusions

This review elucidated the structure and bioactivity of 245 potential compounds from coral and coral-derived microorganisms as promising candidates for further clinical trials. These compounds possess diverse structures and bioactivities. In particular, some compounds have exhibited very strong activity, with IC_50_ < 1 µM and stronger activity than the positive controls, suggesting that these compounds could be used as potential lead compounds in the synthesis of more active analogues and/or new drugs. It is noted that the development of new drugs from coral and coral-derived microorganisms has been hampered by a supply problem. Thus, efforts to introduce and develop new approaches as well as improve current approaches, including synthesis, aquaculture, cultivation, and molecular approaches, are expected to unravel hidden potential natural products of coral and coral-derived microorganisms.

## Supplementary Materials

The following are available online at https://www.mdpi.com/1660-3397/17/8/468/s1: Supplementary.docx. Tables S1–S11: Bioactive compounds from coral and its associated microorganisms.

## References

[1] Pietra F. (2002). Biodiversity and Natural Product Diversity.

[2] Hay M.E., Fenical W. (1996). Chemical ecology and marine biodiversity: Insights and products from the sea. Oceanography.

[3] Puglisi M.P., Sneed J.M., Ritson-Williams R., Young R. (2019). Marine chemical ecology in benthic environments. Nat. Prod. Rep..

[4] Rohde S., Nietzer S., Schupp P.J. (2015). Prevalence and Mechanisms of Dynamic Chemical Defenses in Tropical Sponges. PLoS ONE.

[5] Coll J.C., Barre S.L., Sammarco P.W., Williams W.T., Bakus G.J. (1982). Chemical Defences in Soft Corals (Coelenterata: Octocorallia) of the Great Barrier Reef: A Study of Comparative Toxicities. Mar. Ecol. Prog. Ser..

[6] McClintock J.B., Amsler C.D., Baker B.J. (2010). Overview of the Chemical Ecology of Benthic Marine Invertebrates along the Western Antarctic Peninsula. Integr. Comp. Biol..

[7] Lindquist N. (2002). Chemical Defense of Early Life Stages of Benthic Marine Invertebrates. J. Chem. Ecol..

[8] Eskander R., Al-Sofyani A.A., El-Sherbiny M.M.O., Ba-Akdah M.A., Satheesh S. (2018). Chemical Defense of Soft Coral *Sinularia polydactyla* from the Red Sea Against Marine Biofilm-Forming Bacteria. J. Ocean Univ. China.

[9] Marris E. (2006). Drugs from the deep. Nature.

[10] Molinski T.F., Dalisay D.S., Lievens S.L., Saludes J.P. (2008). Drug development from marine natural products. Nat. Rev. Drug Discov..

[11] Lindequist U. (2016). Marine-Derived Pharmaceuticals—Challenges and Opportunities. Biomol. Ther..

[12] Malve H. (2016). Exploring the ocean for new drug developments: Marine pharmacology. J. Pharm. Bioallied Sci..

[13] De Zoysa M. (2012). Chapter 9—Medicinal Benefits of Marine Invertebrates: Sources for Discovering Natural Drug Candidates. Adv. Food Nutr. Res..

[14] Hu G.-P., Yuan J., Sun L., She Z.-G., Wu J.-H., Lan X.-J., Zhu X., Lin Y.-C., Chen S.-P. (2011). Statistical research on marine natural products based on data obtained between 1985 and 2008. Mar. Drugs.

[15] Horta A., Alves C., Pinteus S., Pedrosa R. (2015). The marine origin of drugs. Phycotoxins.

[16] Simmons T.L., Andrianasolo E., McPhail K., Flatt P., Gerwick W.H. (2005). Marine natural products as anticancer drugs. Mol. Cancer Ther..

[17] Blunt J.W., Copp B.R., Keyzers R.A., Munro M.H.G., Prinsep M.R. (2013). Marine natural products. Nat. Prod. Rep..

[18] Blunt J.W., Copp B.R., Keyzers R.A., Munro M.H.G., Prinsep M.R. (2012). Marine natural products. Nat. Prod. Rep..

[19] Blunt J.W., Copp B.R., Munro M.H.G., Northcote P.T., Prinsep M.R. (2011). Marine natural products. Nat. Prod. Rep..

[20] Wu Q., Sun J., Chen J., Zhang H., Guo Y.-W., Wang H. (2018). Terpenoids from Marine Soft Coral of the Genus *Lemnalia*: Chemistry and Biological Activities. Mar. Drugs.

[21] Rodrigues G.I., Miguel G.M., Mnif W. (2019). A Brief Review on New Naturally Occurring Cembranoid Diterpene Derivatives from the Soft Corals of the Genera *Sarcophyton*, *Sinularia*, and *Lobophytum* Since 2016. Molecules.

[22] Mayer A.M.S., Rodríguez A.D., Berlinck R.G.S., Fusetani N. (2011). Marine pharmacology in 2007–8: Marine compounds with antibacterial, anticoagulant, antifungal, anti-inflammatory, antimalarial, antiprotozoal, antituberculosis, and antiviral activities; affecting the immune and nervous system, and other miscellaneous mechanisms of action. Comp. Biochem. Physiol. C Toxicol. Pharmacol..

[23] Kooperman N., Ben-Dov E., Kramarsky-Winter E., Barak Z., Kushmaro A. (2007). Coral mucus-associated bacterial communities from natural and aquarium environments. FEMS Microbiol. Lett..

[24] Rohwer F., Breitbart M., Jara J., Azam F., Knowlton N. (2001). Diversity of bacteria associated with the Caribbean coral *Montastraea franksi*. Coral Reefs.

[25] Pollock F.J., McMinds R., Smith S., Bourne D.G., Willis B.L., Medina M., Thurber R.V., Zaneveld J.R. (2018). Coral-associated bacteria demonstrate phylosymbiosis and cophylogeny. Nat. Commun..

[26] Huggett M.J., Apprill A. (2019). Coral microbiome database: Integration of sequences reveals high diversity and relatedness of coral-associated microbes. Environ. Microbiol. Rep..

[27] Mahmoud H.M., Kalendar A.A. (2016). Coral-associated actinobacteria: Diversity, abundance, and biotechnological potentials. Front. Microbiol..

[28] Lawler S.N., Kellogg C.A., France S.C., Clostio R.W., Brooke S.D., Ross S.W. (2016). Coral-Associated Bacterial Diversity Is Conserved across Two Deep-Sea Anthothela Species. Front. Microbiol..

[29] La Rivière M., Garrabou J., Bally M. (2015). Evidence for host specificity among dominant bacterial symbionts in temperate gorgonian corals. Coral Reefs.

[30] van de Water J.A.J.M., Melkonian R., Voolstra C.R., Junca H., Beraud E., Allemand D., Ferrier-Pagès C. (2017). Comparative Assessment of Mediterranean Gorgonian-Associated Microbial Communities Reveals Conserved Core and Locally Variant Bacteria. Microb. Ecol..

[31] Rosenberg E., Koren O., Reshef L., Efrony R., Zilber-Rosenberg I. (2007). The role of microorganisms in coral health, disease and evolution. Nat. Rev. Microbiol..

[32] Zaneveld J.R., Burkepile D.E., Shantz A.A., Pritchard C.E., McMinds R., Payet J.P., Welsh R., Correa A.M.S., Lemoine N.P., Rosales S. (2016). Overfishing and nutrient pollution interact with temperature to disrupt coral reefs down to microbial scales. Nat. Commun..

[33] Sunagawa S., DeSantis T.Z., Piceno Y.M., Brodie E.L., DeSalvo M.K., Voolstra C.R., Weil E., Andersen G.L., Medina M. (2009). Bacterial diversity and White Plague Disease-associated community changes in the Caribbean coral *Montastraea faveolata*. ISME J..

[34] Sato Y., Willis B.L., Bourne D.G. (2009). Successional changes in bacterial communities during the development of black band disease on the reef coral, *Montipora hispida*. ISME J..

[35] Morrow K.M., Muller E., Lesser M.P., van Oppen M.J.H., Lough J.M. (2018). How Does the Coral Microbiome Cause, Respond to, or Modulate the Bleaching Process?. Coral Bleaching: Patterns, Processes, Causes and Consequences.

[36] Roitman S., Joseph Pollock F., Medina M. (2018). Coral Microbiomes as Bioindicators of Reef Health. Population Genomics.

[37] Alagely A., Krediet C.J., Ritchie K.B., Teplitski M. (2011). Signaling-mediated cross-talk modulates swarming and biofilm formation in a coral pathogen *Serratia marcescens*. ISME J..

[38] Kim B.R. (2006). Regulation of microbial populations by coral surface mucus and mucus-associated bacteria. Mar. Ecol. Prog. Ser..

[39] Teplitski M., Ritchie K. (2009). How feasible is the biological control of coral diseases?. Trends Ecol. Evol..

[40] Shnit-Orland M., Kushmaro A. (2009). Coral mucus-associated bacteria: A possible first line of defense. FEMS Microbiol. Ecol..

[41] Debbab A., Aly A.H., Lin W.H., Proksch P. (2010). Bioactive Compounds from Marine Bacteria and Fungi. Microb. Biotechnol..

[42] Lei H. (2016). Diterpenoids of Gorgonian Corals: Chemistry and Bioactivity. Chem. Biodivers..

[43] Su Y.-D., Su J.-H., Hwang T.-L., Wen Z.-H., Sheu J.-H., Wu Y.-C., Sung P.-J. (2017). Briarane Diterpenoids Isolated from Octocorals between 2014 and 2016. Mar. Drugs.

[44] Sheu J.-H., Chen Y.-H., Chen Y.-H., Su Y.-D., Chang Y.-C., Su J.-H., Weng C.-F., Lee C.-H., Fang L.-S., Wang W.-H. (2014). Briarane diterpenoids isolated from gorgonian corals between 2011 and 2013. Mar. Drugs.

[45] Chang Y.-C., Sheu J.-H., Wu Y.-C., Sung P.-J. (2017). Terpenoids from Octocorals of the Genus *Pachyclavularia*. Mar. Drugs.

[46] Carroll A.R., Copp B.R., Davis R.A., Keyzers R.A., Prinsep M.R. (2019). Marine natural products. Nat. Prod. Rep..

[47] Scrivo R., Vasile M., Bartosiewicz I., Valesini G. (2011). Inflammation as “common soil” of the multifactorial diseases. Autoimmun. Rev..

[48] Sostres C., Gargallo C.J., Arroyo M.T., Lanas A. (2010). Adverse effects of non-steroidal anti-inflammatory drugs (NSAIDs, aspirin and coxibs) on upper gastrointestinal tract. Best Pract. Res. Clin. Gastroenterol..

[49] Yan P., Lv Y., van Ofwegen L., Proksch P., Lin W. (2010). Lobophytones A−G, new isobiscembranoids from the soft coral *Lobophytum pauciflorum*. Org. Lett..

[50] Chen B.W., Chang S.M., Huang C.Y., Chao C.H., Su J.H., Wen Z.H., Hsu C.H., Dai C.F., Wu Y.C., Sheu J.H. (2010). Hirsutalins A-H, eunicellin-based diterpenoids from the soft coral *Cladiella hirsuta*. J. Nat. Prod..

[51] Quang T.H., Ha T.T., Minh C.V., Kiem P.V., Huong H.T., Ngan N.T., Nhiem N.X., Tung N.H., Tai B.H., Thuy D.T. (2011). Cytotoxic and anti-inflammatory cembranoids from the Vietnamese soft coral *Lobophytum laevigatum*. Bioorg. Med. Chem..

[52] Su J.H., Wen Z.H. (2011). Bioactive cembrane-based diterpenoids from the soft coral *Sinularia Triangular*. Mar. Drugs.

[53] Kao C.Y., Su J.H., Lu M.C., Hwang T.L., Wang W.H., Chen J.J., Sheu J.H., Kuo Y.H., Weng C.F., Fang L.S. (2011). Lobocrassins A-E: New cembrane-type diterpenoids from the soft coral *Lobophytum crassum*. Mar. Drugs.

[54] Chao C.H., Chou K.J., Wen Z.H., Wang G.H., Wu Y.C., Dai C.F., Sheu J.H. (2011). Paraminabeolides A-F, cytotoxic and anti-inflammatory marine withanolides from the soft coral *Paraminabea acronocephala*. J. Nat. Prod..

[55] Hsu F.-J., Chen B.-W., Wen Z.-H., Huang C.-Y., Dai C.-F., Su J.-H., Wu Y.-C., Sheu J.-H. (2011). Klymollins A–H, bioactive eunicellin-based diterpenoids from the Formosan soft coral *Klyxum molle*. J. Nat. Prod..

[56] Lin W.Y., Lu Y., Su J.H., Wen Z.H., Dai C.F., Kuo Y.H., Sheu J.H. (2011). Bioactive cembranoids from the dongsha atoll soft coral *Sarcophyton crassocaule*. Mar. Drugs.

[57] Chung H.M., Hong P.H., Su J.H., Hwang T.L., Lu M.C., Fang L.S., Wu Y.C., Li J.J., Chen J.J., Wang W.H. (2012). Bioactive compounds from a gorgonian coral *Echinomuricea* sp. (Plexauridae). Mar. Drugs.

[58] Lin W.Y., Lu Y., Chen B.W., Huang C.Y., Su J.H., Wen Z.H., Dai C.F., Kuo Y.H., Sheu J.H. (2012). Sarcocrassocolides M-O, bioactive cembranoids from the Dongsha Atoll soft coral *Sarcophyton crassocaule*. Mar. Drugs.

[59] Fang H.-Y., Liaw C.-C., Chao C.-H., Wen Z.-H., Wu Y.-C., Hsu C.-H., Dai C.-F., Sheu J.-H. (2012). Bioactive pregnane-type steroids from the soft coral *Scleronephthya gracillimum*. Tetrahedron.

[60] Chen Y.-H., Hwang T.-L., Su Y.-D., Chang Y.-C., Chen Y.-H., Hong P.-H., Hu L.-C., Yen W.-H., Hsu H.-Y., Huang S.-J. (2012). New 6-hydroxyeunicellins from a soft coral *Cladiella* sp.. Chem. Pharm. Bull..

[61] Yang B., Zhou X., Huang H., Yang X.-W., Liu J., Lin X., Li X., Peng Y., Liu Y. (2012). New cembrane diterpenoids from a Hainan soft coral *Sinularia* sp.. Mar. Drugs.

[62] Tai C.J., Su J.H., Huang C.Y., Huang M.S., Wen Z.H., Dai C.F., Sheu J.H. (2013). Cytotoxic and anti-inflammatory eunicellin-based diterpenoids from the soft coral *Cladiella krempfi*. Mar. Drugs.

[63] Thao N.P., Nam N.H., Cuong N.X., Quang T.H., Tung P.T., Dat Le D., Chae D., Kim S., Koh Y.S., Kiem P.V. (2013). Anti-inflammatory norditerpenoids from the soft coral *Sinularia maxima*. Bioorg. Med. Chem. Lett..

[64] Nguyen P.T., Nguyen H.N., Nguyen X.C., Nguyen X.N., Pham T.T., Tran H.Q., Nguyen T.T.N., Phan V.K., Chau V.M., Kim Y.H. (2013). A new sterol from the soft coral *Lobophytum crassum*. Bull. Korean Chem. Soc..

[65] Yin J., Zhao M., Ma M., Xu Y., Xiang Z., Cai Y., Dong J., Lei X., Huang K., Yan P. (2013). New casbane diterpenoids from a South China Sea soft coral, *Sinularia* sp.. Mar. Drugs.

[66] Lin M.C., Chen B.W., Huang C.Y., Dai C.F., Hwang T.L., Sheu J.H. (2013). Eunicellin-based diterpenoids from the Formosan soft coral *Klyxum molle* with inhibitory activity on superoxide generation and elastase release by neutrophils. J. Nat. Prod..

[67] Chen B.-W., Wang S.-Y., Huang C.-Y., Chen S.-L., Wu Y.-C., Sheu J.-H. (2013). Hirsutalins I–M, eunicellin-based diterpenoids from the soft coral *Cladiella hirsuta*. Tetrahedron.

[68] Thao N.P., Luyen B.T., Ngan N.T., Song S.B., Cuong N.X., Nam N.H., Kiem P.V., Kim Y.H., Minh C.V. (2014). New anti-inflammatory cembranoid diterpenoids from the Vietnamese soft coral *Lobophytum crassum*. Bioorg. Med. Chem. Lett..

[69] Cuong N.X., Thao N.P., Luyen B.T.T., Ngan N.T.T., Thuy D.T.T., Song S.B., Nam N.H., Van Kiem P., Kim Y.H., Van Minh C. (2014). Cembranoid diterpenes from the soft coral *Lobophytum crassum* and their anti-inflammatory activities. Chem. Pharm. Bull..

[70] Thao N.P., Luyen B.T., Sun Y.N., Song S.B., Thanh N.V., Cuong N.X., Nam N.H., Kiem P.V., Kim Y.H., Minh C.V. (2014). NF-kappaB inhibitory activity of polyoxygenated steroids from the Vietnamese soft coral *Sarcophyton pauciplicatum*. Bioorg. Med. Chem. Lett..

[71] Lee Y.N., Tai C.J., Hwang T.L., Sheu J.H. (2014). Krempfielins N-P, new anti-inflammatory eunicellins from a Taiwanese soft coral *Cladiella krempfi*. Mar. Drugs.

[72] Lin W.Y., Chen B.W., Huang C.Y., Wen Z.H., Sung P.J., Su J.H., Dai C.F., Sheu J.H. (2014). Bioactive cembranoids, sarcocrassocolides P-R, from the Dongsha Atoll soft coral *Sarcophyton crassocaule*. Mar. Drugs.

[73] Chao C.-H., Huang T.-Z., Wu C.-Y., Chen B.-W., Huang C.-Y., Hwang T.-L., Dai C.-F., Sheu J.-H. (2015). Steroidal and α-tocopherylhydroquinone glycosides from two soft corals *Cladiella hirsuta* and *Sinularia nanolobata*. RSC Adv..

[74] Huang C.Y., Tseng Y.J., Chokkalingam U., Hwang T.L., Hsu C.H., Dai C.F., Sung P.J., Sheu J.H. (2016). Bioactive isoprenoid-derived natural products from a Dongsha Atoll soft coral *Sinularia erecta*. J. Nat. Prod..

[75] Tsai C.-R., Huang C.-Y., Chen B.-W., Tsai Y.-Y., Shih S.-P., Hwang T.-L., Dai C.-F., Wang S.-Y., Sheu J.-H. (2015). New bioactive steroids from the soft coral *Klyxum flaccidum*. RSC Adv..

[76] Huang C.Y., Sung P.J., Uvarani C., Su J.H., Lu M.C., Hwang T.L., Dai C.F., Wu S.L., Sheu J.H. (2015). Glaucumolides A and B, biscembranoids with new structural type from a cultured soft coral *Sarcophyton glaucum*. Sci. Rep..

[77] Tsai Y.Y., Huang C.Y., Tseng W.R., Chiang P.L., Hwang T.L., Su J.H., Sung P.J., Dai C.F., Sheu J.H. (2017). Klyflaccisteroids K-M, bioactive steroidal derivatives from a soft coral *Klyxum flaccidum*. Bioorg. Med. Chem. Lett..

[78] Chang Y.C., Kuo L.M., Hwang T.L., Yeh J., Wen J.H., Fang L.S., Wu Y.C., Lin C.S., Sheu J.H., Sung P.J. (2016). Pinnisterols A–C, new 9,11-secosterols from a Gorgonian *Pinnigorgia* sp.. Mar. Drugs.

[79] Huang C.Y., Chang C.W., Tseng Y.J., Lee J., Sung P.J., Su J.H., Hwang T.L., Dai C.F., Wang H.C., Sheu J.H. (2016). Bioactive steroids from the Formosan soft coral *Umbellulifera petasites*. Mar. Drugs.

[80] Tseng W.R., Huang C.Y., Tsai Y.Y., Lin Y.S., Hwang T.L., Su J.H., Sung P.J., Dai C.F., Sheu J.H. (2016). New cytotoxic and anti-inflammatory steroids from the soft coral *Klyxum flaccidum*. Bioorg. Med. Chem. Lett..

[81] Zhao M., Cheng S., Yuan W., Xi Y., Li X., Dong J., Huang K., Gustafson K.R., Yan P. (2016). Cembranoids from a Chinese collection of the soft coral *Lobophytum crassum*. Mar. Drugs.

[82] Chang Y.-C., Kuo L.-M., Su J.-H., Hwang T.-L., Kuo Y.-H., Lin C.-S., Wu Y.-C., Sheu J.-H., Sung P.-J. (2016). Pinnigorgiols A–C, 9,11-secosterols with a rare ring arrangement from a gorgonian coral *Pinnigorgia* sp.. Tetrahedron.

[83] Huang C.Y., Ahmed A.F., Su J.H., Sung P.J., Hwang T.L., Chiang P.L., Dai C.F., Liaw C.C., Sheu J.H. (2017). Bioactive new withanolides from the cultured soft coral *Sinularia brassica*. Bioorg. Med. Chem. Lett..

[84] Wu Q., Li X.W., Li H., Yao L.G., Tang W., Miao Z.H., Wang H., Guo Y.W. (2019). Bioactive polyoxygenated cembranoids from a novel Hainan chemotype of the soft coral *Sinularia flexibilis*. Bioorg. Med. Chem. Lett..

[85] Andrea N., Angelo C., Paola F., Pier Mario B., Giuseppe R. (2016). Immunotherapy and Hormone-therapy in Metastatic Breast Cancer: A Review and an Update. Curr. Drug Targets.

[86] Mullard A. (2015). FDA approves first immunotherapy combo. Nat. Rev. Drug Discov..

[87] Ali R., Mirza Z., Ashraf G.M.D., Kamal M.A., Ansari S.A., Damanhouri G.A., Abuzenadah A.M., Chaudhary A.G., Sheikh I.A. (2012). New Anticancer Agents: Recent Developments in Tumor Therapy. Anticancer Res..

[88] Khoo B.L., Chaudhuri P.K., Ramalingam N., Tan D.S.W., Lim C.T., Warkiani M.E. (2016). Single-cell profiling approaches to probing tumor heterogeneity. Int. J. Cancer.

[89] Feinberg A.P., Ohlsson R., Henikoff S. (2006). The epigenetic progenitor origin of human cancer. Nat. Rev. Genet..

[90] Nguyen H.T., Chau V.M., Phan V.K., Hoang T.H., Nguyen H.N., Nguyen X.C., Tran H.Q., Nguyen X.N., Hyun J.H., Kang H.K. (2010). Chemical components from the Vietnamese soft coral *Lobophytum* sp.. Arch. Pharm. Res..

[91] Chau V.M., Phan V.K., Nguyen X., Nguyen X.C., Nguyen P.T., Nguyen H.N., Hoang Le T.A., Do C.T., Thuy D.T., Kang H.K. (2011). Cytotoxic and antioxidant activities of diterpenes and sterols from the Vietnamese soft coral *Lobophytum compactum*. Bioorg. Med. Chem. Lett..

[92] Tai C.J., Su J.H., Huang M.S., Wen Z.H., Dai C.F., Sheu J.H. (2011). Bioactive eunicellin-based diterpenoids from the soft coral *Cladiella krempfi*. Mar. Drugs.

[93] Hegazy M.-E.F., El-Beih A.A., Moustafa A.Y., Hamdy A.A., Alhammady M.A., Selim R.M., Abdel-Rehim M., Paré P.W. (2011). Cytotoxic Cembranoids from the Red Sea Soft Coral *Sarcophyton glaucum*. Nat. Prod. Commun..

[94] Liao X.-J., Tang L.-D., Liang Y.-W., Geng H.-W., Xu S.-H. (2011). Isolation and identification of two new polyhydroxylated sterols from soft coral *Sinularia* sp.. Chem. Res. Chin. Univ..

[95] Lee N.L., Su J.H. (2011). Tetrahydrofuran cembranoids from the cultured soft coral *Lobophytum crassum*. Mar. Drugs.

[96] Cheng S.Y., Huang K.J., Wang S.K., Duh C.Y. (2011). Capilloquinol: A novel farnesyl quinol from the Dongsha atoll soft coral *Sinularia capillosa*. Mar. Drugs.

[97] Lin Y.S., Eid Fazary A., Chen C.H., Kuo Y.H., Shen Y.C. (2011). Bioactive xenicane diterpenoids from the Taiwanese soft coral *Asterospicularia laurae*. Chem. Biodivers..

[98] Huang C.Y., Su J.H., Duh C.Y., Chen B.W., Wen Z.H., Kuo Y.H., Sheu J.H. (2012). A new 9,11-secosterol from the soft coral *Sinularia granosa*. Bioorg. Med. Chem. Lett..

[99] Su J.H., Huang C.Y., Li P.J., Lu Y., Wen Z.H., Kao Y.H., Sheu J.H. (2012). Bioactive cadinane-type compounds from the soft coral *Sinularia scabra*. Arch. Pharm. Res..

[100] Hegazy M.E., Gamal Eldeen A.M., Shahat A.A., Abdel-Latif F.F., Mohamed T.A., Whittlesey B.R., Pare P.W. (2012). Bioactive hydroperoxyl cembranoids from the Red Sea soft coral *Sarcophyton glaucum*. Mar. Drugs.

[101] Roy P.K., Maarisit W., Roy M.C., Taira J., Ueda K. (2012). Five new diterpenoids from an Okinawan soft coral, *Cespitularia* sp.. Mar. Drugs.

[102] Li R., Shao C.L., Qi X., Li X.B., Li J., Sun L.L., Wang C.Y. (2012). Polyoxygenated sterols from the South China Sea soft coral *Sinularia* sp.. Mar. Drugs.

[103] Wang S.K., Duh C.Y. (2012). New cytotoxic cembranolides from the soft coral *Lobophytum michaelae*. Mar. Drugs.

[104] Wang S.K., Hsieh M.K., Duh C.Y. (2012). Three new cembranoids from the Taiwanese soft coral *Sarcophyton ehrenbergi*. Mar. Drugs.

[105] Wang S.K., Puu S.Y., Duh C.Y. (2012). New 19-oxygenated steroids from the soft coral *Nephthea chabrolii*. Mar. Drugs.

[106] Govindam S.V., Yoshioka Y., Kanamoto A., Fujiwara T., Okamoto T., Ojika M. (2012). Cyclolobatriene, a novel prenylated germacrene diterpene, from the soft coral *Lobophytum pauciflorum*. Bioorg. Med. Chem..

[107] Yen W.H., Hu L.C., Su J.H., Lu M.C., Twan W.H., Yang S.Y., Kuo Y.C., Weng C.F., Lee C.H., Kuo Y.H. (2012). Norcembranoidal diterpenes from a Formosan soft coral *Sinularia* sp.. Molecules.

[108] Murni A., Hanif N., Tanaka J. (2013). A new cytotoxic dolabellane from the Indonesian soft coral *Anthelia* sp.. Indones. J. Chem..

[109] Chao C.H., Wu Y.C., Wen Z.H., Sheu J.H. (2013). Steroidal carboxylic acids from soft coral *Paraminabea acronocephala*. Mar. Drugs.

[110] Su C.C., Wong B.S., Chin C., Wu Y.J., Su J.H. (2013). Oxygenated cembranoids from the soft coral *Sinularia flexibilis*. Int. J. Mol. Sci..

[111] Huang C.Y., Liaw C.C., Chen B.W., Chen P.C., Su J.H., Sung P.J., Dai C.F., Chiang M.Y., Sheu J.H. (2013). Withanolide-based steroids from the cultured soft coral *Sinularia brassica*. J. Nat. Prod..

[112] Aboutabl S.A., Azzam S.M., Michel C.G., Selim N.M., Hegazy M.F., Ali A.H., Hussein A.A. (2013). Bioactive terpenoids from the Red Sea soft coral *Sinularia polydactyla*. Nat. Prod. Res..

[113] Zhang J., Liao X.J., Wang K.L., Deng Z., Xu S.H. (2013). Cytotoxic cholesta-1,4-dien-3-one derivatives from soft coral *Nephthea* sp.. Steroids.

[114] Gong K.K., Tang X.L., Zhang G., Cheng C.L., Zhang X.W., Li P.L., Li G.Q. (2013). Polyhydroxylated steroids from the South China Sea soft coral *Sarcophyton* sp. and their cytotoxic and antiviral activities. Mar. Drugs.

[115] Zhao M., Yin J., Jiang W., Ma M., Lei X., Xiang Z., Dong J., Huang K., Yan P. (2013). Cytotoxic and antibacterial cembranoids from a South China Sea soft coral, *Lobophytum* sp.. Mar. Drugs.

[116] Ellithey M.S., Lall N., Hussein A.A., Meyer D. (2013). Cytotoxic, cytostatic and HIV-1 PR inhibitory activities of the soft coral *Litophyton arboreum*. Mar. Drugs.

[117] Yen W.H., Chen W.F., Cheng C.H., Dai C.F., Lu M.C., Su J.H., Su Y.D., Chen Y.H., Chang Y.C., Chen Y.H. (2013). A new 5α,8α-epidioxysterol from the soft coral *Sinularia gaweli*. Molecules.

[118] Kuo C.Y., Juan Y.S., Lu M.C., Chiang M.Y., Dai C.F., Wu Y.C., Sung P.J. (2014). Pregnane-type steroids from the Formosan soft coral *Scleronephthya flexilis*. Int. J. Mol. Sci..

[119] Elkhayat E.S., Ibrahim S.R.M., Fouad M.A., Mohamed G.A. (2014). Dendronephthols A–C, new sesquiterpenoids from the Red Sea soft coral *Dendronephthya* sp.. Tetrahedron.

[120] Chang F.Y., Hsu F.J., Tai C.J., Wei W.C., Yang N.S., Sheu J.H. (2014). Klymollins T-X, bioactive eunicellin-based diterpenoids from the soft coral *Klyxum molle*. Mar. Drugs.

[121] Al-Footy K.O., Alarif W.M., Asiri F., Aly M.M., Ayyad S.-E.N. (2014). Rare pyrane-based cembranoids from the Red Sea soft coral *Sarcophyton trocheliophorum* as potential antimicrobial–antitumor agents. Med. Chem. Res..

[122] Lei L.-F., Chen M.-F., Wang T., He X.-X., Liu B.-X., Deng Y., Chen X.-J., Li Y.-T., Guan S.-Y., Yao J.-H. (2014). Novel cytotoxic nine-membered macrocyclic polysulfur cembranoid lactones from the soft coral *Sinularia* sp.. Tetrahedron.

[123] Roy P.K., Roy M.C., Taira J., Ueda K. (2014). Structure and bioactivity of a trisnorditerpenoid and a diterpenoid from an Okinawan soft coral, *Cespitularia* sp.. Tetrahedron Lett..

[124] Cheng S.Y., Shih N.L., Hou K.Y., Ger M.J., Yang C.N., Wang S.K., Duh C.Y. (2014). Kelsoenethiol and dikelsoenyl ether, two unique kelsoane-type sesquiterpenes, from the Formosan soft coral *Nephthea erecta*. Bioorg. Med. Chem. Lett..

[125] Al-Lihaibi S.S., Alarif W.M., Abdel-Lateff A., Ayyad S.E., Abdel-Naim A.B., El-Senduny F.F., Badria F.A. (2014). Three new cembranoid-type diterpenes from Red Sea soft coral *Sarcophyton glaucum*: Isolation and antiproliferative activity against HepG2 cells. Eur. J. Med. Chem..

[126] Tseng Y.J., Yang Y.C., Wang S.K., Duh C.Y. (2014). Numerosol A-D, new cembranoid diterpenes from the soft coral *Sinularia numerosa*. Mar. Drugs.

[127] Lin Y.C., Wang S.S., Chen C.H., Kuo Y.H., Shen Y.C. (2014). Cespitulones A and B, cytotoxic diterpenoids of a new structure class from the soft coral *Cespitularia taeniata*. Mar. Drugs.

[128] Zhang N.X., Tang X.L., van Ofwegen L., Xue L., Song W.J., Li P.L., Li G.Q. (2015). Cyclopentenone derivatives and polyhydroxylated steroids from the soft coral *Sinularia acuta*. Chem. Biodivers..

[129] Nam N.H., Tung P.T., Ngoc N.T., Hanh T.T.H., Thao N.P., Thanh N.V., Cuong N.X., Thao D.T., Huong T.T., Thung D.C. (2015). Cytotoxic biscembranoids from the soft coral *Sarcophyton pauciplicatum*. Chem. Pharm. Bull..

[130] Cheng S.Y., Wang S.K., Hsieh M.K., Duh C.Y. (2015). Polyoxygenated cembrane diterpenoids from the soft coral *Sarcophyton Ehrenbergi*. Int. J. Mol. Sci..

[131] Hsiao T.H., Sung C.S., Lan Y.H., Wang Y.C., Lu M.C., Wen Z.H., Wu Y.C., Sung P.J. (2015). New anti-inflammatory cembranes from the cultured soft coral *Nephthea Columnaris*. Mar. Drugs.

[132] Koncic M.Z., Ioannou E., Sawadogo W.R., Abdel-Razik A.F., Vagias C., Diederich M., Roussis V. (2016). 4alpha-methylated steroids with cytotoxic activity from the soft coral *Litophyton mollis*. Steroids.

[133] Tsai T.C., Huang Y.T., Chou S.K., Shih M.C., Chiang C.Y., Su J.H. (2016). Cytotoxic oxygenated steroids from the soft coral *Nephthea erecta*. Chem. Pharm. Bull..

[134] Urda C., Fernandez R., Perez M., Rodriguez J., Jimenez C., Cuevas C. (2017). Protoxenicins A and B, cytotoxic long-chain acylated Xenicanes from the soft coral *Protodendron repens*. J. Nat. Prod..

[135] Chao C.H., Li W.L., Huang C.Y., Ahmed A.F., Dai C.F., Wu Y.C., Lu M.C., Liaw C.C., Sheu J.H. (2017). Isoprenoids from the soft coral *Sarcophyton glaucum*. Mar. Drugs.

[136] Mohammed R., Radwan M.M., Ma G., Mohamed T.A., Seliem M.A., Thabet M., ElSohly M.A. (2017). Bioactive sterols and sesquiterpenes from the Red Sea soft coral *Sinularia terspilli*. Med. Chem. Res..

[137] Wu C.H., Chao C.H., Huang T.Z., Huang C.Y., Hwang T.L., Dai C.F., Sheu J.H. (2018). Cembranoid-related metabolites and biological activities from the soft coral *Sinularia flexibilis*. Mar. Drugs.

[138] Livermore D.M. (2009). Has the era of untreatable infections arrived?. J. Antimicrob. Chemother..

[139] Roca I., Akova M., Baquero F., Carlet J., Cavaleri M., Coenen S., Cohen J., Findlay D., Gyssens I., Heuer O.E. (2015). The global threat of antimicrobial resistance: Science for intervention. New Microbes New Infect..

[140] Hughes D., Andersson D.I. (2017). Environmental and genetic modulation of the phenotypic expression of antibiotic resistance. FEMS Microbiol. Rev..

[141] Genilloud O. (2014). The re-emerging role of microbial natural products in antibiotic discovery. Antonie Van Leeuwenhoek.

[142] Projan S.J. (2003). Why is big Pharma getting out of antibacterial drug discovery?. Curr. Opin. Microbiol..

[143] Bax R., Mullan N., Verhoef J. (2000). The millennium bugs—The need for and development of new antibacterials. Int. J. Antimicrob. Agents.

[144] Zubair M., Alarif W., Al-Footy K., Ph M., Ali M., Basaif S., Al-Lihaibi S., Ayyad S.-E. (2016). New antimicrobial biscembrane hydrocarbon and cembranoid diterpenes from the soft coral *Sarcophyton trocheliophorum*. Turk. J. Chem..

[145] Lou Z., Sun Y., Rao Z. (2014). Current progress in antiviral strategies. Trends Pharmacol. Sci..

[146] Lurain N.S., Chou S. (2010). Antiviral drug resistance of human cytomegalovirus. Clin. Microbiol. Rev..

[147] Tantillo C., Ding J., Jacobo-Molina A., Nanni R.G., Boyer P.L., Hughes S.H., Pauwels R., Andries K., Janssen P.A.J., Arnold E. (1994). Locations of Anti-AIDS Drug Binding Sites and Resistance Mutations in the Three-dimensional Structure of HIV-1 Reverse Transcriptase: Implications for Mechanisms of Drug Inhibition and Resistance. J. Mol. Biol..

[148] Morfin F., Thouvenot D. (2003). Herpes simplex virus resistance to antiviral drugs. J. Clin. Virol..

[149] Wang S.K., Hsieh M.K., Duh C.Y. (2013). New diterpenoids from soft coral *Sarcophyton ehrenbergi*. Mar. Drugs.

[150] Tseng Y.J., Wang S.K., Duh C.Y. (2013). Secosteroids and norcembranoids from the soft coral *Sinularia nanolobata*. Mar. Drugs.

[151] Cheng S.Y., Wang S.K., Duh C.Y. (2014). Secocrassumol, a seco-cembranoid from the Dongsha Atoll soft coral *Lobophytum crassum*. Mar. Drugs.

[152] Yuan W., Cheng S., Fu W., Zhao M., Li X., Cai Y., Dong J., Huang K., Gustafson K.R., Yan P. (2016). Structurally diverse metabolites from the soft coral *Sinularia verruca* collected in the South China sea. J. Nat. Prod..

[153] Yebra D.M., Kiil S., Dam-Johansen K. (2004). Antifouling technology—past, present and future steps towards efficient and environmentally friendly antifouling coatings. Prog. Org. Coat..

[154] Davidson I.C., Brown C.W., Sytsma M.D., Ruiz G.M. (2009). The role of containerships as transfer mechanisms of marine biofouling species. Biofouling.

[155] Schultz M.P., Bendick J.A., Holm E.R., Hertel W.M. (2011). Economic impact of biofouling on a naval surface ship. Biofouling.

[156] Omae I. (2003). Organotin antifouling paints and their alternatives. Appl. Organomet. Chem..

[157] Qian P.-Y., Xu Y., Fusetani N. (2009). Natural products as antifouling compounds: Recent progress and future perspectives. Biofouling.

[158] Konstantinou I.K., Albanis T.A. (2004). Worldwide occurrence and effects of antifouling paint booster biocides in the aquatic environment: A review. Environ. Int..

[159] Cresswell T., Richards J.P., Glegg G.A., Readman J.W. (2006). The impact of legislation on the usage and environmental concentrations of Irgarol 1051 in UK coastal waters. Mar. Pollut. Bull..

[160] Muñoz I., Martínez Bueno M.J., Agüera A., Fernández-Alba A.R. (2010). Environmental and human health risk assessment of organic micro-pollutants occurring in a Spanish marine fish farm. Environ. Pollut..

[161] Kwong T.F.N., Miao L., Li X., Qian P.Y. (2006). Novel Antifouling and Antimicrobial Compound from a Marine-Derived Fungus *Ampelomyces* sp.. Mar. Biotechnol..

[162] Shi H., Yu S., Liu D., van Ofwegen L., Proksch P., Lin W. (2012). Sinularones A-I, new cyclopentenone and butenolide derivatives from a marine soft coral *Sinularia* sp. and their antifouling activity. Mar. Drugs.

[163] Chen D., Chen W., Liu D., van Ofwegen L., Proksch P., Lin W. (2013). Asteriscane-type sesquiterpenoids from the soft coral *Sinularia capillosa*. J. Nat. Prod..

[164] Lai D., Geng Z., Deng Z., van Ofwegen L., Proksch P., Lin W. (2013). Cembranoids from the soft coral *Sinularia rigida* with antifouling activities. J. Agric. Food Chem..

[165] Zhang J., Li L.-C., Wang K.-L., Liao X.-J., Deng Z., Xu S.-H. (2013). Pentacyclic hemiacetal sterol with antifouling and cytotoxic activities from the soft coral *Nephthea* sp.. Bioorg. Med. Chem. Lett..

[166] Zhang J., Liang Y., Wang K.L., Liao X.J., Deng Z., Xu S.H. (2014). Antifouling steroids from the South China Sea gorgonian coral *Subergorgia suberosa*. Steroids.

[167] Li Y.-X., Wu H.-X., Xu Y., Shao C.-L., Wang C.-Y., Qian P.-Y. (2013). Antifouling Activity of Secondary Metabolites Isolated from Chinese Marine Organisms. Mar. Biotechnol..

[168] Wang J., Su P., Gu Q., Li W.D., Guo J.L., Qiao W., Feng D.Q., Tang S.A. (2017). Antifouling activity against bryozoan and barnacle by cembrane diterpenes from the soft coral *Sinularia flexibilis*. Int. Biodeterior. Biodegrad..

[169] Quang T.H., Ha T.T., Minh C.V., Kiem P.V., Huong H.T., Ngan N.T., Nhiem N.X., Tung N.H., Thao N.P., Thuy D.T. (2011). Cytotoxic and PPARs transcriptional activities of sterols from the Vietnamese soft coral *Lobophytum laevigatum*. Bioorg. Med. Chem. Lett..

[170] Liang L.-F., Kurtán T., Mándi A., Gao L.-X., Li J., Zhang W., Guo Y.-W. (2014). Sarsolenane and capnosane diterpenes from the Hainan soft coral *Sarcophyton trocheliophorum* Marenzeller as PTP1B Inhibitors. Eur. J. Org. Chem..

[171] Liu M., Zhou Q., Wang J., Liu J., Qi C., Lai Y., Zhu H., Xue Y., Hu Z., Zhang Y. (2018). Anti-inflammatory butenolide derivatives from the coral-derived fungus *Aspergillus terreus* and structure revisions of aspernolides D and G, butyrolactone VI and 4′,8′′-diacetoxy butyrolactone VI. RSC Adv..

[172] Zheng C.J., Shao C.L., Guo Z.Y., Chen J.F., Deng D.S., Yang K.L., Chen Y.Y., Fu X.M., She Z.G., Lin Y.C. (2012). Bioactive hydroanthraquinones and anthraquinone dimers from a soft coral-derived *Alternaria* sp. fungus. J. Nat. Prod..

[173] Li H.J., Xie Y.L., Xie Z.L., Chen Y., Lam C.K., Lan W.J. (2012). Chondrosterins A-E, triquinane-type sesquiterpenoids from soft coral-associated fungus *Chondrostereum* sp.. Mar. Drugs.

[174] Fu P., Kong F., Wang Y., Wang Y., Liu P., Zuo G., Zhu W. (2013). Antibiotic metabolites from the coral-associated Actinomycete *Streptomyces* sp. OUCMDZ-1703. Chin. J. Chem..

[175] Wang W., Liao Y., Tang C., Huang X., Luo Z., Chen J., Cai P. (2017). Cytotoxic and antibacterial compounds from the coral-derived fungus *Aspergillus tritici* SP2-8-1. Mar. Drugs.

[176] Wei M.Y., Li D., Shao C.L., Deng D.S., Wang C.Y. (2013). (±)-Pestalachloride D, an antibacterial racemate of chlorinated benzophenone derivative from a soft coral-derived fungus *Pestalotiopsis* sp.. Mar. Drugs.

[177] Zheng C.J., Shao C.L., Wu L.Y., Chen M., Wang K.L., Zhao D.L., Sun X.P., Chen G.Y., Wang C.Y. (2013). Bioactive phenylalanine derivatives and cytochalasins from the soft coral-derived fungus, *Aspergillus elegans*. Mar. Drugs.

[178] Shao C.L., Xu R.F., Wang C.Y., Qian P.Y., Wang K.L., Wei M.Y. (2015). Potent antifouling marine dihydroquinolin-2(1H)-one-containing alkaloids from the Gorgonian coral-derived fungus *Scopulariopsis* sp.. Mar. Biotechnol..

[179] Jia Y.-L., Guan F.-F., Ma J., Wang C.-Y., Shao C.-L. (2015). Pestalotiolide A, a new antiviral phthalide derivative from a soft coral-derived fungus *Pestalotiopsissp*. Nat. Prod. Sci..

[180] Bao J., Sun Y.L., Zhang X.Y., Han Z., Gao H.C., He F., Qian P.Y., Qi S.H. (2013). Antifouling and antibacterial polyketides from marine gorgonian coral-associated fungus *Penicillium* sp. SCSGAF 0023. J. Antibiot..

[181] Zhuang Y., Teng X., Wang Y., Liu P., Wang H., Li J., Li G., Zhu W. (2011). Cyclopeptides and polyketides from coral-associated fungus, *Aspergillus versicolor* LCJ-5-4. Tetrahedron.

[182] Hawas U.W., El-Desouky S., Abou El-Kassem L., Elkhateeb W. (2015). Alternariol derivatives from *Alternaria alternata*, an endophytic fungus residing in red sea soft coral, inhibit HCV NS3/4A protease. Appl. Biochem. Microbiol..

[183] Zhu M., Gao H., Wu C., Zhu T., Che Q., Gu Q., Guo P., Li D. (2015). Lipid-lowering polyketides from a soft coral-derived fungus *Cladosporium* sp. TZP29. Bioorg. Med. Chem. Lett..

[184] Liu Z., Xia G., Chen S., Liu Y., Li H., She Z. (2014). Eurothiocin A and B, sulfur-containing benzofurans from a soft coral-derived fungus *Eurotium rubrum* SH-823. Mar. Drugs.

[185] Liu M., Qi C., Sun W., Shen L., Wang J., Liu J., Lai Y., Xue Y., Hu Z., Zhang Y. (2018). α-Glucosidase inhibitors from the coral-associated fungus *Aspergillus terreus*. Front. Chem..

[186] Leal C.M., Sheridan C., Osinga R., Dionísio G., Rocha J.R., Silva B., Rosa R., Calado R. (2014). Marine Microorganism-Invertebrate Assemblages: Perspectives to Solve the “Supply Problem” in the Initial Steps of Drug Discovery. Mar. Drugs.

[187] Martins A., Vieira H., Gaspar H., Santos S. (2014). Marketed marine natural products in the pharmaceutical and cosmeceutical industries: Tips for success. Mar. Drugs.

[188] Carson M.A., Clarke S.A. (2018). Bioactive Compounds from Marine Organisms: Potential for Bone Growth and Healing. Mar. Drugs.

[189] Xue-Mei H., Ru-Fang X., Yu-Cheng G., Chang-Yun W., Chang-Lun S. (2015). Biological and Chemical Diversity of Coral-Derived Microorganisms. Curr. Med. Chem..

[190] Fehmida B., Muhammad F., Esam I.A., Muhammad Y., Sana A.A., Mohammad A.K., Ikram U., Muhammad I.N. (2017). Bacteria from Marine Sponges: A Source of New Drugs. Curr. Drug Metab..

[191] Thomas T.R.A., Kavlekar D.P., LokaBharathi P.A. (2010). Marine drugs from sponge-microbe association--a review. Mar. Drugs.

[192] Raimundo I., Silva S.G., Costa R., Keller-Costa T. (2018). Bioactive Secondary Metabolites from Octocoral-Associated Microbes-New Chances for Blue Growth. Mar. Drugs.

[193] Keller-Costa T., Eriksson D., Gonçalves J.M.S., Gomes N.C.M., Lago-Lestón A., Costa R. (2017). The gorgonian coral Eunicella labiata hosts a distinct prokaryotic consortium amenable to cultivation. FEMS Microbiol. Ecol..

[194] Koren O., Rosenberg E. (2006). Bacteria associated with mucus and tissues of the coral *Oculina patagonica* in summer and winter. Appl. Environ. Microbiol..

[195] Nai C., Meyer V. (2018). From Axenic to Mixed Cultures: Technological Advances Accelerating a Paradigm Shift in Microbiology. Trends Microbiol..

[196] Brakhage A.A. (2012). Regulation of fungal secondary metabolism. Nat. Rev. Microbiol..

[197] Seyedsayamdost M.R. (2014). High-throughput platform for the discovery of elicitors of silent bacterial gene clusters. Proc. Natl. Acad. Sci. USA.

[198] Benkendorff K., Burnell G., Allan G. (2009). 28—Aquaculture and the production of pharmaceuticals and nutraceuticals. New Technologies in Aquaculture.

[199] Pomeroy R.S., Parks J.E., Balboa C.M. (2006). Farming the reef: Is aquaculture a solution for reducing fishing pressure on coral reefs?. Mar. Policy.

[200] Leal M.C., Calado R., Sheridan C., Alimonti A., Osinga R. (2013). Coral aquaculture to support drug discovery. Trends Biotechnol..

[201] Mendola D. (2003). Aquaculture of three phyla of marine invertebrates to yield bioactive metabolites: Process developments and economics. Biomol. Eng..

[202] Loureiro C., Medema M.H., van der Oost J., Sipkema D. (2018). Exploration and exploitation of the environment for novel specialized metabolites. Curr. Opin. Biotechnol..

[203] Faisal M., Saeed A., Shahzad D., Brahmachari G. (2019). Chapter 5–Portrait of the synthesis of some potent anti-inflammatory natural products. Discovery and Development of Anti-Inflammatory Agents from Natural Products.

[204] Barbosa L.C.A., Varejão J.O.S., Varejão E.V.V., Atta-ur R. (2017). Chapter 3—Strategies for Total Synthesis of Furanocembranolides and Related Natural Products from Marine Organisms. Studies in Natural Products Chemistry.

[205] Nicolaou K.C., Xu J.Y., Kim S., Pfefferkorn J., Ohshima T., Vourloumis D., Hosokawa S. (1998). Total Synthesis of Sarcodictyins A and B. J. Am. Chem. Soc..

[206] Nicolaou K.C., Xu J.Y., Kim S., Ohshima T., Hosokawa S., Pfefferkorn J. (1997). Synthesis of the Tricyclic Core of Eleutherobin and Sarcodictyins and Total Synthesis of Sarcodictyin A. J. Am. Chem. Soc..

[207] Nicolaou K.C., van Delft F., Ohshima T., Vourloumis D., Xu J., Hosokawa S., Pfefferkorn J., Kim S., Li T. (1997). Total Synthesis of Eleutherobin. Angew. Chem. Int. Ed. Engl..

[208] Nicolaou K.C., Ohshima T., Hosokawa S., van Delft F.L., Vourloumis D., Xu J.Y., Pfefferkorn J., Kim S. (1998). Total Synthesis of Eleutherobin and Eleuthosides A and B. J. Am. Chem. Soc..

[209] Nicolaou K.C., Kim S., Pfefferkorn J., Xu J., Ohshima T., Hosokawa S., Vourloumis D., Li T. (1998). Synthesis and Biological Activity of Sarcodictyins. Angew. Chem. Int. Ed..

