# Supplementary material for: Coral and Coral-Associated Microorganisms: A Prolific Source of Potential Bioactive Natural Products
Source: Mar Drugs. 2019 Aug 11;17(8):468. doi: 10.3390/md17080468 (PMC6723858; doi:10.3390/md17080468)
Supplement: Supplementary file 1 [file marinedrugs-17-00468-s001.pdf]

List of supplementary tables about bioactive compounds from coral and its associated microorganisms

Supplementary table S1. Anti-inflammatory compounds from coral

Supplementary table S2. Cytotoxic compounds from coral

Supplementary table S3. Antimicrobial compounds from coral

Supplementary table S4. Antiviral compounds from coral

Supplementary table S5. Antifouling compounds from coral

Supplementary table S6. Other bioactive compounds from coral

Supplementary table S7. Anti-inflammatory and cytotoxic compounds from coral-associated microorganisms

Supplementary table S8. Antimicrobial compounds from coral-associated microorganisms

Supplementary table S9. Antiviral compounds from coral-associated microorganisms

Supplementary table S10. Antifouling compounds from coral-associated microorganisms

Supplementary table S11. Other bioactive compounds from coral-associated microorganisms

Supplementary table S1. Anti-inflammatory compounds from coral

Compounds	Coral	Anti-inflammatory mechanism	Activity values	Ref.
Lobophytone D	*Lobophytum pauciflorum*	Inhibiting LPS-induced NO release	IC_{50} = 4.70 μM	[1]
Flexibilisolide A	*Sinularia* sp.	Inhibiting LPS-induced iNOS	Reduce iNOS protein to 19.4 ± 4.5% at 10 μM	[2]
Flexilarin	*Sinularia* sp.	Inhibiting LPS-induced iNOS	Reduce iNOS protein to 13.8 ± 2.1% at 10 μM	[2]
Gyrosanol A	*Sinularia gyrosa*	Inhibiting LPS-induced COX-2	Inhibition percentage: 19.6 ± 3.9% at 10 μM	[3]
Gyrosanol B	*Sinularia gyrosa*	Inhibiting LPS-induced COX-2	Inhibition percentage: 29.1 ± 9.6% at 10 μM	[3]
Hirsutalin B	*Cladiella hirsuta*	Inhibiting LPS-induced iNOS, COX-2	Reduce iNOS and COX-2 protein to 6.8 ± 0.6% and 49.0 ± 2.3%, respectively at 10 μM	[4]
Hirsutalin C	*Cladiella hirsuta*	Inhibiting LPS-induced iNOS	Reduce iNOS protein to 43.6 ± 8.7% at 10 μM	[4]
Hirsutalin D	*Cladiella hirsuta*	Inhibiting LPS-induced iNOS	Reduce iNOS protein to 3.3 ± 0.1% at 10 μM	[4]
Hirsutalin H	*Cladiella hirsuta*	Inhibiting LPS-induced iNOS	Reduce iNOS protein to 32.3 ± 6.1% at 10 μM	[4]
Cespitularin S	*Cespitularia hypotentaculata*	Inhibiting LPS-induced iNOS, COX-2	Reduce iNOS and COX-2 protein to 52.7 ±3.2% and 78.3 ±0.6%, respectively at 10 μM	[5]
Cespitularin I	*Cespitularia hypotentaculata*	Inhibiting LPS-induced iNOS	Reduce iNOS protein to 86.7± 8.4% at 10 μM	[5]
Cespitularin F	*Cespitularia hypotentaculata*	Inhibiting LPS-induced iNOS	Reduce iNOS protein to 72.0 ±15.1% at 10 μM	[5]
Klysimplexin R	*Klyxum simplex*	Inhibiting LPS-induced iNOS and COX-2	Reduce iNOS and COX-2 protein to < 20% and < 50%, respectively at 10 μM	[6]
Klysimplexin S	*Klyxum simplex*	Inhibiting LPS-induced iNOS and COX-2	Reduce iNOS and COX-2 protein to < 10% and < 40%, respectively at 10 μM	[6]
Klysimplexin J	*Klyxum simplex*	Inhibiting LPS-induced iNOS	Reduce iNOS protein to < 60% at 10 μM	[6]
Klysimplexin K	*Klyxum simplex*	Inhibiting LPS-induced iNOS	Reduce iNOS protein to < 40% at 10 μM	[6]
Klysimplexin L	*Klyxum simplex*	Inhibiting LPS-induced iNOS	Reduce iNOS protein to < 30% at 10 μM	[6]
Klysimplexin M	*Klyxum simplex*	Inhibiting LPS-induced iNOS	Reduce iNOS protein to < 20% at 10 μM	[6]
Klysimplexin N	*Klyxum simplex*	Inhibiting LPS-induced iNOS	Reduce iNOS protein to < 40% at 10 μM	[6]
Ximaolide F	*Lobophytum laevigatum*	Inhibiting TNFα-induced NF-κB transcriptional activation	IC_{50} = 6.9 μM	[7]
Methyl tortuoate B	*Lobophytum laevigatum*	Inhibiting TNFα-induced NF-κB transcriptional activation	IC_{50} = 6.7 μM	[7]
Laevigatol A	*Lobophytum laevigatum*	Inhibiting TNFα-induced NF-κB transcriptional activation	IC_{50} = 9.4 μM	[7]

Laevigatol B	*Lobophytum laevigatum*	Inhibiting TNFα-induced NF-κB transcriptional activation	IC_{50} = 9.7 μM	[7]
Sinularin	*Sinularia triangular*<br>*Sinularia flexibilis*	Inhibiting LPS-induced iNOS and COX-2; Inhibiting LPS-induced NO release and TNF-α	Reduce iNOS protein to 1.2 ± 0.3% at 10 μM; 88.60% inhibition (NO) and 94.20% inhibition (TNF-α) at 10 μM	[8,9]
Dihydrosinularin	*Sinularia triangular*	Inhibiting LPS-induced iNOS and COX-2	Reduce iNOS and COX-2 protein to 5.1 ± 1.6% and 24.9 ± 7.4%, respectively at 10 μM	[8]
(−)14-deoxycrassin	*Sinularia triangular*	Inhibiting LPS-induced iNOS and COX-2	Reduce iNOS and COX-2 protein to 0.9 ± 0.7% and 5.9 ± 1.0%, respectively at 10 μM	[8]
Lobocrassin B	*Lobophytum crassum*	Inhibiting superoxide anion generation and elastase release in FMLP/CB-induced human neutrophils	IC_{50} = 4.8 ± 0.7 (superoxide anion) and 4.9 ± 0.4 μM (elastase release)	[10]
Crassarine F	*Sinularia crassa*	Inhibiting LPS-induced COX-2	Reduce COX-2 protein to 65.6 ± 6.2% at 10 μM	[11]
Crassarine H	*Sinularia crassa*	Inhibiting LPS-induced iNOS	Reduce iNOS protein to 35.8 ± 10.7% at 10 μM	[11]
Krempfielin B	*Cladiella krempfi*	Inhibiting LPS-induced iNOS	Reduce iNOS protein to < 40% at 10 μM	[12]
Krempfielin C	*Cladiella krempfi*	Inhibiting LPS-induced iNOS	Reduce iNOS protein to < 60% at 10 μM	[12]
Krempfielin D	*Cladiella krempfi*	Inhibiting LPS-induced iNOS	Reduce iNOS protein to nearly 60% at 10 μM	[12]
Litophynol B	*Cladiella krempfi*	Inhibiting LPS-induced iNOS	Reduce iNOS protein to < 40% at 10 μM	[12]
(1*R**,2*R**,3*R**,6*S**,7*S**,9*R**,10*R**,14*R**)-3-butanoyloxycladiell-11(17)-en-6,7-diol	*Cladiella krempfi*	Inhibiting LPS-induced iNOS	Reduce iNOS protein to nearly 10% at 10 μM	[12]
Paraminabeolide B	*Paraminabea acronocephala*	Inhibiting LPS-induced iNOS	Reduce iNOS protein to 7.3 ± 1.0% at 10 μM	[13]
Paraminabeolide C	*Paraminabea acronocephala*	Inhibiting LPS-induced iNOS	Reduce iNOS protein to 37.9 ± 9.9% at 10 μM	[13]
Paraminabeolide D	*Paraminabea acronocephala*	Inhibiting LPS-induced iNOS	Reduce iNOS protein to 43.4 ± 9.5% at 10 μM	[13]
Minabeolide-1	*Paraminabea acronocephala*	Inhibiting LPS-induced iNOS and COX-2	Reduce iNOS and COX-2 protein to 9.6 ± 1.9% and 18.3± 7.2%, respectively at 10 μM	[13]
Minabeolide-2	*Paraminabea acronocephala*	Inhibiting LPS-induced iNOS and COX-2	Reduce iNOS and COX-2 protein to 45.7 ± 7.7% and 51.2 ±11.5%, respectively at 10 μM	[13]
Minabeolide-4	*Paraminabea acronocephala*	Inhibiting LPS-induced iNOS and COX-2	Reduce iNOS and COX-2 protein to 23.2 ± 4.6% and 22.4 ± 9.9%, respectively at 10 μM	[13]
Minabeolide-5	*Paraminabea acronocephala*	Inhibiting LPS-induced iNOS and COX-2	Reduce iNOS and COX-2 protein to 6.3 ± 1.5% and 31.3 ± 10.7%, respectively at 10 μM	[13]

Paraminabeolide A	*Paraminabea acronocephala*	Inhibiting LPS-induced iNOS	Reduce iNOS protein to 11.0 ± 7.7% at 10 μM	[13]
Paraminabeolide B	*Paraminabea acronocephala*	Inhibiting LPS-induced iNOS	Reduce iNOS protein to 7.3 ± 1.0% at 10 μM	[13]
Paraminabeolide C	*Paraminabea acronocephala*	Inhibiting LPS-induced iNOS	Reduce iNOS protein to 37.9 ± 9.9% at 10 μM	[13]
Paraminabeolide D	*Paraminabea acronocephala*	Inhibiting LPS-induced iNOS	Reduce iNOS protein to 43.4 ± 9.5% at 10 μM	[13]
Klymollin C	*Klyxum molle*	Inhibiting LPS-induced iNOS	Reduce iNOS protein to 42.1 ± 11.5% at 10 μM	[14]
Klymollin D	*Klyxum molle*	Inhibiting LPS-induced iNOS	Reduce iNOS protein to 25.1 ± 8.7% at 10 μM	[14]
Klymollin E	*Klyxum molle*	Inhibiting LPS-induced iNOS	Reduce iNOS protein to 25.7 ± 8.0% at 10 μM	[14]
Klymollin F	*Klyxum molle*	Inhibiting LPS-induced iNOS and COX-2	Reduce iNOS and COX-2 protein to 6.0 ± 2.6% and 8.5 ± 1.3%, respectively at 10 μM	[14]
Klymollin G	*Klyxum molle*	Inhibiting LPS-induced iNOS and COX-2	Reduce iNOS and COX-2 protein to 5.2 ± 2.5% and 4.4 ± 1.3%, respectively at 10 μM	[14]
Klymollin H	*Klyxum molle*	Inhibiting LPS-induced iNOS	At a concentration of 10 μM, reduce the level of iNOS protein to 32.6 ± 11.8% at 10 μM	[14]
Sarcocrassocolide I	*Sarcophyton crassocaule*	Inhibiting LPS-induced iNOS and COX-2	Reduce iNOS and COX-2 protein to < 10% and < 60%, respectively at 10 μM	[15]
Sarcocrassocolide F	*Sarcophyton crassocaule*	Inhibiting LPS-induced iNOS	Reduce iNOS protein to < 10% at 10 μM	[15]
Sarcocrassocolide G	*Sarcophyton crassocaule*	Inhibiting LPS-induced iNOS	Reduce iNOS protein to < 10% at 10 μM	[15]
Sarcocrassocolide H	*Sarcophyton crassocaule*	Inhibiting LPS-induced iNOS	Reduce iNOS protein to < 10% at 10 μM	[15]
Sarcocrassocolide J	*Sarcophyton crassocaule*	Inhibiting LPS-induced iNOS	Reduce iNOS protein to < 10% at 10 μM	[15]
Sarcocrassocolide K	*Sarcophyton crassocaule*	Inhibiting LPS-induced iNOS	Reduce iNOS protein to < 10% at 10 μM	[15]
Sarcocrassocolide L	*Sarcophyton crassocaule*	Inhibiting LPS-induced iNOS	Reduce iNOS protein to < 10% at 10 μM	[15]
Sinularioside	*Sinularia* sp.	Inhibiting LPS-induced NO release	58% inhibition at 30 μM	[16]
Cerebroside	*Sinularia* sp.	Inhibiting LPS-induced NO release	16% inhibition at 30 μM	[16]
Scabralin A	*Sinularia scabra*	Inhibiting LPS-induced iNOS	Reduce iNOS protein to 39.1 ± 15.9% at 10 μM	[17]
Echinolabdane A	*Echinomuricea* sp.	Inhibiting superoxide anion generation and elastase release in FMLP/CB-induced human neutrophils	68.6% and 35.4% inhibition at 10 μM	[18]
6-*epi*-yonarasterol B	*Echinomuricea* sp.	Inhibiting superoxide anion generation and elastase release in FMLP/CB-induced human neutrophils	IC_{50} = 2.98 ± 0.29 và 1.13 ± 0.55 μg/mL	[18]
Lochmolin A	*Sinularia lochmodes*	Inhibiting LPS-induced COX-2	Reduce COX-2 protein to 1.7 ± 1.3% at 100 μM	[19]

Lochmolin B	*Sinularia lochmodes*	Inhibiting LPS-induced COX-2	Reduce COX-2 protein to 17.6 ± 2.2% at 100 μM	[19]
Lochmolin C	*Sinularia lochmodes*	Inhibiting LPS-induced COX-2	Reduce COX-2 protein to 32.8 ± 3.2% at 100 μM	[19]
Lochmolin D	*Sinularia lochmodes*	Inhibiting LPS-induced COX-2	Reduce COX-2 protein to 71.3 ± 7.2% at 100 μM	[19]
Crassarosteroside A	*Sinularia crassa*	Inhibiting LPS-induced iNOS	Reduce iNOS protein to 12.9 ± 4.3% at 10 μM	[20]
Crassarosteroside C	*Sinularia crassa*	Inhibiting LPS-induced iNOS	Reduce iNOS protein to 50.1 ± 6.3% at 10 μM	[20]
Sarcocrassocolide M	*Sarcophyton crassocaule*	Inhibiting LPS-induced iNOS and COX-2	Reduce iNOS and COX-2 protein to 4.2 ± 1.6% and 62.8 ± 22.4%, respectively at 10 μM	[21]
Sarcocrassocolide N	*Sarcophyton crassocaule*	Inhibiting LPS-induced iNOS and COX-2	Reduce iNOS and COX-2 protein to 4.2 ± 1.6% and 52.9 ± 12.8%, respectively at 10 μM	[21]
Sarcocrassocolide O	*Sarcophyton crassocaule*	Inhibiting LPS-induced iNOS and COX-2	Reduce iNOS and COX-2 protein to 4.2 ± 1.6% and 22.7 ± 2.8%, respectively at 10 μM	[21]
Sclerosteroid A	*Scleronephthya gracillimum*	Inhibiting LPS-induced iNOS and COX-2	Reduce iNOS and COX-2 protein to 28.4% and 5.4%, respectively at 10 μM	[22]
Sclerosteroid B	*Scleronephthya gracillimum*	Inhibiting LPS-induced iNOS and COX-2	Reduce iNOS and COX-2 protein to 27.7% and 6.7%, respectively at 10 μM	[22]
Sclerosteroid E	*Scleronephthya gracillimum*	Inhibiting LPS-induced iNOS and COX-2	Reduce iNOS and COX-2 protein to 25.4% and 20.6%, respectively at 10 μM	[22]
8*α*H-3*β*,11-dihydroxy-5*α*,6*α*-expoxy-24-methylene-9,11-secocholestan-9-one	*Sinularia granosa*	Inhibiting LPS-induced iNOS and COX-2	Reduce iNOS and COX-2 protein to 66.1 ± 11.9% and 42.7 ± 16.5%, respectively at 10 μM	[23]
3*β*,11-dihydroxy-5*β*,6*β*-expoxy-24-methylene-9,11-secocholestan-9-one	*Sinularia granosa*	Inhibiting LPS-induced iNOS	Reduce iNOS protein to 19.4 ± 2.1% at 10 μM	[23]
Echinoclerodane A	*Echinomuricea* sp.	Inhibiting the superoxide anion generation and elastase release in FMLP/CB-induced human neutrophils	68.6% inhibition (superoxide anion) and 35.4% inhibition (elastase) at 10 μg/mL	[24]
6-*epi*-cladieunicellin F	*Cladiella* sp.	Inhibiting the superoxide anion generation in FMLP/CB-induced human neutrophils	IC_{50} = 6.57 ± 0.85 μg/mL	[25]
Flexibilide	*Sinularia* sp.	Inhibition of NF-κB activation	IC_{50} = 5.30 μg/mL	[26]
Capillosanane B	*Sinularia capillosa*	Inhibiting production of TNF-*α*	16% inhibition at 10 μM	[27]
Capillosanane I	*Sinularia capillosa*	Inhibiting production of TNF-*α*	21% inhibition at 10 μM	[27]
(−)-Sinularone A	*Sinularia capillosa*	Inhibiting production of TNF-*α*	23% inhibition at 10 μM	[27]
6-acetoxy litophynin E	*Cladiella krempfi*	Inhibiting LPS-induced iNOS	Reduce iNOS protein to 12.8 ± 2.9% at 10 μM	[28]

6-methyl ether of litophynol B	*Cladiella krempfi*	Inhibiting LPS-induced iNOS and COX-2	Reduce iNOS and COX-2 protein to 6.4 ± 0.8% and 52.5 ± 8.0%, respectively at 10 μM	[28]
Scabrolide A	*Sinularia maxima*	Inhibiting LPS-induced IL-12 and IL-6	IC_{50} = 23.52 ± 1.37 and 69.85 ± 4.11 μM	[29]
13-*epi*-scabrolide C	*Sinularia maxima*	Inhibiting LPS-induced IL-12 and IL-6	IC_{50} = 5.30 ± 0.21 and 13.12 ± 0.64 μM	[29]
(22*R*,23*R*,24*R*)-5*α*,8*α*-epidioxy-22,23-methylene-24-methylcholest-6-en-3*β*-ol	*Lobophytum crassum*	Inhibiting TNF*α*-induced NF-κB transcriptional activation	IC_{50} = 3.90 μM	[30]
Ergosterol peroxide	*Lobophytum crassum*	Inhibiting TNF*α*-induced NF-κB transcriptional activation	IC_{50} =7.05 μM	[30]
Sclerosteroid J	*Scleronephthya gracillimum*	Inhibiting LPS-induced iNOS and COX-2	Reduce iNOS and COX-2 protein to 72.8 ± 9.5% and 28.4 ± 4.9%, respectively at 10 μM	[31]
Sclerosteroid K	*Scleronephthya gracillimum*	Inhibiting LPS-induced iNOS and COX-2	Reduce iNOS and COX-2 protein to 28.4 ± 8.4% and 9.0 ± 4.4%, respectively at 10 μM	[31]
Sclerosteroid M	*Scleronephthya gracillimum*	Inhibiting LPS-induced iNOS and COX-2	Reduce iNOS and COX-2 protein to 27.2 ± 9.0% and 11.8 ± 6.8%, respectively at 10 μM	[31]
Sclerosteroid N	*Scleronephthya gracillimum*	Inhibiting LPS-induced iNOS and COX-2	Reduce iNOS and COX-2 protein to 60.3 ± 9.7% and 26.6 ± 10.0%, respectively at 10 μM	[31]
3-methyl-5-(10′-acetoxy-2′,6′,10′-trimethylundecyl)-2-penten-5-olide	*Scleronephthya gracillimum*	Inhibiting LPS-induced iNOS and COX-2	Reduce iNOS and COX-2 protein to 61.8 ± 9.8% and 61.7 ± 8.3%, respectively at 10 μM	[31]
Sinularcasbane B	*Sinularia* sp.	Inhibiting LPS-induced NO release	IC_{50} = 8.3 μM	[32]
Sinularcasbanes E	*Sinularia* sp.	Inhibiting LPS-induced NO release	IC_{50} = 5.4 μM	[32]
Hyperinakin	*Hypericum nakamurai*	Inhibiting LPS-induced NO release	IC_{50} = 20 μM	[33]
3-geranyl-2,4,6-trihydroxybenzophenone	*Hypericum nakamurai*	Inhibiting LPS-induced NO release	IC_{50} = 30 μM	[33]
Paraminabic acid A	*Paraminabea acronocephala*	Inhibiting LPS-induced iNOS	Reduce iNOS protein to 63.9 ±6.3% at 10 μM	[34]
Paraminabic acid B	*Paraminabea acronocephala*	Inhibiting LPS-induced iNOS	53.5 ± 8.6% inhibition at 10 μM	[34]
Flexibilisquinone	*Sinularia flexibilis*	Inhibiting LPS-induced iNOS and COX-2	Reduce iNOS and COX-2 protein to < 50% and nearly 80%, respectively at 10 μM	[35]
Krempfielin K	*Cladiella krempfi*	Inhibiting elastase release in FMLP/CB-induced human neutrophils	45.51 ± 2.69% inhibition at 10 μM	[36]
Krempfielin M	*Cladiella krempfi*	Inhibiting the elastase release in FMLP/CB-induced human neutrophils	27.30 ± 5.42% inhibition at 10 μM	[36]
Klymollin M	*Klyxum molle*	Inhibiting the superoxide anion generation and	IC_{50} = 3.13 ± 0.39 μM (superoxide anion) and 2.92 ±	[37]

		elastase release in FMLP/CB-induced human neutrophils	0.27 μM (elastase)	
Litophynin F	*Cladiella krempfi*	Inhibiting LPS-induced COX-2	Reduce COX-2 protein to 48.1 ± 10.8% 10 μM	[28]
Hirsutalin K	*Cladiella hirsuta*	Inhibiting LPS-induced iNOS	IC_{50} = 9.8 mg/mL	[38]
Sinularianin A	*Sinularia* sp.	Inhibition of NF-κB activation	41.3% inhibition at 10 μg/mL	[39]
Sinularianin B	*Sinularia* sp.	Inhibition of NF-κB activation	29.6% inhibition at 10 μg/mL	[39]
Sinularianin C	*Sinularia* sp.	Inhibition of NF-κB activation	24.3% inhibition at 10 μg/mL	[39]
Sinularianin D	*Sinularia* sp.	Inhibition of NF-κB activation	43.0% inhibition at 10 μg/mL	[39]
Sinularianin E	*Sinularia* sp.	Inhibition of NF-κB activation	30.0% inhibition at 10 μg/mL	[39]
Sinularianin F	*Sinularia* sp.	Inhibition of NF-κB activation	36.1% inhibition at 10 μg/mL	[39]
Lobocrasol A	*Lobophytum crassum*	Inhibiting TNFα-induced NF-κB transcriptional activation	IC_{50} = 6.30 ± 0.42 μM	[40]
Lobocrasol B	*Lobophytum crassum*	Inhibiting TNFα-induced NF-κB transcriptional activation	IC_{50} = 6.63 ± 0.11 μM	[40]
Crassumol E	*Lobophytum crassum*	Inhibiting TNFα-induced NF-κB transcriptional activation	IC_{50} = 9.23± 1.66 μM	[41]
(1*R*,4*R*,2*E*,7*E*,11*E*)- cembra-2,7,11-trien-4-ol	*Lobophytum crassum*	Inhibiting TNFα-induced NF-κB transcriptional activation	IC_{50} = 1.65± 0.2 μM	[41]
Sinumaximol A	*Sinularia maxima*	Inhibiting TNFα-induced NF-κB transcriptional activation	IC_{50} = 21.35 ± 3.21 μM	[42]
Sinumaximol B	*Sinularia maxima*	Inhibiting TNFα-induced NF-κB transcriptional activation	IC_{50} = 29.10 ± 1.54 μM	[42]
Sethukarailin	*Sinularia maxima*	Inhibiting TNFα-induced NF-κB transcriptional activation	IC_{50} = 25.81 ± 1.38 μM	[42]
Sinumaximol G	*Sinularia maxima*	Inhibiting TNFα-induced NF-κB transcriptional activation	IC_{50} = 15.81 ± 2.29 μM	[42]
Yonarolide	*Sinularia maxima*	Inhibiting TNFα-induced NF-κB transcriptional activation	IC_{50} = 25.1 ± 2.58 μM	[42]
5-epinorcembrene	*Sinularia maxima*	Inhibiting TNFα-induced NF-κB transcriptional activation	IC_{50} = 28.19 ± 2.65 μM	[42]
13-*epi*-scabrolide C	*Sinularia maxima*	Inhibiting TNFα-induced NF-κB transcriptional activation	IC50 = 20.13 ± 0.29 μM	[42]
Rumphellol A	*Rumphella antipathies*	Inhibiting superoxide anion generation and elastase release in FMLP/CB-induced human	31.95% inhibition (superoxide anion) and 51.64% inhibition (elastase) at 10 μg/mL	[43]

		neutrophils		
Rumphellol B	*Rumphella antipathies*	Inhibiting superoxide anion generation and elastase release in FMLP/CB-induced human neutrophils	42.22% inhibition (superoxide anion) and 42.10% inhibition (elastase) at 10 μg/mL	[43]
Briaviolide E	*Briareum violacea*	Inhibiting superoxide anion generation and elastase release in FMLP/CB-induced human neutrophils	34.17 ±0.79% inhibition (superoxide anion) and 26.03 ±9.51% inhibition (elastase) at 10 μg/mL	[44]
Briaviolide I	*Briareum violacea*	Inhibiting superoxide anion generation and elastase release in FMLP/CB-induced human neutrophils	28.66 ±1.99% inhibition (superoxide anion) and 28.81 ±6.37% inhibition (elastase) at 10 μg/mL	[44]
Derivative	*Briareum violacea*	Inhibiting the elastase release in FMLP/CB-induced human neutrophils	28.60 ±7.54% inhibition at 10 μg/mL	[44]
Rumphellaoic acid A	*Rumphella antipathies*	Inhibiting the elastase release in FMLP/CB-induced human neutrophils	29.2% inhibition at 10 μg/mL	[45]
Sarcopanol A	*Sarcophyton pauciplicatum*	Inhibiting TNFa/INFc-induced NF-jB transcriptional activation	EC_{50} = 8.27 ± 3.28 μM	[46]
(24*S*)-ergost-1*β*,3*β*,5*α*,6*β*-tetraol-25-monoacetate	*Sarcophyton pauciplicatum*	Inhibiting TNFa/INFc-induced NF-jB transcriptional activation	EC_{50} = 26.07 ± 5.59 μM	[46]
(24*S*)-ergost-25-ene-1*β*,3*β*,5*α*,6*β*-tetraol	*Sarcophyton pauciplicatum*	Inhibiting TNFa/INFc-induced NF-jB transcriptional activation	EC_{50} = 50 μM	[46]
Krempfielin N	*Cladiella krempfi*	Inhibiting the elastase release in FMLP/CB-induced human neutrophils	IC_{50} = 4.94 ± 1.68 μM	[47]
Krempfielin O	*Cladiella krempfi*	Inhibiting the elastase release in FMLP/CB-induced human neutrophils	IC_{50} > 10 μM	[47]
Tortuosene B	*Sarcophyton tortuosum*	Inhibiting the elastase release in FMLP/CB-induced neutrophils	13.7 ± 3.5% inhibition at 10 μM	[48]
Emblide	*Sarcophyton tortuosum*	Inhibiting the elastase release in FMLP/CB-induced neutrophils	29.2 ± 6.1% inhibition at 10 μM	[48]
Sarcocrassocolide R	*Sarcophyton crassocaule*	Inhibiting LPS-induced iNOS	Reduce iNOS protein to 1.2% ± 0.3% at 10 μM	[49]
Crassocolide A	*Sarcophyton crassocaule*	Inhibiting LPS-induced iNOS and COX-2	Reduce iNOS and COX-2 protein to 3.5% ± 0.9% and 59.4% ± 21.4%, respectively at 10 μM	[49]
Crassocolide B	*Sarcophyton crassocaule*	Inhibiting LPS-induced iNOS	Reduce iNOS protein to 3.2% ± 0.7% at 10 μM	[49]
Crassocolide E	*Sarcophyton crassocaule*	Inhibiting LPS-induced iNOS and COX-2	Reduce iNOS and COX-2 protein to 1.4% ± 0.4% and 32.0% ± 15.3%, respectively at 10 μM	[49]
Sarcocrassocolide P	*Sarcophyton crassocaule*	Inhibiting LPS-induced iNOS and COX-2	Reduce iNOS protein to 1.3% ± 0.3% at 10 μM	[49]

Sarcocrassocolide Q	*Sarcophyton crassocaule*	Inhibiting LPS-induced iNOS and COX-2	Reduce iNOS and COX-2 protein to 2.4% ± 0.4% and 58.3% ± 20.5%, respectively at 10 μM	[49]
Crassocolide D	*Sarcophyton crassocaule*	Inhibiting LPS-induced iNOS and COX-2	Reduce iNOS protein to 3.2% ± 0.6% at 10 μM	[49]
Hirsutalin N	*Cladiella hirsuta*	Inhibiting the elastase release in FMLP/CB-induced human neutrophils	31.7 ± 3.2% inhibition at 10 μg/mL	[50]
Krempfielin P	*Cladiella krempfi*	Inhibiting the superoxide anion generation in FMLP/CB-induced human neutrophils	IC_{50} > 10 μM	[47]
Tortuosene A	*Sarcophyton tortuosum*	Inhibiting the superoxide anion generation in fMLP/CB-induced neutrophils	56.0 ± 3.1% inhibition at 10 μM	[48]
Klymollin X	*Klyxum molle*	Inhibiting LPS-induced IL-6	Reduce IL-6 level to nearly 60% at 25 μM	[51]
Krempfielin Q	*Cladiella krempfi*	Inhibiting the superoxide anion generation and elastase release in FMLP/CB-induced human neutrophils	5.46 ± 5.19% inhibition (superoxide anion) and 2.99 ± 2.82% inhibition (elastase) at 10 μM	[52]
Krempfielin R	*Cladiella krempfi*	Inhibiting the superoxide anion generation in FMLP/CB-induced human neutrophils	13.17% ± 2.09% inhibition (superoxide anion) and 11.09% ± 5.55% inhibition (elastase) at 10 μM	[52]
Capgermacrene A	*Capnella sp.*	Inhibiting LPS-induced NO release	28.0% and 14.2% inhibition at 10 and 20 μg/mL	[53]
Hirsutalin S	*Cladiella hirsuta*	Inhibition of superoxide anion generation and elastase release in FMLP/CB-induced human neutrophils	46.7% ± 8.0% inhibition (elastase) and 5.8% ± 0.8% inhibition (superoxide anion) at 10 μg/mL	[54]
Hirsutalin T	*Cladiella hirsuta*	Inhibition of elastase release in FMLP/CB-induced human neutrophils	19.3% ± 5.6% inhibition at 10 μg/mL	[54]
Hirsutosteroside A	*Cladiella hirsuta*	Inhibition of elastase release in FMLP/CB-induced human neutrophils	IC_{50} = 4.1 ± 0.1 μM	[55]
Sinulerectol A	*Sinularia erecta*	Inhibition of superoxide anion generation and elastase release in FMLP/CB-induced human neutrophils	IC_{50} = 2.3 ± 0.4 μM (superoxide anion) and IC_{50} = 0.9 ± 0.1 μM (elastase)	[56]
Sinulerectol B	*Sinularia erecta*	Inhibition of superoxide anion generation and elastase release in FMLP/CB-induced human neutrophils	IC_{50} = 8.5 ± 0.3 μM (superoxide anion) and IC_{50} = 3.8 ± 0.6 μM (elastase)	[56]
(*Z*)-N-[2-(4-hydroxyphenyl)ethyl]-3-methyldodec-2-enamide	*Sinularia erecta*	Inhibition of elastase release in FMLP/CB-induced human neutrophils	IC_{50} = 1.0 ± 0.2 μM	[56]
Klyflaccisteroid C	*Klyxum flaccidum*	Inhibition of superoxide anion generation and elastase release in FMLP/CB-induced human neutrophils	76.24 ± 5.64% inhibition (superoxide anion) and 88.38 ± 1.19% inhibition (elastase) at 10 μg/mL	[57]

Klyflaccisteroid F	*Klyxum flaccidum*	Inhibition of superoxide anion generation and elastase release in FMLP/CB-induced human neutrophils	88.26 ± 3.86% inhibition (superoxide anion) and 104.22 ± 6.55% inhibition (elastase) at 10 μg/mL	[57]
3*β*,11-dihydroxy-9,11-secogorgost-5-en-9-one	*Klyxum flaccidum*	Inhibition of superoxide anion generation and elastase release in FMLP/CB-induced human neutrophils	87.00 ± 1.27% inhibition (superoxide anion) and 97.42 ± 7.76% inhibition (elastase) at 10 μg/mL	[57]
Glaucumolide A	*Sarcophyton glaucum*	Inhibition of superoxide anion generation and elastase release in FMLP/CB-induced human neutrophils	88.42 ± 3.97% inhibition (superoxide anion) and 88.94 ± 6.96% inhibition (elastase) at 10 μg/mL	[58]
Glaucumolide B	*Sarcophyton glaucum*	Inhibition of superoxide anion generation and elastase release in FMLP/CB-induced human neutrophils; inhibiting LPS-induced iNOS and COX-2	91.75 ± 3.08% inhibition (superoxide anion) and 103.25 ± 1.89% inhibition (elastase) at 10 μg/mL; reduce iNOS and COX-2 protein to 75.9 ± 3.5 and 64.3 ± 6.9%; and 43.4 ± 5.0 and 6.0 ± 3.6% at 10 and 20 μM	[58]
24-methylenecholest-5-ene-3*β*,16*β*-diol-3-O-*α*-L-fucoside	*Sinularia nanolobata*	Inhibition of superoxide anion generation and elastase release in FMLP/CB-induced human neutrophils	IC_{50} = 18.6 ± 1.5 μM (superoxide anion) and IC_{50} = 10.1 ± 0.8μM (elastase)	[55]
5*β*,6*β*-epoxy-3*β*,11-dihydroxy-24-methylene9,11-secocholestan-9-one	*Sinularia nanolobata*	Inhibition of superoxide anion generation and elastase release in FMLP/CB-induced human neutrophils	IC_{50} = 6.6 ± 0.6 μM (superoxide anion) and IC_{50} = 2.9± 0.5 μM (elastase)	[55]
Columnariol A	*Nephthea columnaris*	Inhibiting LPS-induced iNOS and COX-2	52.26 ± 3.74% and 60.17 ± 7.09% at 50 μM	[59]
Columnariol B	*Nephthea columnaris*	Inhibiting LPS-induced iNOS and COX-2	24.74 ± 0.02% and 49.79 ± 3.56% at 50 μM	[59]
Sinumerolide A	*Sinularia numerosa*	Inhibiting LPS-induced iNOS	Reduce iNOS protein to 40.18 ± 6.23% at 10 μM	[60]
7*E*-sinumerolide A	*Sinularia numerosa*	Inhibiting LPS-induced iNOS	Reduce iNOS protein to 31.60 ± 2.15% at 10 μM	[60]
Ximaolide A	*Sarcophyton glaucum*	Inhibiting LPS-induced iNOS	Reduce COX-2 protein to 22.0 ± 6.5% at 20 μM	[58]
Casbane-type diterpenoid 1	*Lobophytum* sp.	Inhibiting the LPS/IFN-γ-induced NO release	IC_{50} = 41.21 μM	[61]
Cembrane diterpenoid 2	*Lobophytum* sp.	Inhibiting the LPS/IFN-γ-induced NO release	IC_{50} = 64.96 μM	[61]
Cembrane diterpenoid 3	*Lobophytum* sp.	Inhibiting the LPS/IFN-γ-induced NO release	IC_{50} = 74.76 μM	[61]
Klyflaccisteroid M	*Klyxum flaccidum*	Inhibition of elastase release in FMLP/CB-induced human neutrophils	79.24 ± 3.21% inhibition at 10 μM	[62]
Klyflaccisteroid K	*Klyxum flaccidum*	Inhibition of superoxide anion generation and elastase release in FMLP/CB-induced human neutrophils	67.03 ± 5.26% inhibition (superoxide anion) and 111.14 ± 2.47% inhibition (elastase) at 10 μM	[62]
Pinnisterol A	*Pinnigorgia* sp.	Inhibition of superoxide anion generation and	IC_{50} = 2.33 μM (superoxide anion) and IC_{50} = 3.32 μM	[63]

		elastase release in FMLP/CB-induced human neutrophils	(elastase)	
Pinnisterol C	*Pinnigorgia* sp.	Inhibition of superoxide anion generation and elastase release in FMLP/CB-induced human neutrophils	IC_{50} = 2.50 μM (superoxide anion) and IC_{50} = 2.81 μM (elastase)	[63]
Petasitosterone A	*Umbellulifera petasites*	Inhibiting LPS-induced NO release	Reduce NO level to 16.9% at 10 μg/mL	[64]
5*α*-pregna-1,20-dien-3-one	*Umbellulifera petasites*	Inhibiting LPS-induced NO release	Reduce NO level to 0.3% at 10 μg/mL	[64]
Nephthenol	*Lobophytum pauciflorum*	Inhibiting LPS-induced iNOS and COX-2	IC_{50} = 1.52 (iNOS) and 0.43 μM (COX-2)	[65]
Gorgost-5-ene-3*β*-ol	*Lobophytum pauciflorum*	Inhibiting LPS-induced iNOS and COX-2	IC_{50} = 1.0 (iNOS) and 0.29 μM (COX-2)	[65]
Briarenolide ZII	*Briareum* sp.	Inhibiting LPS-induced iNOS	Reduce iNOS protein to 47.2% at 10 μM	[66]
Briarenolide ZVI	*Briareum* sp.	Inhibiting LPS-induced iNOS	Reduce iNOS protein to 55.7% at 10 μM	[66]
Klyflaccisteroid J	*Klyxum flaccidum*	Inhibition of superoxide anion generation and elastase release in FMLP/CB-induced human neutrophils	76.35 ± 4.29% inhibition (superoxide anion) and 113.89 ± 2.99% inhibition (elastase) at 10 μM	[67]
(4*S**,5*S**)-4-hydroxy-5-(hydroxymethyl)-2,3-dimethyl-4-pentylcyclopent-2-en-1-one	*Sinularia verruca*	Inhibiting LPS-induced NO release	IC_{50} = 28 μM	[68]
4-hydroxy-4-(ethylpropanoate)-2,3-dimethyl-5-butylcyclopent-2-en-1-one	*Sinularia verruca*	Inhibiting LPS-induced NO release	IC_{50} = 24 μM	[68]
(4*S**,5*S**)-4-hydroxy-5-(ethoxymethyl)-2,3-dimethyl-4-pentylcyclopent-2-en-1-one	*Sinularia verruca*	Inhibiting LPS-induced NO release	IC_{50} = 26 μM	[68]
Locrassumin A	*Lobophytum crassum*	Inhibiting LPS-induced NO release	IC_{50} = 17μM	[69]
Locrassumin G	*Lobophytum crassum*	Inhibiting LPS-induced NO release	IC_{50} = 13μM	[69]
Sarcophytonolide O	*Lobophytum crassum*	Inhibiting LPS-induced NO release	IC_{50} = 8μM	[69]
ent-sarcophine	*Lobophytum crassum*	Inhibiting LPS-induced NO release	IC_{50} = 24μM	[69]
Ketoemblide	*Lobophytum crassum*	Inhibiting LPS-induced NO release	IC_{50} = 12μM	[69]
Nanoculone B	*Sinularia nanolobata*	Inhibiting LPS-induced NO release	Reduce NO level to 32.6% 100 μM	[70]
Nanolobol B	*Sinularia nanolobata*	Inhibiting LPS-induced NO release	Reduce NO level to 8.0% at 100 μM	[70]

Calyculone I	*Sinularia nanolobata*	Inhibiting LPS-induced NO release	Reduce NO level to 2.3% at 100 μM	[70]
Pinnigorgiol A	*Pinnigorgia* sp.	Inhibition of superoxide anion generation and elastase release in FMLP/CB-induced human neutrophils	IC_{50} = 4.0 μM (superoxide anion) and IC_{50} = 5.3 μM (elastase)	[63]
Pinnigorgiol B	*Pinnigorgia* sp.	Inhibition of superoxide anion generation and elastase release in FMLP/CB-induced human neutrophils	IC_{50} = 2.5 μM (superoxide anion) and IC_{50} = 3.1 μM (elastase)	[63]
Pinnigorgiol C	*Pinnigorgia* sp.	Inhibition of superoxide anion generation and elastase release in FMLP/CB-induced human neutrophils	IC_{50} = 2.7 μM (superoxide anion) and IC_{50} = 2.7 μM (elastase)	[63]
Sinubrasolide H	*Sinularia brassica*	Inhibition of elastase release in FMLP/CB-induced human neutrophils	32.4 ± 5.6% inhibition at 10 μM	[71]
Sarelengan B	*Sarcophyton elegans*	Inhibiting the LPS/IFN-γ-induced NO production	IC_{50} =18.2 μM	[72]
Sarelengan C	*Sarcophyton elegans*	Inhibiting the LPS/IFN-γ-induced NO production	IC_{50} = 32.5 μM	[72]
Isofuscol	*Lobophytum varium*	Inhibition of elastase release in FMLP/CB-induced human neutrophils	< 40% inhibition at 10 μM	[73]
(1*R*,2*R*,4*S*,17*R*)-loba-8,10,13(15)-trien-17,18-diol	*Lobophytum varium*	Inhibition of elastase release in FMLP/CB-induced human neutrophils	< 40% inhibition at 10 μM	[73]
Sinubrasolide J	*Sinularia brassica*	Inhibiting the superoxide anion generation in FMLP/CB-induced human neutrophils	32.1 ± 5.3% inhibition at 10 μM	[71]
Sinubrasolide K	*Sinularia brassica*	Inhibiting the superoxide anion generation in FMLP/CB-induced human neutrophils	34.3 ± 6.6% inhibition at 10 μM	[71]
Sinubrasolide L	*Sinularia brassica*	Inhibiting the superoxide anion generation and elastase release in FMLP/CB-induced human neutrophils	26.3 ± 0.7% inhibition (superoxide anion) and 25.0 ± 1.3% inhibition (elastase) at 10 μM	[71]
Sinubrasolide A	*Sinularia brassica*	Inhibiting the superoxide anion generation and elastase release in FMLP/CB-induced human neutrophils	87.7 ± 5.9% inhibition (superoxide anion) and 113.9 ± 1.8% inhibition (elastase) at 10 μM	[71]
Lobovarol E	*Lobophytum varium*	Inhibiting the superoxide anion generation and elastase release in FMLP/CB-induced human neutrophils	20.59 ± 2.15% inhibition (superoxide anion) and 23.07 ± 6.55% inhibition (elastase) at 10 μM	[73]
17,18-epoxyloba-8,10,13(15)-trien-16-ol	*Lobophytum varium*	Inhibiting the superoxide anion generation and elastase release in FMLP/CB-induced human neutrophils	28.16 ± 5.06% inhibition (superoxide anion) and 45.34 ± 4.08% inhibition (elastase) at 10 μM	[73]

Lobovarol B	*Lobophytum varium*	Inhibiting the superoxide anion generation in FMLP/CB-induced human neutrophils	22.08 ± 4.71% inhibition at 10 μM	[73]
11-*epi*-sinulariolide acetate	*Sinularia flexibilis*	Inhibiting LPS-induced TNF-*α*	IC_{50} = 2.7 μM	[74]
Sinulariolide	*Sinularia flexibilis*	Inhibiting LPS-induced TNF-*α*	IC_{50} = 4.7 μM	[74]
Flexilarin B	*Sinularia flexibilis*	Inhibiting LPS-induced TNF-*α* and NO	IC_{50} = 4.2 μM (TNF-*α*) and 28.5% inhibition (NO) at 25 μM	[9,74]
Xidaosinularide A	*Sinularia flexibilis*	Inhibiting LPS-induced TNF-*α*	IC_{50} = 38.9 μM	[74]
Sinuladiterpene I	*Sinularia flexibilis*	Inhibiting LPS-induced TNF-*α*	IC_{50} = 13.3 μM	[74]
Sinulaflexiolide L	*Sinularia flexibilis*	Inhibiting LPS-induced NO and TNF-*α*	33.50% inhibition (NO) and 41.50% inhibition (TNF-*α*) at 10 μM	[9]
Sinulaflexiolide M	*Sinularia flexibilis*	Inhibiting LPS-induced NO and TNF-*α*	33.00% inhibition (NO) and 29.20% inhibition (TNF-*α*) at 10 μM	[9]
Sinulaflexiolide N	*Sinularia flexibilis*	Inhibiting LPS-induced NO and TNF-*α*	20.40% inhibition (NO) and 40.00% inhibition (TNF-*α*) at 10 μM	[9]
Sinulaflexiolide O	*Sinularia flexibilis*	Inhibiting LPS-induced NO and TNF-*α*	16.50% inhibition (NO) and 53.90% inhibition (TNF-*α*) at 10 μM	[9]
ent-sinuflexibilin D	*Sinularia flexibilis*	Inhibiting LPS-induced NO and TNF-*α*	21.80% inhibition (NO) and 26.20% inhibition (TNF-*α*) at 10 μM	[9]
Dihydromanaarenolide I	*Sinularia flexibilis*	Inhibiting LPS-induced NO and TNF-*α*	61.2% inhibition (NO) and 63.0% inhibition (TNF-*α*) at 10 μM	[9]
(1*R*,13*S*,12*S*,9*S*,8*R*,5*S*,4*R*)-9-acetoxy5,8:12,13-diepoxycembr-15(17)-en-16,4-olide	*Sinularia flexibilis*	Inhibiting LPS-induced NO and TNF-*α*	22.3% inhibition (NO) and 28.4% inhibition (TNF-*α*) at 10 μM	[9]
Thioflexibilolide A	*Sinularia flexibilis*	Inhibiting LPS-induced NO and TNF-*α*	51.1% inhibition (NO) and 89.4% inhibition (TNF-*α*) at 10 μM	[9]
(−)14-Deoxycrassin	*Sinularia flexibilis*	Inhibiting LPS-induced NO and TNF-*α*	64.5% inhibition (NO) and 93.6% inhibition (TNF-*α*) at 10 μM	[9]
30-isoamylene butyrolactone IV	*Lobophytum michaelae*	Inhibiting LPS-induced NO production	nearly 25.3% inhibition at 20 μM	[75]
Butyrolactone I	*Lobophytum michaelae*	Inhibiting LPS-induced NO production	nearly 25.3% inhibition at 20 μM	[75]
Michosterol A	*Lobophytum michaelae*	Inhibiting superoxide anion generation and elastase release in FMLP/CB-induced human	IC_{50} = 7.1 ± 0.3 μM (superoxide anion) and IC_{50} = 4.5 ± 0.9 μM (elastase)	[76]

		neutrophils		
Michosterol B	*Lobophytum michaelae*	Inhibiting superoxide anion generation and elastase release in FMLP/CB-induced human neutrophils	IC_{50} = 7.1 ± 0.3 μM (superoxide anion) and IC_{50} = 4.5 ± 0.9 μM (elastase)	[76]
Michosterol C	*Lobophytum michaelae*	Inhibiting superoxide anion generation and elastase release in FMLP/CB-induced human neutrophils	IC_{50} > 10 μM (superoxide anion, elastase)	[76]
14-deoxycrassin	*Sinularia flexibilis*	Inhibiting superoxide anion generation and elastase release in FMLP/CB-induced human neutrophils	IC_{50} = 10.8 ± 0.38 (superoxide anion) and IC_{50} = 11.0 ± 1.52 μM (elastase) at 10 μM	[77]

Supplementary table S2. Cytotoxic compounds from coral

Compound	Coral species	Target cell lines	Activity value	Ref.
Hirsutalin A	*Cladiella hirsuta*	Hep3B, A549, Ca9-22	IC_{50} = 29, 28, 35 μM	[4]
Hirsutalin F	*Cladiella hirsuta*	HepG2, Hep3B, MCF-7	IC_{50} = 29, 29, 32 μM	[4]
Hirsutalin E	*Cladiella hirsuta*	Hep3B, MDA-MB-231, MCF-7, A549, Ca9-22	IC_{50} = 14, 41, 35, 34, 34 μM	[4]
3,4-epoxy-nephthenol acetate	*Nephthea* sp.	SF-268, MCF-7, H460	GI_{50} > 100 μM	[78]
Decaryiol	*Nephthea* sp.	SF-268, MCF-7, H460	GI_{50} > 100 μM	[78]
15-hydroxy-cembrenene	*Nephthea* sp.	SF-268, MCF-7, H460	GI_{50} > 100 μM	[78]
2-hydroxy-nephthenol	*Nephthea* sp.	SF-268, MCF-7, H460	GI_{50} > 100 μM	[78]
Nephthenol	*Nephthea* sp.	SF-268, MCF-7, H460	GI_{50} > 100 μM	[78]
Arachidonic acid	*Nephthea* sp.	SF-268, MCF-7, H460	GI_{50} > 100 μM	[78]
Lobophytene	*Lobophytum* sp.	A549, HT-29	IC_{50} = 8.2, 5.6 μM	[79]
(1*S*,2*S*,3*E*,7*E*,11*E*)-3,7,11,15-cembratetraen-17,2-olide	*Lobophytum* sp.	A549, HT-29	IC_{50} = 5.1, 1.8 μM	[79]
Diepoxycembrane A	*Sinularia facile*	HepG2	IC_{50} = 12.9 μg/mL	[80]
Lobatrienolide	*Lobophytum compactum*	A549, HL-60	IC_{50} = 23.03 ± 0.76, 24.79 ± 0.77 μM	[81]
3*β*,11-dihydroxy-24-methylene-9,11-secocholestan-5-en-9-one	*Lobophytum compactum*	A549, HL-60	IC_{50} of 4.97 ± 0.06, 17.80 ± 1.43 μM	[81]
(24*S*)-ergostane-3*β*,5*α*,6*β*,25-tetraol25-monoacetate	*Lobophytum compactum*	A549, HL-60	IC_{50} = 42.76 ± 2.85 μM	[81]
Lobocompactol A	*Lobophytum compactum*	HL-60	IC_{50} = 48.24 ± 1.33 μM	[81]
Lobocompactol B	*Lobophytum compactum*	HL-60	IC_{50} = 37.51 ± 0.38 μM	[81]
(24*S*)-ergostane-3*β*,5*α*,-6*β*,25-tetraol	*Lobophytum compactum*	HL-60	IC_{50} = 59.06 ± 2.31 μM	[81]
Lobatriene	*Lobophytum compactum*	A549, HL-60	IC_{50} = 31.13 ± 0.08, 33.82 ± 0.27 μM	[81]
Lobocrassin A	*Lobophytum crassum*	K562, CCRF-CEM, Molt4, HepG2, Huh7	IC_{50} = 15.39, 5.33, 11.86, 32.16, 26.13 μg/ml	[10]
Lobocrassin B	*Lobophytum crassum*	K562, CCRF-CEM, Molt4, HepG2, Huh7	IC_{50} = 2.97, 0.48, 0.34, 3.44, 8.17μg/ml	[10]
Lobocrassin C	*Lobophytum crassum*	K562, CCRF-CEM, Molt4, HepG2, Huh7	IC_{50} = > 40, 11.55, 9.51, > 40, 39.77μg/mL	[10]
Lobocrassin D	*Lobophytum crassum*	K562, CCRF-CEM, Molt4, HepG2, Huh7	IC_{50} = 24, 10.53, 10.99, 34.91, > 40 μg/ml	[10]
Paraminabeolide A	*Paraminabea acronocephala*	HepG2	IC_{50} = 8.0 μM	[13]

Minabeolide-1	*Paraminabea acronocephala*	HepG2, MCF-7	IC_{50} = 5.2, 18.7 μM	[13]
Paraminabeolide B	*Paraminabea acronocephala*	MDA-MB-231, MCF-7	IC_{50} = 19.3 and 14.9 μM	[13]
(1*R**,2*R**,3*R**,6*S**,7*S**,9*R**,10*R**,14*R**)-3-butanoyloxycladiell-11(17)-en-6,7-diol	*Cladiella krempfi*	A549, BT483, SAS	ED_{50} = 15.8 ± 2.0, 8.5 ± 1.0, 14.3 ± 1.8 μg/mL	[12]
Litophynol B	*Cladiella krempfi*	H1299, BT483	ED_{50} = 18.1 ± 1.5, 13.2 ± 1.1 μg/mL	[12]
13-acetoxysarcocrassolide	*Sarcophyton crassocaule*	BFTC	Reduce the population of BFTC nearly 50% and < 30% at 1.5 and 3 μg/mL	[82]
7*β*-acetoxy-8*α*-hydroxydeepoxysarcophine	*Sarcophyton glaucum*	HepG2, HCT-116, HeLa	IC_{50} = 3.6, 2.3, 6.7 μg/mL	[83]
(−)14-deoxycrassin	*Sinularia triangular*	CCRF-CEM, DLD-1	ED_{50} = 29.8, 32.2 μM	[8]
Sinularin	*Sinularia triangular*	CCRF-CEM, DLD-1	ED_{50} = 26.0, 37.1 μM	[8]
12*β*,16*β*,20-trihydroxycholesta-1,4-dien-3-one-16-acetate	*Sinularia* sp.	MCF-7, Bel-7402, HeLa	IC_{50} = 3.82, 14.47, 21.97 μg/mL	[84]
24-methyl-12*β*,16*β*,20-trihydroxycholesta-1,4-dien-3-one	*Sinularia* sp.	MCF-7, Bel-7402, HeLa	IC_{50} = 14.81, 45.05, 69.94 μg/mL	[84]
Culobophylin B	*Lobophytum crassum*	HL60, DLD-1, HCT-116	IC_{50} = 6.8, 16.2, 16.7 μg/mL	[85]
Culobophylin A	*Lobophytum crassum*	HL60, MDA-MB-231, DLD-1, HCT-116	IC_{50} = 3.0, 16.8, 4.6, 16.3 μg/mL	[85]
Capilloquinol	*Sinularia capillosa*	P-388	ED_{50} = 3.8 μg/mL	[86]
Emblide	*Lobophytum laevigatum*	HL-60	IC_{50} = 38.8 μM	[7]
(+)-sarcophine	*Lobophytum laevigatum*	HL-60	IC_{50} = 34.7 μM	[7]
Ximaolide F	*Lobophytum laevigatum*	HL-60, A549, HCT-116	IC_{50} = 9.0, 28.4, 16.4 μM	[87]
(22*S*,24*S*)-24-methyl-22,25-epoxyfurost-5-ene-3*β*,20*β*-diol	*Lobophytum laevigatum*	HCT-116	IC_{50} = 6.9 μM	[87]
(24*S*)-ergost-5-ene-3*β*,7*α*-diol	*Lobophytum laevigatum*	HCT-116	IC_{50} = 18.1 μM	[87]
Lobophytosterol	*Lobophytum laevigatum*	HCT-116, A549, HL-60	IC_{50} = 3.2, 4.5, 5.6 μM	[87]
Methyl tortuoate B	*Lobophytum laevigatum*	HL-60, A549, HCT-116	IC_{50} = 25.8, 24.4, 19.7 μM	[7]
Nyalolide	*Lobophytum laevigatum*	HL-60, A549, HCT-116, MCF-7	IC_{50} = 28.1, 28.7, 17.5, 35.5 μM	[7]
Sarcocrassocolide F	*Sarcophyton crassocaule*	Daoy, HEp-2, MCF-7, WiDr	ED_{50} = 7.3 ± 1.7, 15.0 ± 1.9, 19.4 ± 2.4, 18.4 ± 0.9 μM	[15]
Sarcocrassocolide G	*Sarcophyton crassocaule*	Daoy, HEp-2, MCF-7, WiDr	ED_{50} = 8.3 ± 1.4, 16.5 ± 1.7, 9.6 ± 2.7, 18.9 ± 1.9 μM	[15]
Sarcocrassocolide H	*Sarcophyton crassocaule*	Daoy, HEp-2, MCF-7, WiDr	ED_{50} = 6.4 ± 2.0, 13.5 ± 2.5, 9.4 ± 2.5, 18.7 ±	[15]

			1.0 μM	
Sarcocrassocolide I	*Sarcophyton crassocaule*	Daoy, HEp-2, MCF-7, WiDr	ED_{50} = 5.1 ± 1.2, 5.8 ± 0.5, 8.4 ± 1.5, 6.4 ± 2.0 μM	[15]
Sarcocrassocolide J	*Sarcophyton crassocaule*	Daoy, HEp-2, MCF-7, WiDr	ED_{50} > 20 μM	[15]
Sarcocrassocolide K	*Sarcophyton crassocaule*	Daoy, HEp-2, MCF-7, WiDr	ED_{50} = 9.9 ± 4.0, >20, 10.2 ± 1.0, > 20 μM	[15]
Sarcocrassocolide L	*Sarcophyton crassocaule*	Daoy, HEp-2, MCF-7, WiDr	ED_{50} >20 μM	[15]
Asterolaurin L	*Asterospicularia Laurae*	HEp-2, Daoy, MCF-7, WiDr	ED_{50} = 4.12, 6.23, 4.09, 6.08 μg/mL	[88]
Gorgosten-5(*E*')-3*β*-ol	*Heteroxenia ghardaqensis*	Caco-2	IC_{50} = 379.6 μg/mL	[89]
Gorgostan-3*β*,5*α*,6*β*,11*α*-tetraol	*Heteroxenia ghardaqensis*	Caco-2	IC_{50} = 2170.8 μg/mL	[89]
Crassarosterol A	*Sinularia crassa*	HepG2	IC_{50} = 14.9 μM	[20]
Crassarosteroside C	*Sinularia crassa*	HepG2, HepG3	IC_{50} = 17.6 and 18.9 μM	[20]
Echinoclerodane A	*Echinomuricea* sp.	MOLT-4, HL-60, DLD-1, LoVo	IC_{50} = 13.18, 14.89, 23.44, 21.69 μM	[24]
3*β*,11-dihydroxy-5*β*,6*β*-expoxy-24-methylene-9,11-secocholestan-9-one	Sinularia granosa	Daoy, MCF-7	ED_{50} = 7.07 ± 0.71, 9.98 ± 0.32 μg/mL	[23]
8*α*H-3*β*,11-dihydroxy-5*α*,6*α*-expoxy-24-methylene-9,11-secocholestan-9-one	*Sinularia granosa*	HeLa, HEp 2, Daoy, MCF-7	ED_{50} = 8.21 ± 1.61, 6.21 ± 1.38, 5.53 ± 1.58, 4.99 ± 0.70 μg/mL	[23]
Sinularone C	*Sinularia* sp.	A2780, A549, BGC823, Bel7402, HCT-8	IC_{50} > 10 μg/mL	[90]
Sinularone A	*Sinularia* sp.	A2780, A549, BGC823, Bel7402, HCT-8	IC_{50} > 10 μg/mL	[90]
Sinularone B	*Sinularia* sp.	A2780, A549, BGC823, Bel7402, HCT-8	IC_{50} > 10 μg/mL	[90]
Sinularone D	*Sinularia* sp.	A2780, A549, BGC823, Bel7402, HCT-8	IC_{50} > 10 μg/mL	[90]
Sinularone E	*Sinularia* sp.	A2780, A549, BGC823, Bel7402, HCT-8	IC_{50} > 10 μg/mL	[90]
Sinularone F	*Sinularia* sp.	A2780, A549, BGC823, Bel7402, HCT-8	IC_{50} > 10 μg/mL	[90]
Sinularone G	*Sinularia* sp.	A2780, A549, BGC823, Bel7402, HCT-8	IC_{50} > 10 μg/mL	[90]
Sinularone H	*Sinularia* sp.	A2780, A549, BGC823, Bel7402, HCT-8	IC_{50} > 10 μg/mL	[90]
Sinularone I	Sinularia sp.	A2780, A549, BGC823, Bel7402, HCT-8	IC_{50} > 10 μg/mL	[90]
Butenolide	*Sinularia* sp.	A2780, A549, BGC823, Bel7402, HCT-8	IC_{50} > 10 μg/mL	[90]
Ceratosteroid C	*Scleronephthya gracillimum*	HepG2, Hep3B, Ca9-22	IC_{50} = 46.1, 48.8, 44.3 μM	[22]
Ceratosteroid D	*Scleronephthya gracillimum*	HepG2, Hep3B, Ca9-22, A-549, MCF-7	IC_{50} = 36.8, 35.3, 34.7, 46.2, 42.2 μM	[22]
Stereonsteroid E	*Scleronephthya gracillimum*	Ca9-22	IC_{50} = 36.6 μM	[22]
Sclerosteroid H	*Scleronephthya gracillimum*	Hep3B, Ca9-22	IC_{50} = 36.3, 37.9 μM	[22]

Stereosteroid F	*Scleronephthya gracillimum*	Hep3B, Ca9-22	IC_{50} = 32.2, 37.3 μM	[22]
Sclerosteroid A	*Scleronephthya gracillimum*	HepG2 and MDA-MB-23	IC_{50} = 19.5 and 15.8 μM	[22]
Stereonsteroid A	*Scleronephthya gracillimum*	HepG2, Hep3B, Ca9-22	IC_{50} = 45.7, 44.4, 39.3 μM	[22]
Pregnene glycoside	*Scleronephthya gracillimum*	HepG2, Hep3B, Ca9-22	IC_{50} = 32.1, 30.3, 28.4 μM	[22]
Sclerosteroid I	*Scleronephthya gracillimum*	HepG2, Hep3B, Ca9-22, A-549	IC_{50} = 37.4. 31.5, 30.8, 28.9 μM	[22]
Sclerosteroid F	*Scleronephthya gracillimum*	HepG2, Hep3B, Ca9-22, A-549, MCF-7, MDA-MB-231	IC_{50} = 35.6, 29.3, 28.9, 30.6, 38.7, 30.8 μM	[22]
Sclerosteroid G	*Scleronephthya gracillimum*	HepG2, Hep3B, Ca9-22, A-549, MCF-7, MDA-MB-231	IC_{50} = 29.0, 28.1, 27.2, 28.7, 34.1, 28.0 μM	[22]
Stereonsteroid H	*Scleronephthya gracillimum*	HepG2, Hep3B, Ca9-22, A-549, MCF-7, MDA-MB-231	IC_{50} = 28.4, 28.9, 26.9, 29.7, 34.1, 27.8 μM	[22]
Stereonsteroid D	*Scleronephthya gracillimum*	HepG2, Hep3B, Ca9-22, A-549, MCF-7, MDA-MB-231	IC_{50} = 23.5, 26.8, 24.7, 28.9, 31.0, 27.0 μM	[22]
Stereonsteroid G	*Scleronephthya gracillimum*	HepG2, Hep3B, Ca9-22, A-549, MDA-MB-231	IC_{50} = 33.2, 31.2, 28.1, 30.8, 32.0 μM	[22]
Sclerosteroid B	*Scleronephthya gracillimum*	HepG2, MDA-MB-231	IC_{50} = 35.0, 41.3 μM	[22]
Scabralin A	*Sinularia scabra*	MCF-7, WiDr, Daoy, HEpG2	ED_{50} = 9.6, 10.7, 7.6, 13.8 μg/mL	[17]
12(*S*)-hydroperoxylsarcoph-10-ene	*Sarcophyton glaucum*	Inhibiting phase I enzyme cytochrome P_{450} 1A	IC_{50} = 2.7 nM	[91]
8-*epi*-sarcophinone	*Sarcophyton glaucum*	Inhibiting phase I enzyme cytochrome P_{450} 1A	IC_{50} = 3.7 nM	[91]
ent-sarcophine	*Sarcophyton glaucum*	Inhibiting phase I enzyme cytochrome P_{450} 1A	IC_{50} = 3.4 nM	[91]
Alcyonolide	*Cespitularia* sp.	HCT-116	IC_{50} = 5.85 μM	[92]
Five new diterpenoids	*Cespitularia* sp.	HCT-116	IC_{50} = 28.18 to 91.35 μM	[92]
24-methylenecholestane-3*β*,5*α*,6*β*-triol-6-monoacetate	*Sinularia* sp.	Artemia salina, K562	LC_{50} = 0.96 μM, IC_{50} = 3.18 μM	[93]
Michaolide L	*Lobophytum michaelae*	A-549, HT-29, P-388, HEL	ED_{50} = 1.2, 0.8, 0.3, 1.0 μM	[94]
Michaolide M	*Lobophytum michaelae*	A-549, HT-29, P-388, HEL	ED_{50} = 2.0, 4.9, 1.5, 3.2 μM	[94]
Michaolide N	*Lobophytum michaelae*	A-549, HT-29, P-388, HEL	ED_{50} = 2.1, 1.6, 0.4, 2.0 μM	[94]
Michaolide P	*Lobophytum michaelae*	A-549, HT-29, P-388, HEL	ED_{50} = 2.0, 1.5, 1.0, 1.8 μM	[94]
Michaolide Q	*Lobophytum michaelae*	A-549, HT-29, P-388, HEL	ED_{50} = 1.9, 1.4, 0.4, 1.7 μM	[94]
(+)-12-ethoxycarbonyl-11*Z*-sarcophine	*Sarcophyton ehrenbergi*	A549, P-388	IC_{50} = 20.8, 5.8 μg/mL	[95]
Ehrenbergol B	*Sarcophyton ehrenbergi*	A549, P-388	IC_{50} = 10.2, 4.7 μg/mL	[95]
Ehrenbergol A	*Sarcophyton ehrenbergi*	P-388	IC_{50} = 7.4μg/mL	[95]

Nebrosteroid N	*Nephthea chabrolii*	P-388	ED_{50} = 0.9 μg/mL	[96]
Nebrosteroid O	*Nephthea chabrolii*	P-388	ED_{50} = 1.2 μg/mL	[96]
Nebrosteroid P	*Nephthea chabrolii*	P-388	ED_{50} = 1.7 μg/mL	[96]
Sarcophytoxide	*Sarcophyton* sp.	HepG2, Hep3B, MDA-MB-231, MCF-7, A549, Ca9-22	IC_{50} = 16.2, 12.4, 13.2, 13.2, 15.3, 18.9 μg/mL	[97]
Cyclolobatriene	*Lobophytum pauciflorum*	A431	IC_{50} = 0.64 μM	[98]
Eunicol	*Lobophytum pauciflorum*	A431	IC_{50} = 0.35 μM	[98]
Fuscol	*Lobophytum pauciflorum*	A431	IC_{50} = 0.52 μM	[98]
Lobatriene	*Lobophytum pauciflorum*	A431	IC_{50} = 0.41 μM	[98]
3*β*-hexadecanoylcholest-5-en-7*β*-one	*Antipathes dichotoma*	HepG2, WI 38, VERO, MCF-7	IC_{50} = 28.7, 30.5, 28.8, 29.1 μg/mL	[99]
3*β*-hexadecanoylcholest-5-en-7*β*-ol	*Antipathes dichotoma*	HepG2, WI 38, VERO, MCF-7	IC_{50} = 81.2, 82.6, 82.3, 78.2 μg/mL	[99]
Cholest-5-en-3*β*-yl-formate	*Antipathes dichotoma*	HepG2, WI 38, VERO, MCF-7	IC_{50} = 38.1, 31.1, 39.6, 37.4 μg/mL	[99]
Thymidine	*Antipathes dichotoma*	HepG2, WI 38, VERO, MCF-7	IC_{50} = 45.1, 48.2, 20.5, 20.2 μg/mL	[99]
Indole-3-carboxaldehyde	*Antipathes dichotoma*	HepG2, WI 38, VERO, MCF-7	IC_{50} = 70.5, 84.8, 72.4, 77.5 μg/mL	[99]
Sarcocrassocolide M	*Sarcophyton crassocaule*	Daoy, HEp-2, MCF-7, WiDr	ED_{50} = 6.6 ± 0.8, 10.4 ±1.1, 10.6 ± 0.5, >40 μM	[21]
Sarcocrassocolide N	*Sarcophyton crassocaule*	Daoy, HEp-2, MCF-7, WiDr	ED_{50} = 5.2 ± 0.6, 12.3 ± 1.6, 10.1 ± 2.3, 30.1 ± 2.8 μM	[21]
Sarcocrassocolide O	*Sarcophyton crassocaule*	Daoy, HEp-2, MCF-7, WiDr	ED_{50} = 5.0 ± 0.7, 12.4 ± 2.1, 6.4 ± 0.5, >40 μM	[21]
5-episinuleptolide acetate	*Sinularia* sp.	K562, MOLT-4, HTC-11, DLD-1, T-47D, MDA-MB-231	IC_{50} = 0.67, 0.59, 4.09, 0.92, 3.09, 2.95 μg/mL	[100]
Paraminabic acid C	*Paraminabea acronocephala*	Hep3B, MDA-MB-231, MCF-7, A-549	IC_{50}= 2.83, 2.25, 2.23, 2.05 μg/mL	[34]
Krempfielin I	*Cladiella krempfi*	A549, BT483, H1299, HepG2, SAS	ED_{50} = 15.0 ± 3.5, 11.5 ± 1.8, 19.2 ± 4.0, 12.9 ± 3.1, 10.2 ± 3.5 μg/mL	[28]
Litophynin F	*Cladiella krempfi*	A549, BT483, H1299, HepG2, SAS, BEAS2B	ED_{50} = 12.2 ± 1.1, 6.8 ± 0.6, 12.8 ± 1.2, 11.1 ± 0.4, 10.3 ± 0.5, 13.6 ± 0.5 μg/mL	[28]
6-acetoxy litophynin E	*Cladiella krempfi*	A549, BT483, H1299, SAS, BEAS2B	ED_{50} = 6.8 ± 1.0, 11.6 ± 2.8, 6.7 ± 0.7, 8.5 ± 1.3, 9.5 ± 3.7, 4.8 ± 0.7 μg/mL	[28]
6-methyl ether of litophynol B	*Cladiella krempfi*	A549, BT483, H1299, SAS, BEAS2B	ED_{50} = 16.1 ± 1.2, 10.0 ± 1.8, 11.8 ± 1.0, 17.2 ± 0.4, 10.4 ± 0.3 μg/mL	[28]
11-acetylsinuflexolide	*Sinularia flexibilis*	HeLa, HEp-2, MCF-7, MDA-MB-231	IC_{50} = 9.5, 11.3, 17.8, 15.7 μg/mL	[101]
Sinuflexolide	*Sinularia flexibilis*	HeLa, HEp-2, MCF-7, MDA-MB-231	IC_{50} = 8.6, 8.2, 16.0, 11.3 μg/mL	[101]

Sinularin	*Sinularia flexibilis*	HEp-2, MCF-7, MDA-MB-231	IC_{50} = 12.6, 17.5, 13.5 μg/mL	[101]
Sinubrasolide B	*Sinularia brassica*	P388, MOLT 4, HT-29	ED_{50} = 9.1 ± 1.4, 4.8 ± 0.9, 4.8 ± 0.7 Mm	[102]
Sinubrasolide A	*Sinularia brassica*	K562	ED_{50} = 8.7 ± 1.4 μM	[102]
Sinubrasolide E	*Sinularia brassica*	MOLT-4, HT-29	ED_{50} = 9.9 ± 1.8, 7.5 ± 1.5 μM	[102]
Durumolide C	*Sinularia polydactyla*	HepG2	IC_{50} = 1.0 μg/mL	[103]
24-methylcholestane-3*β*,5*α*,6*β*,25-tetrol 25-monoacetate	*Sinularia polydactyla*	HepG2, HCT-116	IC_{50} = 6.1, 8.2 μg/mL	[103]
Sclerosteroid M	*Scleronephthya gracillimum*	HepG2, A549, MDA-MB-231	IC_{50} = 23.3, 21.9, 24.3 μM	[31]
Nephthoacetal	*Nephthea* sp.	HeLa	IC_{50} = 12.3 μg/mL	[104]
(18*S*)-18-*O*-acetyl-nephthoacetal	*Nephthea* sp.	HeLa	IC_{50} = 10.1 μg/mL	[104]
(18*R*)-18-*O*-acetyl-nephthoacetal	*Nephthea* sp.	HeLa	IC_{50} = 19.6 μg/mL	[104]
(12b,22*R*)-12-acetoxy-22-hydroxy-cholesta-1,4-dien-3-one	*Nephthea* sp.	HeLa	IC_{50} = 7.51 ± 0.22 μg/mL	[104]
(12b,22*R*)-12-hydroxy-22-acetoxy-cholesta-1,4-dien-3-one	Nephthea sp.	HeLa	IC_{50} = 7.50 ± 0.31 μg/mL	[104]
(12b, 22R)-12, 22-diacetoxy-cholesta-1,4-dien-3-one	*Nephthea* sp.	HeLa	IC_{50} = 18.48 ± 0.56 μg/mL	[104]
(22*R*)-18, 22-diacetoxy-cholesta-1,4dien-3-one	*Nephthea* sp.	HeLa	IC_{50} = 8.29 ± 0.42 μg/mL	[104]
(20*R*,22*R*)-20-hydroxy-22-acetoxy-cholesta-1,4-dien-3-one	*Nephthea* sp.	HeLa	IC_{50} = 17.25 ± 0.61 μg/mL	[104]
Astrogorgol N	*Nephthea* sp.	HeLa	IC_{50} = 18.72 ± 0.78 μg/mL	[104]
(24*R*)-gorgost-25-en-3*β*,5*α*,6*β*,11*α*-tetraol	*Sarcophyton* sp.	K562	IC_{50} = 9.9 μM	[105]
(24*S*)-23,24-dimethylcholest-22-en-3*β*,5*α*,6*β*,11*α*-tetraol	*Sarcophyton* sp.	K562	IC_{50} = 26.6 μg/mL	[105]
11*α*-acetoxycholest-24-en-1*α*,3*β*,5*α*,6*β*-tetraol	*Sarcophyton* sp.	K562, HL-60	IC_{50} = 10.1, 17.2 μM	[105]
11*α*-acetoxy-cholest-24-en-3*β*,5*α*,6*β*-triol	*Sarcophyton* sp.	K562, HL-60	IC 50 = 9.1, 14.3 μM	[105]
(24*S*)-11*α*-acetoxy-ergost-3*β*,5*α*,6*β*-triol	*Sarcophyton* sp.	K562, HL-60	IC_{50} = 10.3, 12.8 μM	[105]
(24*S*)-ergost-3*β*,5*α*,6*β*,11*α*-tetraol	*Sarcophyton* sp.	K562, HL-60	IC_{50} = 24.5, 32.5 μM	[105]
(23*R*,24*R*,17*Z*)-11*α*-acetoxy-16*β*-methoxy-23,24-dimethylcholest-17(20)-en-	*Sarcophyton* sp.	K562, HL-60, HeLa	IC_{50} = 17.3, 9.3, 17.0 μM	[105]

3β,5α,6β-triol				
(22*E*,24*S*)-11α-acetoxy-ergost-22, 25-dien-3β,5α,6β-triol	*Sarcophyton* sp.	K562, HL-60, HeLa	IC_{50} = 6.4, 10.5, 11.5 μM	[105]
(24*R*)-11α-acetoxy-gorgost-3β,5α,6β-triol	*Sarcophyton* sp.	K562, HL-60, HeLa	IC_{50} = 9.8, 14.6, 24.7 μM	[105]
(1*R**,3*R**,4*R**,14*R**,7*E*,11*E*)-3,4-epoxycembra-7,11,15(17)-trien-16,14-olide	*Lobophytum* sp.	SGC7901, A549, MCF7, HCT116, B16	IC_{50} = 5.3, 6.1, 3.8, 5.2, 8.6 μg/ml	[106]
(1*R**,7*S**,14*S**,3*E*,11*E*)-7-hydroperoxycembra-3,8(19),11,15(17)-tetraen-16,14-olide	*Lobophytum* sp.	SGC7901, A549, MCF7, HCT116, B16	IC_{50} = 2.7, 3.2, 1.2, 4.5, 2.1 μg/ml	[106]
(1*R**,7*S**,14*S**,3*E*,11*E*)-18-acetoxy-7-hydroperoxycembra-3,8(19),11,15(17)-tetraen-16,14-olide	*Lobophytum* sp.	SGC7901, A549, MCF7, HCT116, B16	IC_{50} = 2.3, 1.8, 2.9, 3.4, 5.6 μg/ml	[106]
Klymollin M	*Klyxum molle*	K562, MOLT-4, T47D	ED_{50} = 7.97 ± 2.55, 4.35 ± 0.63, 8.58 ± 1.72 μM	[37]
24-methylcholesta-5,24-(28)-diene-3β,7β,19-triol	*Litophyton arboreum*	HeLa, U937	IC_{50} = 8 ± 0.5, 16.4 ± 1.25 μM	[107]
Sarcophytol M	*Litophyton arboreum*	HeLa	IC_{50} = 27.5 ± 0.2 μM	[107]
7β-acetoxy-24-methylcholesta-5-24(28)-diene-3,19-diol	*Litophyton arboreum*	HeLa, U937	IC_{50} = 5.3 ± 0.60, 10.6 ± 0.12 μM	[107]
(4-(benzo[d][1,3]dioxol-5-ylmethyl)piperazin-1-yl)(5-((1*E*,5*Z*)-2,6-dimethylocta-1,5,7-trienyl)furan-3-yl)methanone	*Sinularia kavarattiensis*	THP1, DU145	IC_{50} = 15.9, 17.9 μM	[108]
(4-benzhydrylpiperazin-1-yl)(5-((1*E*,5*Z*)-2,6-dimethylocta-1,5,7-trienyl)furan-3-yl)methanone	*Sinularia kavarattiensis*	THP1, DU145	IC_{50} = 17.5, 16.8 μM	[108]
Leptoclalin A	*Sinularia leptoclados*	T47D, K-562	IC_{50} = 15.4, 12.8 μg/mL	[109]
(22*R*,23*R*,24*R*)-5α,8α-epidioxy-22,23-methylene-24-methylcholest-6-en-3β-ol	*Sinularia gaweli*	HL-60	IC_{50} = 12.14 μg/ml	[110]
24-methylenecholestane-1α,3β,5α,6β,11α-pentol	*Sinularia gaweli*	K562, MOLT-4, HL-60	IC_{50} = 9.71, 6.91, 3.39 μg/ml	[110]
(22*R*,23*R*,24*R*)-5α,8α-epidioxy-22,23-methylene-24-methylcholest-6,9(11)-dien-3β-ol	*Sinularia gaweli*	MOLT-4	IC_{50} = 15.7 μg/ml	[110]

22α-acetoxy-24-methylene-3β,6α,11-trihydroxy-9,11-seco-cholest-7-en-9-one	*Sinularia nanolobata*	P-388	IC_{50} = 10.2 μg/mL	[111]
11-acetoxy-24-methylene-1β,3β,6α-trihydroxy-9,11-seco-cholest-7-en-9-one	*Sinularia nanolobata*	P-388	IC_{50} = 27.8 μg/mL	[111]
5-*epi*-sinuleptolide	*Sinularia nanolobata*	P-388	IC_{50} = 15.7 μg/mL	[111]
Litophynin F	*Cladiella krempfi*	C6	IC_{50} = 46 ± 3 μM	[112]
Litophynol A	*Cladiella krempfi*	C6	IC_{50} = 70-80 μM	[112]
Litophynol A acetate	*Cladiella krempfi*	C6	IC_{50} = 21 ± 2 μM	[112]
Litophynol B	*Cladiella krempfi*	C6	IC_{50} = 70-80 μM	[112]
Philippinlin A	*Lemnalia philippinensis*	HepG2, MDA-MB231, A549	IC_{50} = 16.0, 16.3, 15.8 μg/mL	[113]
7-keto-8α-hydroxy-deepoxysarcophine	*Sarcophyton ehrenbergi*	MCF-7	IC_{50} = 192.87 μmol/mL	[114]
7β-chloro-8α-hydroxy-12-acetoxy-deepoxysarcophine	*Sarcophyton ehrenbergi*	MCF-7	IC_{50} = 68.57 μmol/mL	[114]
(*E*)-methyl-3-(5-butyl-1-hydroxy-2,3-dimethyl-4-oxocyclopent-2-enyl)acrylate	*Sarcophyton ehrenbergi*	MCF-7	IC_{50} = 114.41 μmol/mL	[114]
5α-pregna-1,20-dien-3-one	*Scleronephthya flexilis*	MOLT-4, HL-60, K-562	IC_{50} = 2.15, 3.14, 8.32 μg/mL	[115]
Dendronephthol A	*Dendronephthya* sp.	L5187Y	ED_{50} = 8.4 μg/mL	[116]
Dendronephthol C	*Dendronephthya* sp.	L5187Y	ED_{50} = 6.8 μg/mL	[116]
Klymollin W	*Klyxum molle*	CCRF-CEM, Molt-4, T47D	ED_{50} = 9.6, 8.5, 19.9 μg/mL	[51]
Klymollin X	*Klyxum molle*	CCRF-CEM, K562, Molt-4, T47D, DLD-1	ED_{50} = 4.2, 15.0, 16.5, 12.4 μg/mL	[51]
Palustrol	*Sarcophyton trocheliophorum*	Lymphoma and Erlish	LC_{50} = 2.8, 3.11 μM	[117]
Sarcophine	*Sarcophyton trocheliophorum*	Lymphoma and Erlish	LC_{50} = 2.5, 3.79 μM	[117]
Sinulariaoid A	Sinularia sp.	HepG2/ADM	IC_{50} = 9.70 ± 1.77 μM	[118]
Sarcophine	*Sarcophyton auritum*	HepG2 and and MCF-7	IC_{50} = 23 ± 0.12, 22.4 ± 0.22 μg/mL	[119]
2-*epi*-sarcophine	*Sarcophyton auritum*	HepG2 and and MCF-7	IC_{50} = 20.6 ± 0.31, 19.7 ± 0.24 μg/ml	[119]
(+)-7α,8β-dihydroxydeepoxysarcophine	*Sarcophyton auritum*	HepG2, MCF-7	IC50= 11 ± 0.22, 18.4 ± 0.16 μg/mL	[119]
(1*R*,2*E*,4*S*,6*E*,8*R*,11*R*,12*R*)-2,6-cembradiene-4,8,11,12-tetrol	*Sarcophyton auritum*	HepG2, MCF-7	IC_{50} = 21.1 ± 0.16, 20 ± 0.12 μg/mL	[119]
Trisnorditerpenoid	*Cespitularia* sp.	HCT116	IC_{50} = 6.04 μM	[120]
Diterpenoid	*Cespitularia* sp.	HCT116	IC_{50} = 47.0 μM	[120]
Kelsoenethiol	*Nephthea erecta*	P-388, HT-29	ED_{50} = 1.3, 1.8 μg/mL	[121]

Sinugyrosanolide A	*Sinularia gyrosa*	P-388	EC_{50} = 11.8 μM	[122]
Sarcophytolol	*Sarcophyton glaucum*	HepG2	IC_{50} = 20 μM	[123]
Sarcophytolide C	*Sarcophyton glaucum*	HepG2, MCF-7	IC_{50} = 20, 29 μM	[123]
10(14)-aromadendrene	*Sarcophyton glaucum*	HepG2, PC-3	IC_{50} = 20, 9.3 μM	[123]
Sarcophytolide B	*Sarcophyton glaucum*	MCF-7	IC_{50} = 25 μM	[123]
Hirsutalin E	*Cladiella hirsuta*	A549	IC_{50} = 37.2 μM	[50]
Hirsutalin R	*Cladiella hirsuta*	P388, K562	IC_{50} = 13.8, 36.3 μM	[50]
Crassocolide A	*Sarcophyton crassocaule*	DLD-1, CCRF-CEM	ED_{50} = 5.7, 6.3μM	[49]
Sarcocrassocolide R	*Sarcophyton crassocaule*	DLD-1, CCRF-CEM, HL-60	ED_{50} = 10.0, 28.1, 8.7μM	[49]
Crassocolide B	*Sarcophyton crassocaule*	DLD-1, CCRF-CEM, HL-60	ED_{50} = 3.8, 8.7, 7.3μM	[49]
Crassocolide E	*Sarcophyton crassocaule*	DLD-1, CCRF-CEM, HL-60	ED_{50} = 7.9, 11.1, 8.4μM	[49]
Sarcocrassocolide P	*Sarcophyton crassocaule*	DLD-1, CCRF-CEM, HL-60	ED_{50} = 21.8, 48.8, 24.9μM	[49]
Sarcocrassocolide Q	*Sarcophyton crassocaule*	DLD-1, CCRF-CEM, HL-60	ED_{50} = 35.8, 73.1, 18.6μM	[49]
Crassocolide D	*Sarcophyton crassocaule*	DLD-1, CCRF-CEM, HL-60	ED_{50} = 27.7, 41.9, 34.6μM	[49]
Gibberoketosterol	*Sinularia numerosa*	P-388	ED_{50} = 6.9 μM	[124]
Cespitulone A	*Cespitularia taeniata*	Daoy, WiDr	IC_{50} = 8.7 and 6.7 μM	[125]
Klyflaccisteroid A	*Klyxum flaccidum*	A549	ED_{50} = 7.7 μg/mL	[57]
Klyflaccisteroid C	*Klyxum flaccidum*	HT-29, A549	ED_{50} = 8.2, 6.1 μg/mL	[57]
Klyflaccisteroid E	*Klyxum flaccidum*	HT-29, P388	ED_{50} = 6.9, 3.7 μg/mL	[57]
Hirsutosteroside B	*Cladiella hirsuta*	K562, P388, HT-29	IC_{50} = 39.3, 10.2, 29.1 μM	[55]
Hirsutosteroside A	*Cladiella hirsuta*	K562, A549	IC_{50} = 27.6, 32.2 μM	[55]
5*β*,6*β*-epoxy-3*β*,11-dihydroxy-24-methylene9,11-secocholestan-9-one	*Sinularia nanolobata*	K562, P388, HT-29	IC_{50} = 15.8, 15.5, 12.6 μM	[55]
Cladophenol glycoside A	*Cladiella hirsuta*	K562, P388, HT-29	IC_{50} = 18.5, 21.1, 20.3 μM	[55]
Sinulerectol C	*Sinularia erecta*	K-562	IC_{50} = 9.2 ± 3.3 μM	[56]
Glaucumolide A	*Sarcophyton glaucum*	HL-60, CCRF-CEM	ED_{50} = 6.6 ± 1.2, 7.4 ± 1.5 μg/mL	[58]
Sinulerectadione	*Sinularia erecta*	K-562, MOLT-4	IC_{50} = 8.6 ± 1.1, 9.7 ± 2.9 μM	[56]
Glaucumolide B	*Sarcophyton glaucum*	HL-60, CCRF-CEM	ED_{50} = 3.8 ± 0.9, 5.3 ± 1.4 μg/mL	[58]
(*Z*)-*N*-[2-(4-hydroxyphenyl)ethyl]-3-	*Sinularia erecta*	CCRF-CEM, MOLT-4	IC_{50} = 6.3 ± 1.5, 9.7 ± 3.6 μM	[56]

methyldodec-2-enamide				
3*α*,6*α*-epidioxyhimachal-1-ene	*Litophyton arboreum*	MCF-7, HCT116, HepG-2	IC_{50} = 19.1 ± 0.032 to 1000 μM	[126]
22-norerogostane derivative,13,14-seco-22-norergosta-4,24(28)-dien-19-hydroperoxide-3-one	*Litophyton arboreum*	MCF-7, HCT116, HepG-2	IC50 from 19.1 ± 0.032 to 1000 μM	[126]
11-acetoxy-15,17-dihydroxy-2,12-epoxy-(3*E*,7*E*)-1-cembra-3,7-diene	*Litophyton arboreum*	MCF-7, HCT116, HepG-2	IC_{50} = 19.1 ± 0.032, 22.0 ± 0.092, 24.0 ± 0.032 μM	[126]
Erythro-*N*-dodecanoyl-docosasphinga-(4*E*,8*E*)-dienine	*Litophyton arboreum*	MCF-7, HCT116, HepG-2	IC_{50} = 19.1 ± 0.032 to 1000 μM	[126]
Sarcophytol M	*Litophyton arboreum*	MCF-7, HCT116, HepG-2	IC_{50} = 19.1 ± 0.032 to 1000 μM	[126]
7*β*-acetoxy-24-methylcholesta-5-24(28)-diene-3,19-diol	*Litophyton arboreum*	MCF-7, HCT116, HepG-2	IC_{50} = 19.1 ± 0.032 to 1000 μM	[126]
(3*β*,5*α*,6*β*,22*E*)-3,5-dihydroxy-24-oxocholest-22-en-6-yl acetate	*Sinularia acuta*	HeLa	IC_{50} = 44.8 μM	[127]
(3*β*,5*α*,6*β*)-3,5-dihydroxyergost-24-ene-6,28-diyl diacetate	*Sinularia acuta*	HL-60, HeLa	IC_{50} = 7.3, 27.1 μM	[127]
24-methylidenecholestane-3*β*,5*α*,6*β*-triol 6-monoacetate	*Sinularia acuta*	HL-60, K562	IC_{50} = 9.9, 10.9 μM	[127]
(24*S*)-methylidenecholestane-3*β*,5*α*,6i-triol 6-monoacetate	*Sinularia acuta*	K562, HeLa	IC_{50} = 11.7, 18.2 μM	[127]
Methyl tortuoate B	*Sarcophyton pauciplicatum*	HepG2, HL-60, KB, LNCaP, SK-Mel2, and SW480	IC_{50} = 11.60 ± 0.30 to 30.03 ± 5.61 μM	[128]
Sarcophytolide M	*Sarcophyton pauciplicatum*	HepG2, HL-60, KB, LNCaP, LU-1, MCF7, SK-Mel2, SW480	IC_{50} = 15.31 ± 0.80 to 44.01 ± 1.51 μM	[128]
Sarcophytolide L	*Sarcophyton pauciplicatum*	HepG2, HL-60, KB, LNCaP, LU-1, MCF7, SK-Mel2, SW480	IC_{50} = 47.39 ± 1.03 to 67.29 ± 2.91 μM	[128]
Sarcophytolide I	*Sarcophyton pauciplicatum*	HepG2, HL-60, KB, LNCaP, LU-1, MCF7, SK-Mel2, SW480	IC_{50} = 12.34 ± 0.17 to 37.87 ± 3.82 μM	[128]
Sarcosarcophytolide J	*Sarcophyton pauciplicatum*	HepG2, HL-60, KB, LNCaP, LU-1, MCF7, SK-Mel2, SW480	IC_{50} = 10.54 ± 0.33 to 34.35 ± 1.61 μM	[128]
Methyl sartortuoate	*Sarcophyton pauciplicatum*	HepG2, HL-60, KB, LNCaP, LU-1, MCF7, SK-Mel2, SW480	IC_{50} = 7.93 ± 2.08 to 19.34 ± 0.72 μM	[128]
Lobophytone U	*Sarcophyton pauciplicatum*	HepG2, HL-60, KB, LNCaP, SK-Mel2, SW480	IC_{50} =16.22 ± 0.22 to 94.18 ± 3.02 μM	[128]
Ehrenbergol D	*Sarcophyton ehrenbergi*	P-388	EC_{50} = 2.0 μM	[129]

Ehrenbergol E	*Sarcophyton ehrenbergi*	P-388	EC_{50} = 3.0 μM	[129]
Columnariol A	*Nephthea columnaris*	LNCaP	IC_{50} = 9.80 μg/mL	[59]
Flexibilide	*Sinularia flexibilis*	Anti-tumor activity targeting the inositol-requiring 1/X-box-binding protein 1 (IRE1 /XBP1) signaling pathway	IC_{50} = 4.10 μg/mL	[130]
Klysimplexin Q	*Klyxum simplex*	HepG2, Hep3B, MDA-MB-231, MCF-7, A549, Ca9-22	IC_{50} = 53.2, 35.1, 44.0, 36.5, 40.5, 40.5 μM	[130]
Klysimplexin T	*Klyxum simplex*	HepG2, Hep3B, MDA-MB-231, MCF-7, A549, Ca9-22	IC_{50} = 34.3, 26.4, 44.0, 27.2, 42.0, 37.4 μM	[130]
Petasitosterone B	*Umbellulifera petasites*	DLD-1	IC_{50} = 6.4 ± 1.4 μg/mL	[64]
Petasitosterone C	*Umbellulifera petasites*	DLD-1	IC_{50} = 15.2 ± 3.5 μg/mL	[64]
5*α*-pregna-1,20-dien-3-one	*Umbellulifera petasites*	K-562, MOLT-4, DLD-1	IC_{50} = 13.5 ± 3.1, 5.9 ± 1.9, 9.7 ± 3.2 μg/mL	[64]
Petasitosterone A	*Umbellulifera petasites*	MOLT-4, DLD-1	IC_{50} = 12.1 ± 4.5, 5.8 ± 1.7 μg/mL	[64]
Cembrene A	*Lobophytum* sp.	A. Salina and Erhlich	LD_{50} = 25, 50 μg/mL	[131]
Nebrosteroid-*M*-[4*α*,24-dimethyl-5*α*-cholest-24(28)-en3*β*,8*β*,11*β*-triol]	*Litophyton mollis*	A549	IC_{50} = 20.4 ± 1.1 μM	[132]
4*α*,24-dimethyl-5*α*-cholest-24(28)-en-3*β*,8*β*,18-triol	*Litophyton mollis*	K562, A549	IC_{50} = 8.9 ± 0.9, 25.7 ± 1.5 μM	[132]
(22*E*,24*R*)-4*α*,24-dimethyl-5*α*-cholest-22-en-3*β*,8*β*,11*β*-triol	*Litophyton mollis*	K562, A549	IC_{50} = 7.7 ± 0.8, 20.8 ± 1.2 μM	[132]
Nebrosteroid-*D*-[(22*E*)-4*α*,24-dimethyl-5*α*-cholesta-22,24(28)-dien-3*β*,8*β*,11*β*-triol]	*Litophyton mollis*	K562, A549	IC_{50} = 6.0 ± 0.5, 22.1 ± 1.4 μM	[132]
Nebrosteroid-*A*-[23-oxo-4*α*,24-dimethyl-5*α*-cholest-24(28)-en3*β*,8*β*,11*β*-triol]	*Litophyton mollis*	K562	IC_{50} = 5.8 ± 0.8 μM	[132]
23n-acetoxy-4*α*,24-dimethyl-5*α*-cholest-24(28)-en-3*β*,8*β*,11btriol	*Litophyton mollis*	K562	IC_{50} = 5.6 ± 1.2 μM	[132]
Suberosoid	*Subergorgia suberosa*	HeLa	IC_{50} = 10.6 μM	[133]
Sarcophytosterol	*Sinularia nanolobata*	HL-60	IC_{50} = 89.02 ± 9.93 μM	[134]
24(*S*),28-epoxyergost-5-ene-3*β*,4*α*-diol	*Sinularia nanolobata*	HL-60, HepG2, SW480	IC_{50} = 33.53 ± 4.25, 64.35 ± 7.00, 71.02 ± 4.00 μM	[134]
Casbane-type diterpenoid 1	*Lobophytum* sp.	HCT116	IC_{50} = 135.57 μM	[61]
Cembrane diterpenoid 2	*Lobophytum* sp.	HCT116	IC_{50} = 177.11 μM	[61]

Cembrane diterpenoid 3	*Lobophytum* sp.	HCT116	IC_{50} = 153.11 μM	[61]
24-methyl-cholesta-5,24(28)-diene3*β*,19-diol-7*β*-monoacetate	*Nephthea erecta*	K562, Molt-4, Sup-T1	IC_{50} = 11.2, 19.9, 16.3 μM	[135]
Erectasteroid F	*Nephthea erecta*	K562, Molt-4, Sup-T1, U937	IC_{50} = 6.5, 8.0, 8.0, 12.9 μM	[135]
(3*β*,7*β*)-ergost-5,24(28)-diene-3*β*,7*β*,19-triol-7,19-diacetate	*Nephthea erecta*	K562, Molt-4, Sup-T1, U937	IC_{50} = 14.0, 7.9, 7.3, 6.8 μM	[135]
Klyflaccisteroid J	*Klyxum flaccidum*	HT-29, P388, K562	IC_{50} = 15.1, 14.8, 12.7 μM	[67]
Klyflaccisteroid H	*Klyxum flaccidum*	P388	IC_{50} = 15.5 μM	[67]
Klyflaccisteroid K	*Klyxum flaccidum*	HT-29, K562, A549, DLD-1	IC_{50} = 27.5, 22.5, 15.8, 27.5 μM	[62]
Klyflaccisteroid L	*Klyxum flaccidum*	HT-29, P388	IC_{50} = 36.7, 31.8 μM	[62]
Pinnisterol A	*Pinnigorgia* sp.	HSC-T6	46.5% inhibition at 10 μM	[63]
Pinnigorgiol A	*Pinnigorgia* sp.	HSC-T6	IC_{50} = 5.77 ± 0.27 μM	[63]
Pinnigorgiol B	*Pinnigorgia* sp.	HSC-T6	IC_{50} = 7.89 ± 0.52 μM	[63]
Bisdioxycalamenene	*Rhytisma fulvum*	Artemia salina	LD_{50} = 15 μg/mL	[136]
Protoxenicin A	*Protodendron repens*	A-549, HT-29, MDA-MB-231	GI_{50} = 2.1, 0.6, 1.1 μM	[137]
Protoxenicin B	*Protodendron repens*	A-549, HT-29, MDA-MB-231	GI_{50} = 6.3, 1.7, 6.1 μM	[137]
Sarcoaldesterol B	*Sarcophyton glaucum*	HepG2, MDA-MB-231, A-549	IC_{50} = 9.7, 14.0, 15.8 μg/mL	[138]
Sarcomilasterol	*Sarcophyton glaucum*	MDA-MB-231, MOLT-4, SUP-T, U-937	IC_{50} = 13.8, 6.7, 10.5, 17.7 μg/mL	[138]
Sinubrasolide H	*Sinularia brassica*	P388, MOLT-4, K-562, HT-29	IC_{50} = 39.8, 28.6, 29.7, 24.4 μg/mL	[71]
Sinubrasolide J	*Sinularia brassica*	P388, MOLT-4, K-562, HT-29	IC_{50} = 18.7, 17.2, 12.6, 11.2 μg/mL	[71]
Sinubrasolide K	*Sinularia brassica*	P388, MOLT-4, K-562, HT-29	IC_{50} = 18.3, 13.7, 17.4, 20.5 μg/mL	[71]
Sinubrasolide A	*Sinularia brassica*	P388, MOLT-4, K-562, HT-29	IC_{50} = 29.9, 12.1, 8.7, 18.7 μg/mL	[71]
(1*R*,2*E*,4*R*,6*R*,7*E*,10*S*,11*S*,12*R*)-10,18-diacetoxydolabella-2,7-dien-6-one	*Clavularia viridis*	A549, MCF-7	IC_{50} = 10.5 and 12.6 μM	[139]
Ergost-24(28)-ene-3,5,6-triol,(3*β*,5*α*,6*β*)-triol	*Sinularia terspilli*	HL60, K562	IC_{50} = 0.004, 0.005 μM	[140]
Ergost-24(28)-ene-1,3,6,11-tetraacetyl-5-ol, (1*α*,3*β*,5*α*,6*β*,11*α*)	*Sinularia terspilli*	HL60, K563	IC_{50} = 0.002, 0.003 μM	[140]
Alismol	*Sinularia terspilli*	HL60, K564	IC_{50} = 0.30, 0.35 μM	[140]
1*S*,4*S*,5*S*,10*R*-4,10-guaianediol	*Sinularia terspilli*	HL60, K565	IC_{50} = 0.21, 0.35 μm	[140]
Clavirolide G	*Clavularia viridis*	KB, HL-60	IC_{50} = 15.4, 17.8 μmol/L	[141]

14-deoxycrassin	*Sinularia flexibilis*	P-388, K-562	IC_{50} = 16.0, 26.7 μM	[77]
Dehydrosinulariolide	*Sinularia flexibilis*	P-388, K-562, HT-29	IC_{50} = 9.3, 23.4, 15.9 μM	[77]
Sinulariolide	*Sinularia flexibilis*	K-562, HT-29	IC_{50} = 21.7, 27.1 μM	[77]
11-*epi*-sinulariolide acetate	*Sinularia flexibilis*	P-388, K-562, HT-29	IC_{50} = 6.9, 12.2, 9.6 μM	[77]
Michosterol A	*Lobophytum michaelae*	A549	IC_{50} = 14.9 ± 5.7 μg/mL	[76]
Sinulariolide	*Sinularia flexibilis*	HT-29, SNU-398, Capan-1	IC_{50} = 33.6, 24.7, 26.1μM	[74]
5-dehydrosinulariolide	*Sinularia flexibilis*	A549, HT-29, SNU-398, Capan-1	IC_{50} = 27.4, 22.7, 8.9, 9.4 μM	[74]
11-*epi*-sinulariolide acetate	*Sinularia flexibilis*	HT-29, SNU-398, Capan-1	IC_{50} = 32.6, 24.9, 28.7μM	[74]

Supplementary table S3. Antimicrobial compounds from coral

Compound	Coral species	Reference microorganisms	Activity value	Ref.
Sinularoside A	*Sinularia humilis* Ofwegen	*M. violaceum, S. tritici, B. megaterium, C. fusca*	D = 17, 14, 30, 12 mm	[142]
Sinularoside B	*Sinularia humilis* Ofwegen	*M. violaceum, S. tritici, B. megaterium, C. fusca*	D = 17, 15, 30, 14 mm	[142]
(3*β*,5*α*,6*β*)-ergost-24(28)-en-3,5,6,19-tetrol 19-monoacetate	*Sinularia depressa*	*S. aureus*	MIC_{50} = 15.6 µM	[143]
Sarcophytolide	*Sarcophyton trocheliophorum* Marenzeller	*S. aureus*	MIC = 125 µg/mL	[144]
Sarcophine	*Sarcophyton glaucum*	*C. neoformans*	IC_{50} = 20 µg/mL	[145]
(22*E*,24*S*)-11*α*-acetoxy-ergostane-22,25-dien-3*β*,5*α*,6*β*-triol	*Sarcophyton* sp.	*E. coli, B. megaterium, M. violaceum, S. tritici*	D = 14.5, 12, 10, 7.5 mm	[146]
(24*S*)-11*α*-acetoxy-ergostane-3*β*,5*α*,6*β*-triol	*Sarcophyton* sp.	*E. coli, B. megaterium, M. violaceum, S. tritici*	D = 7.0, 7.5, 6.0, 6.5 mm	[146]
(24*S*)-23,24-dimethylcholesta-22-en-3*β*,5*α*,6*β*,11*α*-tetraol	*Sarcophyton* sp.	*E. coli, B. megaterium, M. violaceum, S. tritici*	D = 6.0, 7.0, 7.5, 9.0 mm	[146]
(24*S*)-ergostane-1*α*,3*β*,5*α*,6*β*,11*α*-pentaol	*Sarcophyton* sp.	*E. coli, B. megaterium, M. violaceum, S. tritici*	D = 7.5, 4.5, 7.0, 10.5 mm	[146]
(24*S*)-ergostane-3*β*,5*α*,6*β*,11*α*-tetraol	*Sarcophyton* sp.	*E. coli, B. megaterium, M. violaceum, S. tritici*	D = 6.0, 8.5, 8.5, 4.5 mm	[146]
(24*S*)-ergostane-3*β*,5*α*,6*β*-triol	*Sarcophyton* sp.	*E. coli, B. megaterium, M. violaceum, S. tritici*	D = 4.5, 4.5, 7.0, 6.5 mm	[146]
(24*S*)-ergostane-7-en-3*β*,5*α*,6*β*-triol	*Sarcophyton* sp.	*E. coli, B. megaterium, M. violaceum, S. tritici*	D = 6.0, 7.0, 6.0, 6.0 mm	[146]
11*α*-acetoxy-cholesta-24-en-3*β*,5*α*,6*β*-triol	*Sarcophyton* sp.	*E. coli, B. megaterium, M. violaceum, S. tritici*	D = 12, 10, 6.5, 10.5 mm	[146]
11*α*-acetoxy-gorgostane-3*β*,5*α*,6*β*,12*α*-tetraol	*Sarcophyton* sp.	*E. coli, B. megaterium, M. violaceum, S. tritici*	D = 9.5, 10, 8.5, 10 mm	[146]
11*α*-acetoxy-gorgostane-3*β*,5*α*,6*β*-triol	*Sarcophyton* sp.	*E. coli, B. megaterium, M. violaceum, S. tritici*	D = 7.0, 8.0, 9.5, 10 mm	[146]
12*α*-acetoxy-gorgostane-3*β*,5*α*,6*β*,11*α*-tetraol	*Sarcophyton* sp.	*E. coli, B. megaterium, M. violaceum, S. tritici*	D = 8.0, 10, 7.0, 4.5 mm	[146]
Gorgostane-1*α*,3*β*,5*α*,6*β*,11*α*-pentaol	*Sarcophyton* sp.	*E. coli, B. megaterium, M. violaceum, S. tritici*	D = 5.0, 4.0, 8.5, 7.0 mm	[146]

Gorgostane-3*β*,5*α*,6*β*,11*α*-tetraol	*Sarcophyton* sp.	*E. coli, B. megaterium, M. violaceum, S. tritici*	D = 10, 6.5, 11.5, 7.5 mm	[146]
1-(11′-hydroxymethyl-hexadecanoyl)-2-pentadecanoyl-3-propanol	*Heteroxenia ghardaqensis*	*M. luteus, K. pneumonia, C. albicans*	MIC = 64, 125, 125 μg/disc	[147]
Cembrene-C	*Sarcophyton trocheliophorum*	*S. aureus, Acinetobacter spp., MRSA, A. flavus, C. albicans*	MIC ≥ 13.6, ≥ 13.6, ≥ 13.6, 0.68, 0.68 μM	[117]
Palustrol	*Sarcophyton trocheliophorum*	*S. aureus, Acinetobacter spp., MRSA, A. flavus, C. albicans*	MIC = 6.6, 6.6, 11.1, ≥ 11.1, ≥ 11.1 μM	[117]
Sarcophine	*Sarcophyton trocheliophorum*	*S. aureus, Acinetobacter spp., MRSA, A. flavus, C. albicans*	MIC = 9.4, 9.4, 9.4, ≥ 21.3, ≥ 21.3 μM	[117]
Sarcotrocheliol	*Sarcophyton trocheliophorum*	*S. aureus, Acinetobacter spp., MRSA, A. flavus, C. albicans*	MIC = 1.53, 3.06, 3.06, ≥ 15.3, ≥ 15.3 μM	[117]
Sarcotrocheliol acetate	*Sarcophyton trocheliophorum*	*S. aureus, Acinetobacter spp., MRSA, A. flavus, C. albicans*	MIC = 1.74, 4.34, 4.34, ≥17.4, ≥17.4 μM	[117]
10*α*-methoxy-4*β*-hydroxy guaine-6-ene	*Sinularia kavarattiensis*	*S. aureus*	MIC = 37.5 μg/mL	[148]
New sesquiterpene	*Sinularia kavarattiensis*	*S. aureus*	MIC = 18.75 μg/mL	[148]
1*S**,4*S**,5*S**,10*R**-4,10-guaianediol	*Sinularia kavarattiensis*	*S. epidermidis*	MIC = 18.75 μg/mL	[148]
Subergosterone B	*Subergorgia rubra*	*B. cereus*	MIC = 1.56 μM	[149]
Subergosterone C	*Subergorgia rubra*	*B. cereus*	MIC = 1.56 μM	[149]
Gersemiol A	*Gersemia fruticosa*	*MRSA*	50% inhibition at 48 μg/mL	[150]
Eunicellol A	*Gersemia fruticosa*	*MRSA*	MIC_{90} = 24–48 μg/mL	[150]
Capgermacrene D	*Capnella imbricata*	*S. aureus and MRSA*	MIC = 200 μg/mL	[151]
Capgermacrene E	*Capnella imbricata*	*S. aureus and MRSA*	MIC = 180, 240 μg/mL	[151]
Capgermacrene F	*Capnella imbricata*	*S. aureus and MRSA*	MIC = 150 and 125 μg/mL	[151]
Capgermacrene G	*Capnella imbricata*	*S. aureus and MRSA*	MIC = 125 and 175 μg/mL	[151]
Stearic acid	*Lobophytum pauciflorum*	*B. subtilis*	MIC 50 μg/mL	[65]
Batilol	*Lobophytum pauciflorum*	*B.subtilis, S. lutea and C. albicans*	MIC = 25, 25, 50 μg/mL	[65]
Nephthenol	*Lobophytum pauciflorum*	*C. albicans*	MIC = 50 μg/mL	[65]
Gorgost-5-ene-3*β*-ol	*Lobophytum pauciflorum*	*C. albicans*	MIC = 50 μg/mL	[65]
Heptadecan-1-ol	*Lobophytum pauciflorum*	*P. aeruginosa, S. aureus, B. subtilis, M. pheli, S. lutea, C. albicans*	MIC = 50, 50, 50, 25, 50, 50 μg/mL	[65]

Alismol	*Lobophytum* sp.	*S.aureus, S. epidermis, S. pneumonia, P. aeruginosa*	MIC = 15-30 μg/mL, D = 12-18 mm	[131]
Alismoxide	*Lobophytum* sp.	*S.aureus, S. epidermis, S. pneumonia, P. aeruginosa*	MIC = 30 μg/mL, D = 11 - 15 mm	[131]
Aristol-9-ene	*Lobophytum* sp.	*S.aureus, S. epidermis, S. pneumonia, P. aeruginosa*	MIC ≥ 30 μg/mL, D = 7 - 10 mm	[131]
Cembrene A	*Lobophytum* sp.	*S.aureus, S. epidermis, S. pneumonia, P. aeruginosa*	MIC = 30 μg/mL, D = 11 - 15 mm	[131]
Chalinasterol	*Lobophytum* sp.	*S.aureus, S. epidermis, S. pneumonia, P. aeruginosa*	MIC ≥ 30 μg/mL, D = 7 - 10 mm	[131]
Nardol	*Lobophytum* sp.	*S.aureus, S. epidermis, S. pneumonia, P. aeruginosa*	MIC ≥ 30 μg/mL, D = 7 - 9 mm	[131]
Nephalsterol C	*Lobophytum* sp.	*S.aureus, S. epidermis, S. pneumonia, P. aeruginosa*	MIC = 30 μg/mL, D = 7 - 9 mm	[131]
Sarcotrocheldiol A	*Sarcophyton trocheliophorum*	*K. pneumonia, S. aureus, S. epidermidis*	D = 2 - 8 mm	[152]
Sarcotrocheldiol B	*Sarcophyton trocheliophorum*	*A. baumannii, E. coli, K. pneumonia, P. aeruginosa*	D = 6 - 11 mm	[152]
Trocheliane	*Sarcophyton trocheliophorum*	*A. baumannii, E. coli, K. pneumonia, P. aeruginosa, S. aureus, S. epidermidis, S. pneumoniae*	MIC = 4 - 6 μM, D = 12-18 mm	[152]
Casbane-type diterpenoid 1	*Lobophytum* sp.	*S. aureus, E. coli*	D = 10 mm	[61]
Cembrane diterpenoids 2	*Lobophytum* sp.	*S. aureus, S. enterica, E. coli*	D = 9 - 12 mm	[61]
Cembrane diterpenoids 3	*Lobophytum* sp.	*S. aureus, S. enterica, E. coli*	D = 9 - 10 mm	[61]
Nephthenol	*Nephthea* sp.	*L. thermophilum*	MIC = 12.5 μg/mL	[153]
Nephthecrassocolide A	*Nephthea* sp.	*L. thermophilum*	MIC = 12.5 μg/mL	[153]

Supplementary table S4. Antiviral compounds from coral

Compound	Coral species	Target virus	Activity value	Ref.
(+)-12-ethoxycarbonyl-11*Z*-sarcophine	*Sarcophyton ehrenbergi*	Human cytomegalovirus (HCMV)	IC_{50} = 60 µg/mL	[95]
Ehrenbergol A	*Sarcophyton ehrenbergi*	HCMV	IC_{50} = 46 µg/mL	[95]
Ehrenbergol B	*Sarcophyton ehrenbergi*	HCMV	IC_{50} = 5.0 µg/mL	[95]
(24*R*)-gorgost-25-en-3β,5α,6β,11α-tetraol	*Sarcophyton* sp.	H1N1 (Influenza A virus)	IC_{50}= 19.6 µg/mL	[105]
(24*S*)-ergost-3β,5α,6β,11α-tetraol	*Sarcophyton* sp.	H1N1 (Influenza A virus)	IC_{50}= 36.7 µg/mL	[105]
Sinuleptolide	*Sinularia nanolobata*	HCMV	ED_{50} = 1.92 µg/mL	[111]
Ehrenbergol C	*Sarcophyton ehrenbergi*	HCMV	EC_{50} = 20 µg/mL	[154]
Acetyl ehrenberoxide B	*Sarcophyton ehrenbergi*	HCMV	EC_{50} = 8.0 µg/mL	[154]
Alismol	*Litophyton arboretum*	HIV-1 PR enzymes	IC_{50} = 7.20 ± 0.7 µM	[107]
7β-acetoxy-24-methylcholesta-5-24(28)-diene-3,19-diol	*Litophyton arboretum*	HIV-1 PR enzymes	IC_{50} = 4.85 ± 0.18 µM	[107]
Erythro-*N*-dodecanoyl-docosasphinga-(4*E*,8*E*)-dienine	*Litophyton arboretum*	HIV-1 PR enzymes	IC_{50} = 4.80 ± 0.92 µM	[107]
Secocrassumol	*Lobophytum crissum*	HCMV	IC_{50} = 5.0 µg/mL	[155]
(+)-2-*epi*-12-methoxycarbonyl-11*E*-sarcophine	*Sarcophyton ehrenbergi*	HCMV	IC_{50} = 25.0 µg/mL	[129]
(4*S**,5*S**)-4-hydroxy-5-(hydroxymethyl)-2,3-dimethyl-4-pentylcyclopent-2-en-1-one	*Sinularia verruca*	HIV-1 virus	EC_{50} = 34 µM	[68]
(*S*)-4-hydroxy-5-methylene-2,3-dimethyl-4-pentylcyclopent-2-en-1-one	*Sinularia verruca*	HIV-1 virus	EC_{50} = 5.8 µM	[68]
(4*S**,5*S**)-4-hydroxy-5-(ethoxymethyl)-2,3-dimethyl-4-pentylcyclopent-2-en-1-one	*Sinularia verruca*	HIV-1 virus	EC_{50} = 30 µM	[68]
Subergorgol T	*Subergorgia suberosa*	A/WSN/33 (H1N1)	IC_{50} = 35.64 µM	[156]
Subergorgol U	*Subergorgia suberosa*	A/WSN/33 (H1N1)	IC_{50} = 37.73 µM	[156]
(10)-trien-20-one	*Subergorgia suberosa*	A/WSN/33 (H1N1)	IC_{50} = 50.95 µM	[156]
1,2-dehydroprogesterone	*Subergorgia suberosa*	A/WSN/33 (H1N1)	IC_{50} = 41.6 µM	[156]

Supplementary table S5. Antifouling compounds from coral

Compound	Coral species	Fouling organisms	Activity value	Ref.
Sinularone A	*Sinularia* sp.	Larvae of the barnacle *Balanus amphitrite*	EC_{50} = 13.86 μg/mL	[90]
Sinularone B	*Sinularia* sp.	Larvae of the barnacle *Balanus amphitrite*	EC_{50} = 23.50 μg/mL	[90]
Sinularone G	*Sinularia* sp.	Larvae of the barnacle *Balanus amphitrite*	EC_{50} = 18.65 μg/mL	[90]
Sinularone H	*Sinularia* sp.	Larvae of the barnacle *Balanus amphitrite*	EC_{50} = 21.39 μg/mL	[90]
Sinularone I	*Sinularia* sp.	Larvae of the barnacle *Balanus amphitrite*	EC_{50} = 12.58 μg/mL	[90]
Butenolide	*Sinularia* sp.	Larvae of the barnacle *Balanus amphitrite*	EC_{50} = 3.84 μg/mL	[90]
Capillosanane I	*Sinularia capillosa*	The barnacle *Balanus amphitrite*	IC_{50} = 5.40 μM	[27]
Capillosanane A	*Sinularia capillosa*	The barnacle *Balanus amphitrite*	IC_{50} = 9.70 μM	[27]
Sinulariol Z	*Sinularia rigida*	The barnacle *Balanus amphitrite* and *Bugula neritina*	EC_{50} = 4.57 and 13.48 μg/mL	[157]
(2*E*,7*E*)-4,11-dihydroxy1,12-oxidocembra- 2,7-diene	*Sinularia rigida*	The barnacle *Balanus amphitrite* and *Bugula neritina*	EC_{50} = 4.86 and 12.34 μg/mL	[157]
Nephthoacetal	*Nephthea* sp.	The larvae *Bugula neritina*	EC_{50} = 2.5 μg/mL	[104]
16,22-epoxy-20*β*,23*S*-dihydroxycholest-1-ene-3-one	*Subergorgia suberosa*	Larvae of the barnacle *Balanus amphitrite*	EC_{50} = 5.3 μg/mL	[158]
(1*R*,13*S*,12*S*,9*S*,8*R*,5*S*,4*R*)-9-acetoxy-5,8:12,13-diepoxycembr-15(17)-en-16,4-olide	*Sinularia flexibilis*	The larvae of the barnacle *Balanus albicostatus*	EC_{50} = 20.34 μg/mL	[159]
11-dehydrosinulariolide	*Sinularia flexibilis*	The larvae of the bryozoan *Bugula neritina*	EC_{50} = 21.02 μg/mL	[159]
Dihydrosinularin	*Sinularia flexibilis*	The larvae of the bryozoan *Bugula neritina*	EC_{50} = 55.60 μg/mL	[159]
(−)14-deoxycrassin	*Sinularia flexibilis*	The larvae of the bryozoan *Bugula neritina* and the barnacle *Balanus albicostatus*	EC_{50} = 3.90 and 21.26 μg/mL	[159]
Epoxycembrane A	*Sinularia flexibilis*	The larvae of the bryozoan *Bugula neritina* and the barnacle *Balanus albicostatus*	EC_{50} = 21.37 and 30.60 μg/mL	[159]
Sinulariolide	*Sinularia flexibilis*	The larvae of the bryozoan *Bugula neritina* and the barnacle *Balanus albicostatus*	EC_{50} = 33.18 and 21.00 μg/mL	[159]

Supplementary table S6. Other bioactive compounds from coral

Compound	Coral species	Activity type	Activity value	Ref
Crassumolide E	*Lobophytum* sp.	Acetylcholinesterase inhibitory	At least 1 μg as AChE inhibitor	[160]
(22*S*,24*S*)-24-methyl-22,25-epoxyfurost-5-ene-3*β*,20*β*-diol	*Lobophytum laevigatum*	PPARs transcriptional activity	2.0-fold activation at 10 μM	[87]
(24*S*)-ergost-5-ene-3*β*,7*α*-diol	*Lobophytum laevigatum*	PPARs transcriptional activity	2.5-fold activation 10 μM	[87]
Pregnenolone	*Lobophytum laevigatum*	PPARs transcriptional activity	2.1-fold activation 10 μM	[87]
Ethyl acetate fraction	*Lobophytum* sp.	Heme polymerization inhibitory	IC_{50} = 11.7 μg/mL	[161]
n-butanol fraction	*Lobophytum* sp.	Heme polymerization inhibitory	IC_{50} = 14.3 μg/mL	[161]
Aqueous fraction	*Lobophytum* sp.	Heme polymerization inhibitory	IC_{50} = 12.0 μg/mL	[161]
(3*β*,4*α*,5*α*,8*β*)-4-methylergost-24(28)-ene-3,8-diol	*Sinularia depressa*	PTP1B inhibitory	IC_{50} = 22.7 μM	[143]
(3*β*,4*α*,5*α*)-4-methylergost-24(28)-ene-3-ol	*Sinularia depressa*	PTP1B inhibitory	IC_{50} = 19.5 μM	[143]
Ergost-4,24(28)-diene-3-one	*Sinularia depressa*	PTP1B inhibitory	IC_{50} = 15.3 μM	[143]
Sarsolilide A	*Sarcophyton trocheliophorum*	PTP1B inhibitory	IC_{50} = 6.8 ± 0.96 μM	[162]
Sarsolilide B	*Sarcophyton trocheliophorum*	PTP1B inhibitory	IC_{50} = 27.1 ± 2.6 μM	[162]
An unnamed prenyleudesmane diterpene	*Sinularia polydactyla*	PTP1B inhibitory	IC_{50} = 75.5 μM	[163]
Sinupol	*Sinularia polydactyla*	PTP1B inhibitory	IC_{50} = 63.9 μM	[163]
Sinulacetate	*Sinularia polydactyla*	PTP1B inhibitory	IC_{50} = 51.8 μM	[163]
Montiporic acid D	*Montipora digitata*	DPPH radical scavenging activity	35% inhibition at 1 mg/mL	[164]
(*Z*)-13,15-hexadecadien-2,4-diyn-1-ol	*Montipora digitata*	DPPH radical scavenging activity	25% inhibition at 1 mg/mL	[164]
n-butanol fraction	*Lobophytum* sp.	DPPH radical scavenging activity	IC_{50} = 150 μg/mL	[161]

Supplementary table S7. Anti-inflammatory and cytotoxic compounds from coral-associated microorganisms

Compound	Coral species	Microorganisms	Target cell lines/ anti-inflammatory type	Activity value	Ref.
Versicolactone B	*Lobophytum michaelae*	*Aspergillus terreus*	Inhibition of NO production	Nearly 60% inhibition at 20 mM	[165]
3′-isoamylene butyrolactone IV	*Lobophytum michaelae*	*Aspergillus terreus*	Inhibition of NO production	Nearly 25.1% inhibition at 20 mM	[165]
Butyrolactone I	*Lobophytum michaelae*	*Aspergillus terreus*	Inhibition of NO production	Nearly 25.3% inhibition at 20 mM	[165]
Altersolanol C	*Sarcophyton* sp.	*Alternaria* sp.	HCT-116, MCF-7/ADR, PC-3, HepG2 and Hep3B	IC_{50} = 2.2, 3.2, 7.6, 8.9, 8.2 μM	[166]
Alterporriol P	*Sarcophyton* sp.	*Alternaria* sp.	HCT-116, MCF-7/ADR, PC-3, HepG2 and Hep3B	IC_{50} = 8.6, 23, 6.4, 20, 21 μM	[166]
Alterporriol C	*Sarcophyton* sp.	*Alternaria* sp.	HCT-116, MCF-7/ADR, PC-3, HepG2 and Hep3B	IC_{50} = 24, 98, 27, 53, 51 μM	[166]
Chondrosterin A	*Sarcophyton tortuosum*	*Chondrostereum* sp.	A549, CNE2, LoVo	IC_{50} = 2.45, 4.95, 5.47 μM	[167]
Strepchloritide A	Unidentified Coral	*Streptomyces* sp.	MCF-7	IC_{50} = 9.9 μmol/L	[168]
Strepchloritide B	Unidentified Coral	*Strepomyces* sp.	MCF-7	IC_{50} = 20.2 μmol/L	[168]
Aspetritone A	*Galaxea fascicularis*	*Aspergillus tritici*	HeLa, A549, HepG2	IC_{50} = 2.67 ± 0.60, 3.13 ± 0.68, 3.87 ± 0.74 μM	[169]
3-prenylterphenyllin	*Galaxea fascicularis*	*Aspergillus tritici*	HeLa, A549, HepG2	IC_{50} = 3.23 ± 0.40, 3.87 ± 0.15, 2.10 ± 0.20 μM	[169]
Aspetritone B	*Galaxea fascicularis*	*Aspergillus tritici*	HeLa, A549, HepG2	IC_{50} = 10.57 ± 0.93, 4.67 ± 0.60, 8.57 ± 0.83 μM	[169]
3,4-dimethyl-3″-prenylcandidusin A	*Galaxea fascicularis*	*Aspergillus tritici*	HeLa, A549, HepG2	IC_{50} = 16.77 ± 0.45, 21.07 ± 0.76, 27.17 ± 0.29 μM	[169]
4-methyl-3″-prenylcandidusin A	*Galaxea fascicularis*	*Aspergillus tritici*	HeLa, A549, HepG2	IC_{50} = 10.20 ± 0.50, 13.07 ± 0.72, 35.10 ± 1.00 μM	[169]
Candidusin A	*Galaxea fascicularis*	*Aspergillus tritici*	HeLa, A549, HepG2	IC_{50} = 25.07 ±0.81, 19.07 ± 0.64, 32.10 ± 2.00 μM	[169]
Terphenyllin	*Galaxea fascicularis*	*Aspergillus tritici*	HeLa, A549, HepG2	IC_{50} = 18.87 ± 1.27, 12.33 ± 0.68, 21.2 ± 0.35 μM	[169]
3-hydroxyterphenyllin	*Galaxea fascicularis*	*Aspergillus tritici*	HeLa, A549, HepG2	IC_{50} = 23.37 ± 0.84, 36.07 ± 1.67, 32.10 ± 2.65 μM	[169]
3-hydroxy-4″-deoxyterphenyllin	*Galaxea fascicularis*	*Aspergillus tritici*	HepG2	IC_{50} = 45.20 ± 1.00 μM	[169]

3″-prenylterphenyllin	*Galaxea fascicularis*	*Aspergillus tritici*	HeLa, HepG2	IC_{50} = 38.30 ± 1.50, 40.10 ± 0.90 μM	[169]
Emodin	*Galaxea fascicularis*	*Aspergillus tritici*	HeLa, A549, HepG2	IC_{50} = 25.07 ± 0.81, 22.17 ± 1.45, 30.20 ± 0.87 μM	[169]
3-hydroxy-2-hydroxymethyl-1-methoxyanthracene-9,10-dione	*Galaxea fascicularis*	*Aspergillus tritici*	A549	IC_{50} = 45.63 ± 1.79 μM	[169]
1,2,3-trimethoxy-7-hydroxymethylanthracene-9,10-dione	*Galaxea fascicularis*	*Aspergillus tritici*	HepG2	IC_{50} = 42.07 ± 1.07 μM	[169]

Supplementary table S8. Antimicrobial compounds from coral-associated microorganisms

Compound	Coral species	Microorganisms	Reference microorganisms	Activity value	Ref.
Cottoquinazoline D	*Cladiella* sp.	*A. versicolor*	*C. albicans*	MIC = 22.6 μM	[170]
Watasemycin A	Unidentified Coral	*Streptomyces* sp.	*S. aureus, methicillin-resistant S. aureus (MRSA082, MRSA111, MRSA234)*	MIC = 1.95 μg/mL, 7.81, 7.81, 7.81 μg/mL	[168]
Aerugine	Unidentified Coral	*Streptomyces* sp.	*S. aureus, methicillin-resistant S. aureus (MRSA082, MRSA111, MRSA234)*	MIC = 1.95 μg/mL, 7.81, 7.81, 7.81 μg/mL	[168]
(±)-pestalachloride D	*Sarcophyton* sp.	*Pestalotiopsis* sp.	*E. coli, V. anguillarum, V. parahaemolyticus*	MIC = 5.0, 10.0, 20.0 μM	[171]
(±)-pestalachloride C	*Sarcophyton* sp.	*Pestalotiopsis* sp.	*E. coli, V. anguillarum, V. parahaemolyticus*	MIC = 5.0, 10.0, 20.0 μM	[171]
Secalonic acid D	*Dichotella gemmacea*	*Penicillium* sp.	*B. subtilis, E. coli, M. luteus, P. nigrifaciens*	MIC = 22.4, 22.4, 24.4, 97.5 μg/mL	[172]
Secalonic acid B	*Dichotella gemmacea*	*Penicillium* sp.	*B. subtilis, E. coli, M. luteus, P. nigrifaciens*	MIC = 97.5, 97.5, 97.5, 390.5 μg/mL	[172]
Penicillixanthone A	*Dichotella gemmacea*	*Penicillium* sp.	*B. subtilis, E. coli, M. luteus, P. nigrifaciens*	MIC = 24.4, 24.4, 24.4, 97.5 μg/mL	[172]
4′-OMe-asperphenamate	*Sarcophyton* sp.	*A. elegans*	*S. epidermidis*	MIC = 10 μM	[173]
Asperphenamate	*Sarcophyton* sp.	*A. elegans*	*S. epidermidis*	MIC = 10 μM	[173]
Cytochalasin I	*Sarcophyton* sp.	*A. elegans*	*S. epidermidis and S. aureus*	MIC = 20, 10 μM	[173]
Cytochalasin D	*Sarcophyton* sp.	*A. elegans*	*S. epidermidis, S. aureus, E. coli and B. cereus*	MIC = 10 μM	[173]
Aspergillin PZ	*Sarcophyton* sp.	*A. elegans*	*S. epidermidis*	MIC = 20 μM	[173]
6-deoxyaflaquinolone E	*Carijoa* sp.	*Scopulariopsis* sp.	*S. aureus, B. cereus, V. parahaemolyticus, N. brasiliensis, P. putida*	MIC = 0.78, 1.56, 6.25, 0.78, 1.56 μM	[174]
Tropodithietic acid	*Pocillopora damicornis*	*Pseudovibrio* sp.	*V. coralliilyticus and V. owensii.*	D = 2 ±0.09 and 5 ±0.07 mm	[175]
3, 9-deoxy-7-methoxybostrycin	*Galaxea fascicularis*	*A. tritici*	*S. aureus (MRSA) (ATCC 43300, CGMCC 1.12409)*	MIC = 7.53 ± 0.31, 7.63 ± 0.21 μg/mL	[169]
4-methyl-3″-prenylcandidusin A	*Galaxea fascicularis*	*A. tritici*	*MRSA ATCC 43300, MRSA CGMCC 1.12409, V. vulnificus, V. rotiferianus, V. campbellii*	MIC = 3.80 ± 0.13, 3.80 ± 0.22, 7.77 ± 0.10, 7.75 ± 0.18, 15.57 ± 0.30 μg/mL	[169]
4-methyl-candidusin A	*Galaxea fascicularis*	*A. tritici*	*MRSA ATCC 43300, MRSA CGMCC 1.12409, V. vulnificus, V. campbellii*	MIC = 31.33 ± 0.61, 30.97 ± 0.78, 31.47 ± 1.22, 15.10 ± 0.44 μg/mL	[169]
Aspetritone B	*Galaxea fascicularis*	*A. tritici*	*MRSA ATCC 43300, MRSA CGMCC 1.12409, V. vulnificus, V. rotiferianus, V. campbellii*	MIC = 15.27 ± 0.35, 15.63 ± 0.45, 15.47 ± 0.51, 31.33 ± 0.23, 15.77 ± 0.29 μg/mL	[169]
3,4-dimethyl-3″-prenylcandidusin A	*Galaxea fascicularis*	*A. tritici*	*MRSA ATCC 43300, MRSA CGMCC 1.12409, V. vulnificus, V. rotiferianus*	MIC = 15.67 ± 0.50, 7.57 ± 0.73, 15.58 ± 0.33, 15.57 ± 0.30 μg/mL	[169]

Candidusin A	*Galaxea fascicularis*	*A. tritici*	*MRSA ATCC 43300, MRSA CGMCC 1.12409, V. vulnificus, V. rotiferianus*	MIC = 31.47 ± 0.24, 31.23 ± 0.10, 31.42 ± 0.23, 31.33 ± 0.19 μg/mL	[169]
4″-deoxyterphenyllin	*Galaxea fascicularis*	*A. tritici*	*MRSA ATCC 43300, MRSA CGMCC 1.12409, V. vulnificus, V. rotiferianus, V. campbellii*	MIC = 31.30 ± 0.26, 31.45 ± 0.22, 31.37 ± 0.14, 31.53 ± 0.31, 31.47 ± 0.25 μg/mL	[169]
3-prenylterphenyllin	*Galaxea fascicularis*	*A. tritici*	*MRSA ATCC 43300, MRSA CGMCC 1.12409, V. vulnificus, V. rotiferianus*	MIC = 15.53 ± 0.31, 15.47 ± 0.23, 31.43 ± 0.32, 31.37 ± 0.21 μg/mL	[169]
Terphenyllin	*Galaxea fascicularis*	*A. tritici*	*MRSA ATCC 43300, MRSA CGMCC 1.12409, V. vulnificus*	MIC = 31.47 ± 0.24, 31.27 ± 0.16, 31.27 ± 0.25 μg/mL	[169]
3-hydroxyterphenyllin	*Galaxea fascicularis*	*A. tritici*	*MRSA ATCC 43300, MRSA CGMCC 1.12409, V. vulnificus, V. rotiferianus, V. campbellii*	MIC = 31.30 ± 0.17, 31.33 ± 0.12, 31.43 ± 0.21 μg/mL	[169]
3″-prenylterphenyllin	*Galaxea fascicularis*	*A. tritici*	*MRSA ATCC 43300, MRSA CGMCC 1.12409, V. vulnificus, V. campbellii*	MIC = 31.33 ± 0.23, 31.28 ± 0.10, 31.25 ± 0.13, 31.43 ± 0.20 μg/mL	[169]
Emodin	*Galaxea fascicularis*	*A. tritici*	*MRSA ATCC 43300, MRSA CGMCC 1.12409, V. vulnificus, V. rotiferianus, V. campbellii*	MIC = 15.65 ± 0.18, 15.53 ± 0.12, 15.73 ± 0.12, 62.67 ± 0.15, 31.35 ± 0.22 μg/mL	[169]
3-hydroxy- 1,2,5,6-tetramethoxyanthracene-9,10-dione	*Galaxea fascicularis*	*A. tritici*	*MRSA ATCC 43300, MRSA CGMCC 1.12409*	MIC = 31.32 ± 0.25, 31.33 ± 0.23 μg/mL	[169]
3-hydroxy-2-hydroxymethyl-1-methoxyanthracene-9,10-dione	*Galaxea fascicularis*	*A. tritici*	*V. rotiferianus*	MIC = 31.28 ± 0.14 μg/mL	[169]
Lobophorin K	*Lophelia pertusa*	*Streptomyces* sp.	*S. aureus*	MIC_{90} = 40 - 80 μg/mL	[176]
Cell-free supernatant	*Platygyra* sp.	*Pseudoalteromonas* sp.	*B. cereus, S. aureus*	Survival rate: 15 - 46 % and 34 - 69 %	[177]
Agar containing bacteria	*Antipathes dichotoma*	*B. aquimaris*	*M. luteus, P. piscida*	D = 8.06 ± 0.71, 11.28 ± 0.72 mm	[178]
Agar containing bacteria	*Antipathes dichotoma*	*B. flexus*	*M. luteus, P. piscida*	D = 7.07 ± 0.14, 13.09 ± 0.46 mm	[178]
Agar containing bacteria	*Antipathes dichotoma*	*B. altitudinis*	*M. luteus*	D = 10.28 ± 0.42 mm	[178]
Agar containing bacteria	*Antipathes dichotoma*	*S. albus*	*M. luteus*	D = 11.29 ± 0.35 mm	[178]
Agar containing bacteria	*Antipathes dichotoma*	*S. labedae*	*M. luteus*	D = 12.69 ± 0.44 mm	[178]
Agar containing bacteria	*Antipathes dichotoma*	*P. oxalicum*	*M. luteus*	D = 8.23 ± 0.21 mm	[178]
Agar containing bacteria	*Antipathes dichotoma*	*B. amyloliquefaciens*	*M. luteus, P. piscida*	D = 8.17 ± 0.32 , 11.23 ± 0.56 mm	[178]
Agar containing bacteria	*Antipathes dichotoma*	*P. chrysogenum*	*M. luteus, P. piscida*	D = 9.28 ± 0.28, 11.41 ± 0.77 mm	[178]

Agar containing bacteria	*Antipathes dichotoma*	*P. citrinum*	*M. luteus, P. piscida*	D = 8.18 ± 0.61, 14.00 ± 0.70 mm	[178]
Agar containing bacteria	*Antipathes dichotoma*	*S. equorum*	*P. piscida*	D = 11.41 ± 0.73 mm	[178]
Agar containing bacteria	*Antipathes dichotoma*	*F. proliferatum*	*A. versicolor*	D = 15.45 ± 0.49 mm	[178]
Agar containing bacteria	*Antipathes dichotoma*	*B. subtilis*	*A. versicolor, A. sydowii*	D = 16.95 ± 0.63, 15.23 ± 0.59 mm	[178]
Agar containing bacteria	*Antipathes dichotoma*	*A. ochraceopetaliformis*	*A. versicolor, A. sydowii*	D = 17.15 ± 0.69, 11.62 ± 0.38 mm	[178]
Agar containing bacteria	*Antipathes dichotoma*	*M. coxensis*	*M. luteus, A. versicolor, A. sydowii*	D = 8.03 ± 0.82 , 11.22 ± 0.84, 8.12 ± 0.14 mm	[178]
Agar containing bacteria	*Antipathes dichotoma*	*S. albogriseolus*	*M. luteus, P. piscida, A.versicolor, A. sydowii*	D = 15.29 ± 0.42, 13.08 ± 0.65, 7.56 ± 0.39, 11.87 ± 0.72 mm	[178]
Agar containing bacteria	*Antipathes dichotoma*	*S. xiamenensis*	*M. luteus, P. piscida, A.versicolor, A. sydowii*	D = 16.25 ± 0.38, 16.94 ± 0.56, 14.33 ± 0.62, 9.13 ± 0.30 mm	[178]

Supplementary table S9. Antiviral compounds from coral-associated microorganisms

Compound	Coral species	Microorganisms	Target virus	Activity value	Ref.
Tetrahydroaltersolanol C	Sarcophyton sp.	Alternaria sp.	PRRSV	IC_{50} = 65 μM	[166]
Alterporriol Q	Sarcophyton sp.	Alternaria sp.	PRRSV	IC_{50} = 22 μM	[166]
Alterporriol C	Sarcophyton sp.	Alternaria sp.	PRRSV	IC_{50} = 39 μM	[166]
Pestalotiolide A	Sarcophyton sp.	Pestalotiopsis sp.	EV71, RSV	IC_{50} = 27.7, 80.9 μM	[179]
7-Hydroxy-5-Methoxy-4,6-Dimethyl-7-O-Β-D-Glucopyranosyl-Phthalide	Sarcophyton sp.	Pestalotiopsis sp.	EV71, RSV, HSV-1	IC_{50} = 51.6, 25.6, 63.9 μM	[179]
7-hydroxy-5-methoxy-4,6-dimethyl-7-O-Α-L-rhamnosyl-phthalide	Sarcophyton sp.	Pestalotiopsis sp.	EV71	IC_{50} = 111 μM	[179]
7-hydroxy-5-methoxy-4,6-dimethylphthalidc	Sarcophyton sp.	Pestalotiopsis sp.	RSV	IC_{50} = 21 μM	[179]
5'-*O*-Acetyl uridine	Sarcophyton sp.	Pestalotiopsis sp.	EV71	IC_{50} = 110μM	[179]

Supplementary table S10. Antifouling compounds from coral-associated microorganisms

Compound	Coral species	Microorganisms	Fouling organisms	Activity value	Ref.
Cytochalasin I	*Sarcophyton* sp.	*Aspergillus elegans*	The larval of the barnacle *Balanus amphitrite*	EC_{50} = 34 μM	[173]
Cytochalasin J	*Sarcophyton* sp.	*Aspergillus elegans*	The larval of the barnacle *Balanus amphitrite*	EC_{50} = 14 μM	[173]
Cytochalasin D	*Sarcophyton* sp.	*Aspergillus elegans*	The larval of the barnacle *Balanus amphitrite*	EC_{50} = 6.2 μM	[173]
Cytochalasin H	*Sarcophyton* sp.	*Aspergillus elegans*	The larval of the barnacle *Balanus amphitrite*	EC_{50} = 37 μM	[173]
6,8,5'6'-tetrahydroxy-3'-methylflavone	*Dichotella gemmacea*	*Penicillium* sp.	The larval of the barnacle *Balanus amphitrite*	EC_{50} = 6.7 μg/mL	[172]
Emodin	*Dichotella gemmacea*	*Penicillium* sp.	The larval of the barnacle *Balanus amphitrite*	EC_{50} = 6.1 μg/mL	[172]
Citreorosein	*Dichotella gemmacea*	*Penicillium* sp.	The larval of the barnacle *Balanus amphitrite*	EC_{50} = 17.9 μg/mL	[172]
Isorhodoptilometrin	*Dichotella gemmacea*	*Penicillium* sp.	The larval of the barnacle *Balanus amphitrite*	EC_{50} = 13.7 μg/mL	[172]
Aniduquinolone A	*Carijoa* sp.	*Scopulariopsis* sp.	The larval of the barnacle *Balanus amphitrite*	EC_{50} = 17.5 pM	[174]
Aflaquinolone A	*Carijoa* sp.	*Scopulariopsis* sp.	The larval of the barnacle *Balanus amphitrite*	EC_{50} = 28 nM	[174]
Aflaquinolone D	*Carijoa* sp.	*Scopulariopsis* sp.	The larval of the barnacle *Balanus amphitrite*	EC_{50} = 2.8 nM	[174]
6-deoxyaflaquinolone E	*Carijoa* sp.	*Scopulariopsis* sp.	The larval of the barnacle *Balanus amphitrite*	EC_{50} = 1.04 μM	[174]
Aflaquinolone F	*Carijoa* sp.	*Scopulariopsis* sp.	The larval of the barnacle *Balanus amphitrite*	EC_{50} = 0.86 μM	[174]

Supplementary table S11. Other bioactive compounds from coral-associated microorganisms

Compound	Coral species	Microorganisms	Activity type	Activity value	Ref.
Alternariol-9-methyl ether-3-O-sulphate	*Litophyton arboreum*	*Alternaria alternata*	Protease inhibitory activity (HCV NS3-NS4A)	IC_{50} = 52.0 μg/mL	[180]
Alternar-iol-9-methyl ether	*Litophyton arboreum*	*Alternaria alternata*	Protease inhibitory activity (HCV NS3-NS4A)	IC_{50} = 32.2 μg/mL	[180]
Alternariol	*Litophyton arboreum*	*Alternaria alternata*	Protease inhibitory activity (HCV NS3-NS4A)	IC_{50} = 12.0 μg/mL	[180]
Ethyl acetate extract	*Litophyton arboreum*	*Alternaria alternata*	Protease inhibitory activity (HCV NS3-NS4A)	IC_{50} = 14.0 μg/mL	[180]
Cladospolide E	Unidentified soft coral	*Cladosporium* sp.	Lipid-lowering activity	IC_{50} = 12.1 μM	[181]
Secopatulolide A	Unidentified soft coral	*Cladosporium* sp.	Lipid-lowering activity	IC_{50} = 8.4 μM	[181]
Secopatulolide B	Unidentified soft coral	*Cladosporium* sp.	Lipid-lowering activity	IC_{50} = 13.1 μM	[181]
11-hydroxy-ɣ-dodecalactone	Unidentified soft coral	*Cladosporium* sp.	Lipid-lowering activity	IC_{50} = 7.1 μM	[181]
Eurothiocin A	*Sarcophyton* sp.	*Eurotium rubrum*	α-glucosidase inhibitory activity	IC_{50} = 17.1 μM	[182]
Eurothiocin B	*Sarcophyton* sp.	*Eurotium rubrum*	α-glucosidase inhibitory activity	IC_{50} = 42.6 μM	[182]
Butyrolactone I	*Sarcophyton* sp.	*Eurotium rubrum*	α-glucosidase inhibitory activity	IC_{50} = 98.5 μM	[182]
Aspernolide D	*Sarcophyton* sp.	*Eurotium rubrum*	α-glucosidase inhibitory activity	IC_{50} = 110.8 μM	[182]
Vermistatin	*Sarcophyton* sp.	*Eurotium rubrum*	α-glucosidase inhibitory activity	IC_{50} = 107.1 μM	[182]
Methoxyvermistatin	*Sarcophyton* sp.	*Eurotium rubrum*	α-glucosidase inhibitory activity	IC_{50} = 236 μM	[182]
(±)-asperteretone A	*Sarcophyton subviride*	*Aspergillus terreus*	α-glucosidase inhibitory activity	IC_{50} = 45.4 ± 3.8 to 53.1 ± 1.4 μM	[165]
(±)-asperteretone B	*Sarcophyton subviride*	*Aspergillus terreus*	α-glucosidase inhibitory activity	IC_{50} = 17.3 ± 2.4 to 19.2 ± 1.9 μM	[165]
(±)-asperteretone C	*Sarcophyton subviride*	*Aspergillus terreus*	α-glucosidase inhibitory activity	IC_{50} = 49.8 ± 5.7 to 52.2 ± 4.64 μM	[165]
(±)-asperteretone D	*Sarcophyton subviride*	*Aspergillus terreus*	α-glucosidase inhibitory activity	IC_{50} = 15.7 ± 1.1 to 18.9 ± 2.3 μM	[165]
Asperteretone E	*Sarcophyton subviride*	*Aspergillus terreus*	α-glucosidase inhibitory activity	IC_{50} = 48.9 ± 7.3 μM	[165]
Tetraorcinol A	*Cladiella* sp.	*Aspergillus versicolor*	DPPH radical scavenging activity	IC_{50} = 67 μM	[170]

References

1. Yan, P.; Lv, Y.; van Ofwegen, L.; Proksch, P.; Lin, W. Lobophytones A−G, new isobiscembranoids from the soft coral *Lobophytum pauciflorum*. *Org. Lett.* **2010**, *12*, 2484-2487, doi:10.1021/ol100567d.
2. Lu, Y.; Su, J.-H.; Huang, C.-Y.; Liu, Y.-C.; Kuo, Y.-H.; Wen, Z.-H.; Hsu, C.-H.; Sheu, J.-H. Cembranoids from the soft corals *Sinularia granosa* and *Sinularia* querciformis. *Chem. Pharm. Bull.* **2010**, *58*, 464-466.
3. Cheng, S.-Y.; Chuang, C.-T.; Wang, S.-K.; Wen, Z.-H.; Chiou, S.-F.; Hsu, C.-H.; Dai, C.-F.; Duh, C.-Y. Antiviral and anti-inflammatory diterpenoids from the soft coral *Sinularia gyrosa*. *J. Nat. Prod.* **2010**, *73*, 1184-1187.
4. Chen, B.W.; Chang, S.M.; Huang, C.Y.; Chao, C.H.; Su, J.H.; Wen, Z.H.; Hsu, C.H.; Dai, C.F.; Wu, Y.C.; Sheu, J.H. Hirsutalins A-H, eunicellin-based diterpenoids from the soft coral *Cladiella hirsuta*. *J. Nat. Prod.* **2010**, *73*, 1785–1791.
5. Cheng, S.Y.; Lin, E.H.; Wen, Z.H.; Chiang, M.Y.; Duh, C.Y. Two new verticillane-type diterpenoids from the Formosan soft coral *Cespitularia hypotentaculata*. *Chem. Pharm. Bull.* **2010**, *58*, 848—851.
6. Chen, B.W.; Chao, C.H.; Su, J.H.; Tsai, C.W.; Wang, W.H.; Wen, Z.H.; Huang, C.Y.; Sung, P.J.; Wu, Y.C.; Sheu, J.H. Klysimplexins I-T, eunicellin-based diterpenoids from the cultured soft coral *Klyxum simplex*. *Org. Biomol. Chem.* **2011**, *9*, 834-844, doi:10.1039/c0ob00351d.
7. Quang, T.H.; Ha, T.T.; Minh, C.V.; Kiem, P.V.; Huong, H.T.; Ngan, N.T.; Nhiem, N.X.; Tung, N.H.; Tai, B.H.; Thuy, D.T., et al. Cytotoxic and anti-inflammatory cembranoids from the Vietnamese soft coral *Lobophytum laevigatum*. *Bioorg. Med. Chem.* **2011**, *19*, 2625-2632, doi:10.1016/j.bmc.2011.03.009.
8. Su, J.H.; Wen, Z.H. Bioactive cembrane-based diterpenoids from the soft coral *Sinularia triangular*. *Mar. Drugs* **2011**, *9*, 944-951, doi:10.3390/md9060944.
9. Zhao, C.; Xu, J.-J.; Wang, J.; Li, S.-Y.; Qiao, W.; Tang, S.-A. Five new cembrane diterpenoids from the South China Sea soft coral *Sinularia flexibilis*. *Phytochem. Lett.* **2018**, *25*, 180-183, doi:10.1016/j.phytol.2018.04.012.
10. Kao, C.Y.; Su, J.H.; Lu, M.C.; Hwang, T.L.; Wang, W.H.; Chen, J.J.; Sheu, J.H.; Kuo, Y.H.; Weng, C.F.; Fang, L.S., et al. Lobocrassins A-E: new cembrane-type diterpenoids from the soft coral *Lobophytum crassum*. *Mar. Drugs* **2011**, *9*, 1319-1331, doi:10.3390/md9081319.
11. Chao, C.H.; Chou, K.J.; Huang, C.Y.; Wen, Z.H.; Hsu, C.H.; Wu, Y.C.; Dai, C.F.; Sheu, J.H. Bioactive cembranoids from the soft coral *Sinularia crassa*. *Mar. Drugs* **2011**, *9*, 1955-1968, doi:10.3390/md9101955.
12. Tai, C.J.; Su, J.H.; Huang, M.S.; Wen, Z.H.; Dai, C.F.; Sheu, J.H. Bioactive eunicellin-based diterpenoids from the soft coral *Cladiella krempfi*. *Mar. Drugs* **2011**, *9*, 2036-2045, doi:10.3390/md9102036.
13. Chao, C.H.; Chou, K.J.; Wen, Z.H.; Wang, G.H.; Wu, Y.C.; Dai, C.F.; Sheu, J.H. Paraminabeolides A-F, cytotoxic and anti-inflammatory marine withanolides from the soft coral *Paraminabea acronocephala*. *J. Nat. Prod.* **2011**, *74*, 1132-1141, doi:10.1021/np2000705.
14. Hsu, F.-J.; Chen, B.-W.; Wen, Z.-H.; Huang, C.-Y.; Dai, C.-F.; Su, J.-H.; Wu, Y.-C.; Sheu, J.-H. Klymollins A–H, bioactive eunicellin-based diterpenoids from the Formosan soft coral *Klyxum molle*. *J. Nat. Prod.* **2011**, *74*, 2467-2471.
15. Lin, W.Y.; Lu, Y.; Su, J.H.; Wen, Z.H.; Dai, C.F.; Kuo, Y.H.; Sheu, J.H. Bioactive cembranoids from the dongsha atoll soft coral *Sarcophyton crassocaule*. *Mar. Drugs* **2011**, *9*, 994-1006, doi:10.3390/md9060994.
16. Putra, M.Y.; Ianaro, A.; Panza, E.; Bavestrello, G.; Cerrano, C.; Fattorusso, E.; Taglialatela-Scafati, O. Sinularioside, a triacetylated glycolipid from the Indonesian soft coral *Sinularia* sp., is an inhibitor of NO release. *Bioorg. Med. Chem. Lett.* **2012**, *22*, 2723-2725, doi:10.1016/j.bmcl.2012.02.102.
17. Su, J.H.; Huang, C.Y.; Li, P.J.; Lu, Y.; Wen, Z.H.; Kao, Y.H.; Sheu, J.H. Bioactive cadinane-type compounds from the soft coral *Sinularia scabra*. *Arch. Pharm. Res.* **2012**, *35*, 779-784, doi:10.1007/s12272-012-0503-2.
18. Chung, H.M.; Hong, P.H.; Su, J.H.; Hwang, T.L.; Lu, M.C.; Fang, L.S.; Wu, Y.C.; Li, J.J.; Chen, J.J.; Wang, W.H., et al. Bioactive compounds from a gorgonian coral *Echinomuricea* sp. (Plexauridae). *Mar. Drugs* **2012**, *10*, 1169-1179, doi:10.3390/md10051169.

19. Tseng, Y.J.; Shen, K.P.; Lin, H.L.; Huang, C.Y.; Dai, C.F.; Sheu, J.H. Lochmolins A-G, new sesquiterpenoids from the soft coral *Sinularia lochmodes*. *Mar. Drugs* **2012**, *10*, 1572-1581, doi:10.3390/md10071572.
20. Chao, C.H.; Chou, K.J.; Huang, C.Y.; Wen, Z.H.; Hsu, C.H.; Wu, Y.C.; Dai, C.F.; Sheu, J.H. Steroids from the soft coral *Sinularia crassa*. *Mar. Drugs* **2012**, *10*, 439-450, doi:10.3390/md10020439.
21. Lin, W.Y.; Lu, Y.; Chen, B.W.; Huang, C.Y.; Su, J.H.; Wen, Z.H.; Dai, C.F.; Kuo, Y.H.; Sheu, J.H. Sarcocrassocolides M-O, bioactive cembranoids from the Dongsha Atoll soft coral *Sarcophyton crassocaule*. *Mar. Drugs* **2012**, *10*, 617-626, doi:10.3390/md10030617.
22. Fang, H.-Y.; Liaw, C.-C.; Chao, C.-H.; Wen, Z.-H.; Wu, Y.-C.; Hsu, C.-H.; Dai, C.-F.; Sheu, J.-H. Bioactive pregnane-type steroids from the soft coral *Scleronephthya gracillimum*. *Tetrahedron* **2012**, *68*, 9694-9700, doi:10.1016/j.tet.2012.09.060.
23. Huang, C.Y.; Su, J.H.; Duh, C.Y.; Chen, B.W.; Wen, Z.H.; Kuo, Y.H.; Sheu, J.H. A new 9,11-secosterol from the soft coral *Sinularia granosa*. *Bioorg. Med. Chem. Lett.* **2012**, *22*, 4373-4376, doi:10.1016/j.bmcl.2012.05.002.
24. Cheng, C.H.; Chung, H.M.; Hwang, T.L.; Lu, M.C.; Wen, Z.H.; Kuo, Y.H.; Wang, W.H.; Sung, P.J. Echinoclerodane A: a new bioactive clerodane-type diterpenoid from a gorgonian coral *Echinomuricea* sp. *Molecules* **2012**, *17*, 9443-9450, doi:10.3390/molecules17089443.
25. Chen, Y.-H.; Hwang, T.-L.; Su, Y.-D.; Chang, Y.-C.; Chen, Y.-H.; Hong, P.-H.; Hu, L.-C.; Yen, W.-H.; Hsu, H.-Y.; Huang, S.-J. New 6-hydroxyeunicellins from a soft coral *Cladiella* sp. *Chem. Pharm. Bull.* **2012**, *60*, 160-163.
26. Yang, B.; Zhou, X.; Huang, H.; Yang, X.-W.; Liu, J.; Lin, X.; Li, X.; Peng, Y.; Liu, Y. New cembrane diterpenoids from a Hainan soft coral *Sinularia* sp. *Mar. Drugs* **2012**, *10*, doi:10.3390/md10092023.
27. Chen, D.; Chen, W.; Liu, D.; van Ofwegen, L.; Proksch, P.; Lin, W. Asteriscane-type sesquiterpenoids from the soft coral *Sinularia capillosa*. *J. Nat. Prod.* **2013**, *76*, 1753-1763, doi:10.1021/np400480p.
28. Tai, C.J.; Su, J.H.; Huang, C.Y.; Huang, M.S.; Wen, Z.H.; Dai, C.F.; Sheu, J.H. Cytotoxic and anti-inflammatory eunicellin-based diterpenoids from the soft coral *Cladiella krempfi*. *Mar. Drugs* **2013**, *11*, 788-799, doi:10.3390/md11030788.
29. Thao, N.P.; Nam, N.H.; Cuong, N.X.; Quang, T.H.; Tung, P.T.; Dat le, D.; Chae, D.; Kim, S.; Koh, Y.S.; Kiem, P.V., et al. Anti-inflammatory norditerpenoids from the soft coral *Sinularia maxima*. *Bioorg. Med. Chem. Lett.* **2013**, *23*, 228-231, doi:10.1016/j.bmcl.2012.10.129.
30. Nguyen, P.T.; Nguyen, H.N.; Nguyen, X.C.; Nguyen, X.N.; Pham, T.T.; Tran, H.Q.; Nguyen, T.T.N.; Phan, V.K.; Chau, V.M.; Kim, Y.H. A new sterol from the soft coral *Lobophytum crassum*. *Bull. Korean Chem. Soc.* **2013**, *34*, 249-251, doi:10.5012/bkcs.2013.34.1.249.
31. Fang, H.Y.; Hsu, C.H.; Chao, C.H.; Wen, Z.H.; Wu, Y.C.; Dai, C.F.; Sheu, J.H. Cytotoxic and anti-inflammatory metabolites from the soft coral Scleronephthya gracillimum. *Mar. Drugs* **2013**, *11*, 1853-1865, doi:10.3390/md11061853.
32. Yin, J.; Zhao, M.; Ma, M.; Xu, Y.; Xiang, Z.; Cai, Y.; Dong, J.; Lei, X.; Huang, K.; Yan, P. New casbane diterpenoids from a South China Sea soft coral, *Sinularia* sp. *Mar. Drugs* **2013**, *11*, 455-465, doi:10.3390/md11020455.
33. Cheng, Y.B.; Fazary, A.E.; Lin, Y.C.; Lo, I.W.; Ong, S.C.; Chen, S.Y.; Chien, C.T.; Lin, Y.J.; Lin, W.W.; Shen, Y.C. Hyperinakin, a new anti-inflammatory phloroglucinol derivative from *Hypericum nakamurai*. *Nat. Prod. Res.* **2013**, *27*, 727-734, doi:10.1080/14786419.2012.695365.
34. Chao, C.H.; Wu, Y.C.; Wen, Z.H.; Sheu, J.H. Steroidal carboxylic acids from soft coral *Paraminabea acronocephala*. *Mar. Drugs* **2013**, *11*, 136-145, doi:10.3390/md11010136.
35. Lin, Y.F.; Kuo, C.Y.; Wen, Z.H.; Lin, Y.Y.; Wang, W.H.; Su, J.H.; Sheu, J.H.; Sung, P.J. Flexibilisquinone, a new anti-inflammatory quinone from the cultured soft coral *Sinularia flexibilis*. *Molecules* **2013**, *18*, 8160-8167, doi:10.3390/molecules18078160.
36. Lee, Y.N.; Tai, C.J.; Hwang, T.L.; Sheu, J.H. Krempfielins J-M, new eunicellin-based diterpenoids from the soft coral *Cladiella krempfi*. *Mar. Drugs* **2013**, *11*, 2741-2750, doi:10.3390/md11082741.
37. Lin, M.C.; Chen, B.W.; Huang, C.Y.; Dai, C.F.; Hwang, T.L.; Sheu, J.H. Eunicellin-based diterpenoids from the Formosan soft coral *Klyxum molle* with inhibitory activity on superoxide generation and elastase release by neutrophils. *J. Nat. Prod.* **2013**, *76*, 1661-1667, doi:10.1021/np400372v.

38. Chen, B.-W.; Wang, S.-Y.; Huang, C.-Y.; Chen, S.-L.; Wu, Y.-C.; Sheu, J.-H. Hirsutalins I–M, eunicellin-based diterpenoids from the soft coral *Cladiella hirsuta*. *Tetrahedron* **2013**, *69*, 2296-2301, doi:10.1016/j.tet.2013.01.015.
39. Yang, B.; Liao, S.; Lin, X.; Wang, J.; Liu, J.; Zhou, X.; Yang, X.; Liu, Y. New sinularianin sesquiterpenes from soft coral *Sinularia* sp. *Mar. Drugs* **2013**, *11*, 4741-4750, doi:10.3390/md11124741.
40. Thao, N.P.; Luyen, B.T.; Ngan, N.T.; Song, S.B.; Cuong, N.X.; Nam, N.H.; Kiem, P.V.; Kim, Y.H.; Minh, C.V. New anti-inflammatory cembranoid diterpenoids from the Vietnamese soft coral *Lobophytum crassum*. *Bioorg. Med. Chem. Lett.* **2014**, *24*, 228-232, doi:10.1016/j.bmcl.2013.11.033.
41. Cuong, N.X.; Thao, N.P.; Luyen, B.T.T.; Ngan, N.T.T.; Thuy, D.T.T.; Song, S.B.; Nam, N.H.; Van Kiem, P.; Kim, Y.H.; Van Minh, C. Cembranoid diterpenes from the soft coral *Lobophytum crassum* and their anti-inflammatory activities. *Chem. Pharm. Bull.* **2014**, *62*, 203-208.
42. Thao, N.P.; Nam, N.H.; Cuong, N.X.; Luyen, B.T.; Tai, B.H.; Kim, J.E.; Song, S.B.; Kiem, P.V.; Minh, C.V.; Kim, Y.H. Inhibition of NF-kappaB transcriptional activation in HepG2 cells by diterpenoids from the soft coral *Sinularia maxima*. *Arch. Pharm. Res.* **2014**, *37*, 706-712, doi:10.1007/s12272-013-0230-3.
43. Chung, H.M.; Wang, W.H.; Hwang, T.L.; Chen, J.J.; Fang, L.S.; Wen, Z.H.; Wang, Y.B.; Wu, Y.C.; Sung, P.J. Rumphellols A and B, new caryophyllene sesquiterpenoids from a Formosan gorgonian coral, *Rumphella antipathies*. *Int. J. Mol. Sci.* **2014**, *15*, 15679-15688, doi:10.3390/ijms150915679.
44. Liaw, C.C.; Cheng, Y.B.; Lin, Y.S.; Kuo, Y.H.; Hwang, T.L.; Shen, Y.C. New briarane diterpenoids from Taiwanese soft coral *Briareum violacea*. *Mar. Drugs* **2014**, *12*, 4677-4692, doi:10.3390/md12084677.
45. Chung, H.M.; Wang, W.H.; Hwang, T.L.; Fang, L.S.; Wen, Z.H.; Chen, J.J.; Wu, Y.C.; Sung, P.J. Rumphellaoic acid A, a novel sesquiterpenoid from the formosan gorgonian coral *Rumphella antipathies*. *Mar. Drugs* **2014**, *12*, 5856-5863, doi:10.3390/md12125856.
46. Thao, N.P.; Luyen, B.T.; Sun, Y.N.; Song, S.B.; Thanh, N.V.; Cuong, N.X.; Nam, N.H.; Kiem, P.V.; Kim, Y.H.; Minh, C.V. NF-kappaB inhibitory activity of polyoxygenated steroids from the Vietnamese soft coral *Sarcophyton pauciplicatum*. *Bioorg. Med. Chem. Lett.* **2014**, *24*, 2834-2838, doi:10.1016/j.bmcl.2014.04.103.
47. Lee, Y.N.; Tai, C.J.; Hwang, T.L.; Sheu, J.H. Krempfielins N-P, new anti-inflammatory eunicellins from a Taiwanese soft coral *Cladiella krempfi*. *Mar. Drugs* **2014**, *12*, 1148-1156, doi:10.3390/md12021148.
48. Lin, K.H.; Tseng, Y.J.; Chen, B.W.; Hwang, T.L.; Chen, H.Y.; Dai, C.F.; Sheu, J.H. Tortuosenes A and B, new diterpenoid metabolites from the Formosan soft coral *Sarcophyton tortuosum*. *Org. Lett.* **2014**, *16*, 1314-1317, doi:10.1021/ol403723b.
49. Lin, W.Y.; Chen, B.W.; Huang, C.Y.; Wen, Z.H.; Sung, P.J.; Su, J.H.; Dai, C.F.; Sheu, J.H. Bioactive cembranoids, sarcocrassocolides P-R, from the Dongsha Atoll soft coral *Sarcophyton crassocaule*. *Mar. Drugs* **2014**, *12*, 840-850, doi:10.3390/md12020840.
50. Huang, T.Z.; Chen, B.W.; Huang, C.Y.; Hwang, T.L.; Dai, C.F.; Sheu, J.H. Eunicellin-based diterpenoids, hirsutalins N-R, from the formosan soft coral *Cladiella hirsuta*. *Mar. Drugs* **2014**, *12*, 2446-2457, doi:10.3390/md12052446.
51. Chang, F.Y.; Hsu, F.J.; Tai, C.J.; Wei, W.C.; Yang, N.S.; Sheu, J.H. Klymollins T-X, bioactive eunicellin-based diterpenoids from the soft coral *Klyxum molle*. *Mar. Drugs* **2014**, *12*, 3060-3071, doi:10.3390/md12053060.
52. Tai, C.J.; Chokkalingam, U.; Cheng, Y.; Shih, S.P.; Lu, M.C.; Su, J.H.; Hwang, T.L.; Sheu, J.H. Krempfielins Q and R, two new eunicellin-based diterpenoids from the soft coral *Cladiella krempfi*. *Int. J. Mol. Sci.* **2014**, *15*, 21865-21874, doi:10.3390/ijms151221865.
53. Phan, C.S.; Ng, S.Y.; Kim, E.A.; Jeon, Y.J.; Palaniveloo, K.; Vairappan, C.S. Capgermacrenes A and B, bioactive secondary metabolites from a Bornean soft coral, *Capnella* sp. *Mar. Drugs* **2015**, *13*, 3103-3115, doi:10.3390/md13053103.
54. Huang, T.Z.; Chen, B.W.; Huang, C.Y.; Hwang, T.L.; Uvarani, C.; Dai, C.F.; Sung, P.J.; Su, J.H.; Sheu, J.H. Eunicellin-based diterpenoids, Hirsutalins S-V, from the Formosan soft coral *Cladiella hirsuta*. *Mar. Drugs* **2015**, *13*, 2757-2769, doi:10.3390/md13052757.
55. Chao, C.-H.; Huang, T.-Z.; Wu, C.-Y.; Chen, B.-W.; Huang, C.-Y.; Hwang, T.-L.; Dai, C.-F.; Sheu, J.-H. Steroidal and α-tocopherylhydroquinone glycosides from two soft corals *Cladiella hirsuta* and *Sinularia nanolobata*. *RSC Adv.* **2015**, *5*, 74256-74262, doi:10.1039/c5ra13436f.

56. Huang, C.Y.; Tseng, Y.J.; Chokkalingam, U.; Hwang, T.L.; Hsu, C.H.; Dai, C.F.; Sung, P.J.; Sheu, J.H. Bioactive isoprenoid-derived natural products from a Dongsha Atoll soft coral *Sinularia erecta*. *J. Nat. Prod.* **2016**, *79*, 1339-1346, doi:10.1021/acs.jnatprod.5b01142.
57. Tsai, C.-R.; Huang, C.-Y.; Chen, B.-W.; Tsai, Y.-Y.; Shih, S.-P.; Hwang, T.-L.; Dai, C.-F.; Wang, S.-Y.; Sheu, J.-H. New bioactive steroids from the soft coral *Klyxum flaccidum*. *RSC Adv.* **2015**, *5*, 12546-12554, doi:10.1039/c4ra13977a.
58. Huang, C.Y.; Sung, P.J.; Uvarani, C.; Su, J.H.; Lu, M.C.; Hwang, T.L.; Dai, C.F.; Wu, S.L.; Sheu, J.H. Glaucumolides A and B, biscembranoids with new structural type from a cultured soft coral *Sarcophyton glaucum*. *Sci. Rep.* **2015**, *5*, 15624, doi:10.1038/srep15624.
59. Hsiao, T.H.; Sung, C.S.; Lan, Y.H.; Wang, Y.C.; Lu, M.C.; Wen, Z.H.; Wu, Y.C.; Sung, P.J. New anti-inflammatory cembranes from the cultured soft coral *Nephthea columnaris*. *Mar. Drugs* **2015**, *13*, 3443-3453, doi:10.3390/md13063443.
60. Yin, C.-T.; Wen, Z.-H.; Lan, Y.-H.; Chang, Y.-C.; Wu, Y.-C.; Sung, P.-J. New anti-inflammatory norcembranoids from the soft coral *Sinularia numerosa*. *Chem. Pharm. Bull.* **2015**, *63*, 752-756.
61. Roy, P.K.; Ashimine, R.; Miyazato, H.; Taira, J.; Ueda, K. New casbane and cembrane diterpenoids from an Okinawan soft coral, *Lobophytum* sp. *Molecules* **2016**, *21*, doi:10.3390/molecules21050679.
62. Tsai, Y.Y.; Huang, C.Y.; Tseng, W.R.; Chiang, P.L.; Hwang, T.L.; Su, J.H.; Sung, P.J.; Dai, C.F.; Sheu, J.H. Klyflaccisteroids K-M, bioactive steroidal derivatives from a soft coral *Klyxum flaccidum*. *Bioorg. Med. Chem. Lett.* **2017**, *27*, 1220-1224, doi:10.1016/j.bmcl.2017.01.060.
63. Chang, Y.C.; Kuo, L.M.; Hwang, T.L.; Yeh, J.; Wen, J.H.; Fang, L.S.; Wu, Y.C.; Lin, C.S.; Sheu, J.H.; Sung, P.J. Pinnisterols A–C, new 9,11-secosterols from a Gorgonian *Pinnigorgia* sp. *Mar. Drugs* **2016**, *14*, doi:10.3390/md14010012.
64. Huang, C.Y.; Chang, C.W.; Tseng, Y.J.; Lee, J.; Sung, P.J.; Su, J.H.; Hwang, T.L.; Dai, C.F.; Wang, H.C.; Sheu, J.H. Bioactive steroids from the Formosan soft coral *Umbellulifera petasites*. *Mar. Drugs* **2016**, *14*, doi:10.3390/md14100180.
65. Hassan, M.; Mohammed, R.; Hetta, M.; Abdelaziz, T.; El-Gendy, A.; Sleim, M. Biological and chemical investigation of the soft coral *Lobophytum pauciflorum* collected from the Egyptian red sea. *International Journal of Pharmacognosy and Phytochemical Research* **2016**, *8*, 906-911.
66. Su, Y.D.; Sung, C.S.; Wen, Z.H.; Chen, Y.H.; Chang, Y.C.; Chen, J.J.; Fang, L.S.; Wu, Y.C.; Sheu, J.H.; Sung, P.J. New 9-hydroxybriarane diterpenoids from a Gorgonian coral Briareum sp. (Briareidae). *Int. J. Mol. Sci.* **2016**, *17*, doi:10.3390/ijms17010079.
67. Tseng, W.R.; Huang, C.Y.; Tsai, Y.Y.; Lin, Y.S.; Hwang, T.L.; Su, J.H.; Sung, P.J.; Dai, C.F.; Sheu, J.H. New cytotoxic and anti-inflammatory steroids from the soft coral *Klyxum flaccidum*. *Bioorg. Med. Chem. Lett.* **2016**, *26*, 3253-3257, doi:10.1016/j.bmcl.2016.05.060.
68. Yuan, W.; Cheng, S.; Fu, W.; Zhao, M.; Li, X.; Cai, Y.; Dong, J.; Huang, K.; Gustafson, K.R.; Yan, P. Structurally diverse metabolites from the soft coral *Sinularia verruca* collected in the South China sea. *J. Nat. Prod.* **2016**, *79*, 1124-1131, doi:10.1021/acs.jnatprod.6b00031.
69. Zhao, M.; Cheng, S.; Yuan, W.; Xi, Y.; Li, X.; Dong, J.; Huang, K.; Gustafson, K.R.; Yan, P. Cembranoids from a Chinese collection of the soft coral *Lobophytum crassum*. *Mar. Drugs* **2016**, *14*, doi:10.3390/md14060111.
70. Chao, C.H.; Wu, C.Y.; Huang, C.Y.; Wang, H.C.; Dai, C.F.; Wu, Y.C.; Sheu, J.H. Cubitanoids and cembranoids from the soft coral *Sinularia nanolobata*. *Mar. Drugs* **2016**, *14*, doi:10.3390/md14080150.
71. Huang, C.Y.; Ahmed, A.F.; Su, J.H.; Sung, P.J.; Hwang, T.L.; Chiang, P.L.; Dai, C.F.; Liaw, C.C.; Sheu, J.H. Bioactive new withanolides from the cultured soft coral *Sinularia brassica*. *Bioorg. Med. Chem. Lett.* **2017**, *27*, 3267-3271, doi:10.1016/j.bmcl.2017.06.029.
72. Li, W.; Zou, Y.H.; Ge, M.X.; Lou, L.L.; Xu, Y.S.; Ahmed, A.; Chen, Y.Y.; Zhang, J.S.; Tang, G.H.; Yin, S. Biscembranoids and cembranoids from the soft coral *Sarcophyton elegans*. *Mar. Drugs* **2017**, *15*, doi:10.3390/md15040085.
73. Ahmed, A.F.; Teng, W.T.; Huang, C.Y.; Dai, C.F.; Hwang, T.L.; Sheu, J.H. Anti-inflammatory lobane and prenyleudesmane diterpenoids from the soft coral *Lobophytum varium*. *Mar. Drugs* **2017**, *15*, doi:10.3390/md15100300.

74. Wu, Q.; Li, X.W.; Li, H.; Yao, L.G.; Tang, W.; Miao, Z.H.; Wang, H.; Guo, Y.W. Bioactive polyoxygenated cembranoids from a novel Hainan chemotype of the soft coral *Sinularia flexibilis*. *Bioorg. Med. Chem. Lett.* **2019**, *29*, 185-188, doi:10.1016/j.bmcl.2018.12.004.
75. Liu, M.; Qi, C.; Sun, W.; Shen, L.; Wang, J.; Liu, J.; Lai, Y.; Xue, Y.; Hu, Z.; Zhang, Y. α-Glucosidase inhibitors from the coral-associated fungus *Aspergillus terreus*. *Front. Chem.* **2018**, *6*, 422, doi:10.3389/fchem.2018.00422.
76. Huang, C.Y.; Tseng, W.R.; Ahmed, A.F.; Chiang, P.L.; Tai, C.J.; Hwang, T.L.; Dai, C.F.; Sheu, J.H. Anti-inflammatory polyoxygenated steroids from the soft coral *Lobophytum michaelae*. *Mar. Drugs* **2018**, *16*, doi:10.3390/md16030093.
77. Wu, C.H.; Chao, C.H.; Huang, T.Z.; Huang, C.Y.; Hwang, T.L.; Dai, C.F.; Sheu, J.H. Cembranoid-related metabolites and biological activities from the soft coral *Sinularia flexibilis*. *Mar. Drugs* **2018**, *16*, doi:10.3390/md16080278.
78. Januar, H.I.; Chasanah, E.; Motti, C.A.; Tapiolas, D.M.; Liptrot, C.H.; Wright, A.D. Cytotoxic cembranes from Indonesian specimens of the soft coral *Nephthea* sp. *Mar. Drugs* **2010**, *8*, 2142-2152, doi:10.3390/md8072142.
79. Nguyen, H.T.; Chau, V.M.; Phan, V.K.; Hoang, T.H.; Nguyen, H.N.; Nguyen, X.C.; Tran, H.Q.; Nguyen, X.N.; Hyun, J.H.; Kang, H.K., et al. Chemical components from the Vietnamese soft coral *Lobophytum* sp. *Arch. Pharm. Res.* **2010**, *33*, 503-508, doi:10.1007/s12272-010-0402-3.
80. Chen, B.-W.; Su, J.-H.; Dai, C.-F.; Sung, P.-J.; Wu, Y.-C.; Lin, Y.-T.; Sheu, J.-H. Two new cembranes from a Formosan soft coral *Sinularia facile*. *Bull. Chem. Soc. Jpn.* **2011**, *84*, 1371-1373, doi:10.1246/bcsj.20110186.
81. Chau, V.M.; Phan, V.K.; Nguyen, X.; Nguyen, X.C.; Nguyen, P.T.; Nguyen, H.N.; Hoang le, T.A.; Do, C.T.; Thuy, D.T.; Kang, H.K., et al. Cytotoxic and antioxidant activities of diterpenes and sterols from the Vietnamese soft coral *Lobophytum compactum*. *Bioorg. Med. Chem. Lett.* **2011**, *21*, 2155-2159, doi:10.1016/j.bmcl.2011.01.072.
82. Su, C.C.; Su, J.H.; Lin, J.J.; Chen, C.C.; Hwang, W.I.; Huang, H.H.; Wu, Y.J. An investigation into the cytotoxic effects of 13-acetoxysarcocrassolide from the soft coral *Sarcophyton crassocaule* on bladder cancer cells. *Mar. Drugs* **2011**, *9*, 2622-2642, doi:10.3390/md9122622.
83. Hegazy, M.-E.F.; El-Beih, A.A.; Moustafa, A.Y.; Hamdy, A.A.; Alhammady, M.A.; Selim, R.M.; Abdel-Rehim, M.; Paré, P.W. Cytotoxic Cembranoids from the Red Sea Soft Coral *Sarcophyton glaucum*. *Nat. Prod. Commun.* **2011**, *6*, 1934578X1100601, doi:10.1177/1934578x1100601205.
84. LIAO, X.-j.; TANG, L.-d.; LIANG, Y.-w.; GENG, H.-w.; XU, S.-h. Isolation and identification of two new polyhydroxylated sterols from soft coral *Sinularia* sp. *Chem. Res. Chinese U.* **2011**, *27*, 217-220.
85. Lee, N.L.; Su, J.H. Tetrahydrofuran cembranoids from the cultured soft coral Lobophytum crassum. *Mar. Drugs* **2011**, *9*, 2526-2536, doi:10.3390/md9122526.
86. Cheng, S.Y.; Huang, K.J.; Wang, S.K.; Duh, C.Y. Capilloquinol: a novel farnesyl quinol from the Dongsha atoll soft coral *Sinularia capillosa*. *Mar. Drugs* **2011**, *9*, 1469-1476, doi:10.3390/md9091469.
87. Quang, T.H.; Ha, T.T.; Minh, C.V.; Kiem, P.V.; Huong, H.T.; Ngan, N.T.; Nhiem, N.X.; Tung, N.H.; Thao, N.P.; Thuy, D.T., et al. Cytotoxic and PPARs transcriptional activities of sterols from the Vietnamese soft coral *Lobophytum laevigatum*. *Bioorg. Med. Chem. Lett.* **2011**, *21*, 2845-2849, doi:10.1016/j.bmcl.2011.03.089.
88. Lin, Y.S.; Eid Fazary, A.; Chen, C.H.; Kuo, Y.H.; Shen, Y.C. Bioactive xenicane diterpenoids from the Taiwanese soft coral *Asterospicularia laurae*. *Chem. Biodivers.* **2011**, *8*, 1310-1317.
89. Elshamy, A.I.; Abdel-Razik, A.F.; Nassar, M.I.; Mohamed, T.K.; Ibrahim, M.A.; El-Kousy, S.M. A new gorgostane derivative from the Egyptian Red Sea soft coral *Heteroxenia ghardaqensis*. *Nat. Prod. Res.* **2013**, *27*, 1250-1254, doi:10.1080/14786419.2012.724417.
90. Shi, H.; Yu, S.; Liu, D.; van Ofwegen, L.; Proksch, P.; Lin, W. Sinularones A-I, new cyclopentenone and butenolide derivatives from a marine soft coral *Sinularia* sp. and their antifouling activity. *Mar. Drugs* **2012**, *10*, 1331-1344, doi:10.3390/md10061331.
91. Hegazy, M.E.; Gamal Eldeen, A.M.; Shahat, A.A.; Abdel-Latif, F.F.; Mohamed, T.A.; Whittlesey, B.R.; Pare, P.W. Bioactive hydroperoxyl cembranoids from the Red Sea soft coral *Sarcophyton glaucum*. *Mar. Drugs* **2012**, *10*, 209-222, doi:10.3390/md10010209.
92. Roy, P.K.; Maarisit, W.; Roy, M.C.; Taira, J.; Ueda, K. Five new diterpenoids from an Okinawan soft coral, *Cespitularia* sp. *Mar. Drugs* **2012**, *10*, 2741-2748, doi:10.3390/md10122741.

93. Li, R.; Shao, C.L.; Qi, X.; Li, X.B.; Li, J.; Sun, L.L.; Wang, C.Y. Polyoxygenated sterols from the South China Sea soft coral *Sinularia* sp. *Mar. Drugs* **2012**, *10*, 1422-1432, doi:10.3390/md10071422.
94. Wang, S.K.; Duh, C.Y. New cytotoxic cembranolides from the soft coral *Lobophytum michaelae*. *Mar. Drugs* **2012**, *10*, 306-318, doi:10.3390/md10020306.
95. Wang, S.K.; Hsieh, M.K.; Duh, C.Y. Three new cembranoids from the Taiwanese soft coral *Sarcophyton ehrenbergi*. *Mar. Drugs* **2012**, *10*, 1433-1444, doi:10.3390/md10071433.
96. Wang, S.K.; Puu, S.Y.; Duh, C.Y. New 19-oxygenated steroids from the soft coral Nephthea chabrolii. *Mar. Drugs* **2012**, *10*, 1288-1296, doi:10.3390/md10061288.
97. Chen, S.-P.; Chen, B.-W.; Dai, C.-F.; Sung, P.-J.; Wu, Y.-C.; Sheu, J.-H. Sarcophytonins F and G, new dihydrofuranocembranoids from a Dongsha Atoll soft coral *Sarcophyton* sp. *Bull. Chem. Soc. Jpn.* **2012**, *85*, 920-922, doi:10.1246/bcsj.20120100.
98. Govindam, S.V.; Yoshioka, Y.; Kanamoto, A.; Fujiwara, T.; Okamoto, T.; Ojika, M. Cyclolobatriene, a novel prenylated germacrene diterpene, from the soft coral *Lobophytum pauciflorum*. *Bioorg. Med. Chem.* **2012**, *20*, 687-692, doi:10.1016/j.bmc.2011.12.012.
99. Alarif, W.M.; Abdel-lateff, A.; Al-lihaibi, S.S.; Ayyad, S.-E.N.; Badria, F.A.; Alsofyani, A.A.; Abou-Elnaga, Z.S. Marine bioactive steryl esters from the Red Sea black coral *Antipathes dichotoma*. *CLEAN - Soil, Air, Water* **2013**, *41*, 1116-1121, doi:10.1002/clen.201200409.
100. Yen, W.H.; Hu, L.C.; Su, J.H.; Lu, M.C.; Twan, W.H.; Yang, S.Y.; Kuo, Y.C.; Weng, C.F.; Lee, C.H.; Kuo, Y.H., et al. Norcembranoidal diterpenes from a Formosan soft coral *Sinularia* sp. *Molecules* **2012**, *17*, 14058-14066, doi:10.3390/molecules171214058.
101. Su, C.C.; Wong, B.S.; Chin, C.; Wu, Y.J.; Su, J.H. Oxygenated cembranoids from the soft coral *Sinularia flexibilis*. *Int. J. Mol. Sci.* **2013**, *14*, 4317-4325, doi:10.3390/ijms14024317.
102. Huang, C.Y.; Liaw, C.C.; Chen, B.W.; Chen, P.C.; Su, J.H.; Sung, P.J.; Dai, C.F.; Chiang, M.Y.; Sheu, J.H. Withanolide-based steroids from the cultured soft coral *Sinularia brassica*. *J. Nat. Prod.* **2013**, *76*, 1902-1908, doi:10.1021/np400454q.
103. Aboutabl el, S.A.; Azzam, S.M.; Michel, C.G.; Selim, N.M.; Hegazy, M.F.; Ali, A.H.; Hussein, A.A. Bioactive terpenoids from the Red Sea soft coral *Sinularia polydactyla*. *Nat. Prod. Res.* **2013**, *27*, 2224-2226, doi:10.1080/14786419.2013.805333.
104. Zhang, J.; Li, L.-C.; Wang, K.-L.; Liao, X.-J.; Deng, Z.; Xu, S.-H. Pentacyclic hemiacetal sterol with antifouling and cytotoxic activities from the soft coral *Nephthea* sp. *Bioorg. Med. Chem. Lett.* **2013**, *23*, 1079-1082, doi:https://doi.org/10.1016/j.bmcl.2012.12.012.
105. Gong, K.K.; Tang, X.L.; Zhang, G.; Cheng, C.L.; Zhang, X.W.; Li, P.L.; Li, G.Q. Polyhydroxylated steroids from the South China Sea soft coral *Sarcophyton* sp. and their cytotoxic and antiviral activities. *Mar. Drugs* **2013**, *11*, 4788-4798, doi:10.3390/md11124788.
106. Zhao, M.; Yin, J.; Jiang, W.; Ma, M.; Lei, X.; Xiang, Z.; Dong, J.; Huang, K.; Yan, P. Cytotoxic and antibacterial cembranoids from a South China Sea soft coral, *Lobophytum* sp. *Mar. Drugs* **2013**, *11*, 1162-1172, doi:10.3390/md11041162.
107. Ellithey, M.S.; Lall, N.; Hussein, A.A.; Meyer, D. Cytotoxic, cytostatic and HIV-1 PR inhibitory activities of the soft coral *Litophyton arboreum*. *Mar. Drugs* **2013**, *11*, 4917-4936, doi:10.3390/md11124917.
108. Rajaram, S.; Ramulu, U.; Ramesh, D.; Srikanth, D.; Bhattacharya, P.; Prabhakar, P.; Kalivendi, S.V.; Babu, K.S.; Venkateswarlu, Y.; Navath, S. Anti-cancer evaluation of carboxamides of furano-sesquiterpene carboxylic acids from the soft coral *Sinularia kavarattiensis*. *Bioorg. Med. Chem. Lett.* **2013**, *23*, 6234-6238, doi:10.1016/j.bmcl.2013.09.093.
109. Tsai, T.C.; Wu, Y.J.; Su, J.H.; Lin, W.T.; Lin, Y.S. A new spatane diterpenoid from the cultured soft coral *Sinularia leptoclados*. *Mar. Drugs* **2013**, *11*, 114-123, doi:10.3390/md11010114.
110. Yen, W.H.; Chen, W.F.; Cheng, C.H.; Dai, C.F.; Lu, M.C.; Su, J.H.; Su, Y.D.; Chen, Y.H.; Chang, Y.C.; Chen, Y.H., et al. A new $5\alpha,8\alpha$-epidioxysterol from the soft coral *Sinularia gaweli*. *Molecules* **2013**, *18*, 2895-2903, doi:10.3390/molecules18032895.
111. Tseng, Y.J.; Wang, S.K.; Duh, C.Y. Secosteroids and norcembranoids from the soft coral *Sinularia nanolobata*. *Mar. Drugs* **2013**, *11*, 3288-3296, doi:10.3390/md11093288.

112. Cai, Y.-S.; Yao, L.-G.; Di Pascale, A.; Irace, C.; Mollo, E.; Taglialatela-Scafati, O.; Guo, Y.-W. Polyoxygenated diterpenoids of the eunicellin-type from the Chinese soft coral *Cladiella krempfi*. *Tetrahedron* **2013**, *69*, 2214-2219, doi:10.1016/j.tet.2012.12.051.
113. Xio, Y.J.; Su, J.H.; Chen, B.W.; Tseng, Y.J.; Wu, Y.C.; Sheu, J.H. Oxygenated ylangene-derived sesquiterpenoids from the soft coral *Lemnalia philippinensis*. *Mar. Drugs* **2013**, *11*, 3735-3741, doi:10.3390/md11103735.
114. Elkhateeb, A.; El-Beih, A.A.; Gamal-Eldeen, A.M.; Alhammady, M.A.; Ohta, S.; Pare, P.W.; Hegazy, M.E. New terpenes from the Egyptian soft coral *Sarcophyton ehrenbergi*. *Mar. Drugs* **2014**, *12*, 1977-1986, doi:10.3390/md12041977.
115. Kuo, C.Y.; Juan, Y.S.; Lu, M.C.; Chiang, M.Y.; Dai, C.F.; Wu, Y.C.; Sung, P.J. Pregnane-type steroids from the Formosan soft coral *Scleronephthya flexilis*. *Int. J. Mol. Sci.* **2014**, *15*, 10136-10149, doi:10.3390/ijms150610136.
116. Elkhayat, E.S.; Ibrahim, S.R.M.; Fouad, M.A.; Mohamed, G.A. Dendronephthols A–C, new sesquiterpenoids from the Red Sea soft coral *Dendronephthya* sp. *Tetrahedron* **2014**, *70*, 3822-3825, doi:10.1016/j.tet.2014.03.056.
117. Al-Footy, K.O.; Alarif, W.M.; Asiri, F.; Aly, M.M.; Ayyad, S.-E.N. Rare pyrane-based cembranoids from the Red Sea soft coral *Sarcophyton trocheliophorum* as potential antimicrobial–antitumor agents. *Med. Chem. Res.* **2014**, *24*, 505-512, doi:10.1007/s00044-014-1147-1.
118. Lei, L.-F.; Chen, M.-F.; Wang, T.; He, X.-X.; Liu, B.-X.; Deng, Y.; Chen, X.-J.; Li, Y.-T.; Guan, S.-Y.; Yao, J.-H., et al. Novel cytotoxic nine-membered macrocyclic polysulfur cembranoid lactones from the soft coral *Sinularia* sp. *Tetrahedron* **2014**, *70*, 6851-6858, doi:10.1016/j.tet.2014.07.042.
119. Eltahawy, N.A.; Ibrahim, A.K.; Radwan, M.M.; ElSohly, M.A.; Hassanean, H.A.; Ahmed, S.A. Cytotoxic cembranoids from the Red Sea soft coral, *Sarcophyton auritum*. *Tetrahedron Lett.* **2014**, *55*, 3984-3988, doi:10.1016/j.tetlet.2014.05.013.
120. Roy, P.K.; Roy, M.C.; Taira, J.; Ueda, K. Structure and bioactivity of a trisnorditerpenoid and a diterpenoid from an Okinawan soft coral, *Cespitularia* sp. *Tetrahedron Lett.* **2014**, *55*, 1421-1423, doi:10.1016/j.tetlet.2014.01.035.
121. Cheng, S.Y.; Shih, N.L.; Hou, K.Y.; Ger, M.J.; Yang, C.N.; Wang, S.K.; Duh, C.Y. Kelsoenethiol and dikelsoenyl ether, two unique kelsoane-type sesquiterpenes, from the Formosan soft coral *Nephthea erecta*. *Bioorg. Med. Chem. Lett.* **2014**, *24*, 473-475, doi:10.1016/j.bmcl.2013.12.037.
122. Cheng, S.Y.; Shih, N.L.; Chuang, C.T.; Chiou, S.F.; Yang, C.N.; Wang, S.K.; Duh, C.Y. Sinugyrosanolide A, an unprecedented C-4 norcembranoid, from the Formosan soft coral *Sinularia gyrosa*. *Bioorg. Med. Chem. Lett.* **2014**, *24*, 1562-1564, doi:10.1016/j.bmcl.2014.01.073.
123. Al-Lihaibi, S.S.; Alarif, W.M.; Abdel-Lateff, A.; Ayyad, S.E.; Abdel-Naim, A.B.; El-Senduny, F.F.; Badria, F.A. Three new cembranoid-type diterpenes from Red Sea soft coral *Sarcophyton glaucum*: isolation and antiproliferative activity against HepG2 cells. *Eur. J. Med. Chem.* **2014**, *81*, 314-322, doi:10.1016/j.ejmech.2014.05.016.
124. Tseng, Y.J.; Yang, Y.C.; Wang, S.K.; Duh, C.Y. Numerosol A-D, new cembranoid diterpenes from the soft coral *Sinularia numerosa*. *Mar. Drugs* **2014**, *12*, 3371-3380, doi:10.3390/md12063371.
125. Lin, Y.C.; Wang, S.S.; Chen, C.H.; Kuo, Y.H.; Shen, Y.C. Cespitulones A and B, cytotoxic diterpenoids of a new structure class from the soft coral *Cespitularia taeniata*. *Mar. Drugs* **2014**, *12*, 3477-3486, doi:10.3390/md12063477.
126. Ghandourah, M.A.; Alarif, W.M.; Abdel-Lateff, A.; Al-Lihaibi, S.S.; Ayyad, S.-E.N.; Basaif, S.A.; Badria, F.A. Two new terpenoidal derivatives: a himachalene-type sesquiterpene and 13,14-secosteroid from the soft coral *Litophyton arboreum*. *Med. Chem. Res.* **2015**, *24*, 4070-4077, doi:10.1007/s00044-015-1456-z.
127. Zhang, N.X.; Tang, X.L.; van Ofwegen, L.; Xue, L.; Song, W.J.; Li, P.L.; Li, G.Q. Cyclopentenone derivatives and polyhydroxylated steroids from the soft coral *Sinularia acuta*. *Chem. Biodivers.* **2015**, *12*, 273-283.
128. Nam, N.H.; Tung, P.T.; Ngoc, N.T.; Hanh, T.T.H.; Thao, N.P.; Thanh, N.V.; Cuong, N.X.; Thao, D.T.; Huong, T.T.; Thung, D.C., et al. Cytotoxic biscembranoids from the soft coral *Sarcophyton pauciplicatum*. *Chem. Pharm. Bull.* **2015**, *63*, 636–640.
129. Cheng, S.Y.; Wang, S.K.; Hsieh, M.K.; Duh, C.Y. Polyoxygenated cembrane diterpenoids from the soft coral *Sarcophyton ehrenbergi*. *Int. J. Mol. Sci.* **2015**, *16*, 6140-6152, doi:10.3390/ijms16036140.

130. Chen, W.-T.; Li, J.; Wang, J.-R.; Li, X.-W.; Guo, Y.-W. Structural diversity of terpenoids in the soft coral *Sinularia flexibilis*, evidenced by a collection from the South China Sea. *RSC Adv.* **2015**, *5*, 23973-23980.
131. Al-Footy, K.O.; Alarif, W.M.; Zubair, M.S.; Ghandourah, M.A.; Aly, M.M. Antibacterial and cytotoxic properties of isoprenoids from the red sea soft coral, *Lobophytum* sp. *Tropical Journal of Pharmaceutical Research* **2016**, *15*, 1431, doi:10.4314/tjpr.v15i7.11.
132. Koncic, M.Z.; Ioannou, E.; Sawadogo, W.R.; Abdel-Razik, A.F.; Vagias, C.; Diederich, M.; Roussis, V. 4alpha-methylated steroids with cytotoxic activity from the soft coral *Litophyton mollis*. *Steroids* **2016**, *115*, 130-135, doi:10.1016/j.steroids.2016.08.017.
133. Xu, M.W.; Hao, Y.T. A new sesquiterpene from the South China Sea gorgonian coral *Subergorgia suberosa*. *Nat. Prod. Res.* **2016**, *30*, 2402-2406, doi:10.1080/14786419.2016.1190720.
134. Ngoc, N.T.; Huong, P.T.M.; Thanh, N.V.; Cuong, N.X.; Nam, N.H.; Thung, D.C.; Kiem, P.V.; Minh, C.V. Steroid constituents from the soft coral *Sinularia nanolobata*. *Chem. Pharm. Bull.* **2016**, *64*, 1417–1419.
135. Tsai, T.C.; Huang, Y.T.; Chou, S.K.; Shih, M.C.; Chiang, C.Y.; Su, J.H. Cytotoxic oxygenated steroids from the soft coral *Nephthea erecta*. *Chem. Pharm. Bull.* **2016**, *64*, 1519–1522.
136. Trifman, Y.J.; Aknin, M.; Gauvin-Bialecki, A.; Benayahu, Y.; Carmeli, S.; Kashman, Y. Bisdioxycalamenene: a bis-sesquiterpene from the soft coral *Rhytisma fulvum fulvum*. *Mar. Drugs* **2016**, *14*, doi:10.3390/md14020041.
137. Urda, C.; Fernandez, R.; Perez, M.; Rodriguez, J.; Jimenez, C.; Cuevas, C. Protoxenicins A and B, cytotoxic long-chain acylated Xenicanes from the soft coral *Protodendron repens*. *J. Nat. Prod.* **2017**, *80*, 713-719, doi:10.1021/acs.jnatprod.7b00046.
138. Chao, C.H.; Li, W.L.; Huang, C.Y.; Ahmed, A.F.; Dai, C.F.; Wu, Y.C.; Lu, M.C.; Liaw, C.C.; Sheu, J.H. Isoprenoids from the soft coral *Sarcophyton glaucum*. *Mar. Drugs* **2017**, *15*, doi:10.3390/md15070202.
139. Zhao, M.; He, T.; Shi, S.K.; Song, L.X.; Lu, Y. A new dolabellane diterpene and a new polyacetylene from the soft coral *Clavularia viridis*. *Nat. Prod. Res.* **2018**, *32*, 1104-1108, doi:10.1080/14786419.2017.1380025.
140. Mohammed, R.; Radwan, M.M.; Ma, G.; Mohamed, T.A.; Seliem, M.A.; Thabet, M.; ElSohly, M.A. Bioactive sterols and sesquiterpenes from the Red Sea soft coral *Sinularia terspilli*. *Med. Chem. Res.* **2017**, *26*, 1647-1652, doi:10.1007/s00044-017-1876-z.
141. Gao, Y.; Xiao, W.; Liu, H.-C.; Wang, J.-R.; Yao, L.-G.; Ouyang, P.-K.; Wang, D.-C.; Guo, Y.-W. Clavirolide G, a new rare dolabellane-type diterpenoid from the Xisha soft coral *Clavularia viridis*. *Chin. Chem. Lett.* **2017**, *28*, 905-908, doi:10.1016/j.cclet.2017.01.004.
142. Sun, P.; Meng, L.Y.; Tang, H.; Liu, B.S.; Li, L.; Yi, Y.; Zhang, W. Sinularosides A and B, bioactive 9,11-secosteroidal glycosides from the South China Sea soft coral *Sinularia humilis* Ofwegen. *J. Nat. Prod.* **2012**, *75*, 1656-1659, doi:10.1021/np300475d.
143. Liang, L.F.; Wang, X.J.; Zhang, H.Y.; Liu, H.L.; Li, J.; Lan, L.F.; Zhang, W.; Guo, Y.W. Bioactive polyhydroxylated steroids from the Hainan soft coral *Sinularia depressa* Tixier-Durivault. *Bioorg. Med. Chem. Lett.* **2013**, *23*, 1334-1337, doi:10.1016/j.bmcl.2012.12.087.
144. Liang, L.-F.; Lan, L.-F.; Taglialatela-Scafati, O.; Guo, Y.-W. Sartrolides A–G and bissartrolide, new cembranolides from the South China Sea soft coral *Sarcophyton trocheliophorum* Marenzeller. *Tetrahedron* **2013**, *69*, 7381-7386, doi:10.1016/j.tet.2013.06.068.
145. Abou El-Ezz, R.F.; Ahmed, S.A.; Radwan, M.M.; Ayoub, N.A.; Afifi, M.S.; Ross, S.A.; Szymanski, P.T.; Fahmy, H.; Khalifa, S.I. Bioactive cembranoids from the Red Sea soft coral *Sarcophyton glaucum*. *Tetrahedron Lett.* **2013**, *54*, 989-992, doi:10.1016/j.tetlet.2012.12.037.
146. Wang, Z.; Tang, H.; Wang, P.; Gong, W.; Xue, M.; Zhang, H.; Liu, T.; Liu, B.; Yi, Y.; Zhang, W. Bioactive polyoxygenated steroids from the South China sea soft coral, *Sarcophyton* sp. *Mar. Drugs* **2013**, *11*, 775-787, doi:10.3390/md11030775.
147. Elshamy, A.I.; Nassar, M.I.; Mohamed, T.K.; Madkour, H.A. A new hydroxymethyl diacylglycerol from methanol extract of Egyptian soft coral *Heteroxenia ghardaqensis*. *Journal of Biologically Active Products from Nature* **2015**, *5*, 172-177, doi:10.1080/22311866.2015.1090339.

148. Rajaram, S.; Ramesh, D.; Ramulu, U.; Anjum, M.; Kumar, P.; Murthy, U.; Hussain, M.A.; Sastry, G.N.; Venkateswarlu, Y. Chemical examination of the soft coral *Sinularia kavarattiensis* and evaluation of anti-microbial activity. *Indian J. Chem.* **2014**, *53B*, 1086-1090.
149. Suna, X.P.; Cao, F.; Shao, C.L.; Wang, M.; Zhang, X.L.; Wang, C.Y. Antibacterial Δ^1-3-ketosteroids from the South China Sea Gorgonian coral *Subergorgia rubra*. *Chem. Biodivers.* **2015**, *12*, 1068-1074.
150. Angulo-Preckler, C.; Genta-Jouve, G.; Mahajan, N.; de la Cruz, M.; de Pedro, N.; Reyes, F.; Iken, K.; Avila, C.; Thomas, O.P. Gersemiols A–C and eunicellol A, diterpenoids from the Arctic soft coral *Gersemia fruticosa*. *J. Nat. Prod.* **2016**, *79*, 1132-1136, doi:10.1021/acs.jnatprod.6b00040.
151. Phan, C.S.; Vairappan, C.S. Capgermacrenes D-G, new sesquiterpenoids from a Bornean soft coral, *Capnella imbricata*. *Nat. Prod. Res.* **2017**, *31*, 742-748, doi:10.1080/14786419.2016.1241997.
152. Zubair, M.; Alarif, W.; Al-Footy, K.; Ph, M.; Ali, M.; Basaif, S.; Al-Lihaibi, S.; Ayyad, S.-E. New antimicrobial biscembrane hydrocarbon and cembranoid diterpenes from the soft coral *Sarcophyton trocheliophorum*. *Turk. J. Chem.* **2016**, *40*, 385-392, doi:10.3906/kim-1502-82.
153. Tani, K.; Kamada, T.; Phan, C.S.; Vairappan, C.S. New cembrane-type diterpenoids from Bornean soft coral *Nephthea* sp. with antifungal activity against *Lagenidium thermophilum*. *Nat. Prod. Res.* **2018**, 10.1080/14786419.2018.1475387, 1-7, doi:10.1080/14786419.2018.1475387.
154. Wang, S.K.; Hsieh, M.K.; Duh, C.Y. New diterpenoids from soft coral Sarcophyton ehrenbergi. *Mar. Drugs* **2013**, *11*, 4318-4327, doi:10.3390/md11114318.
155. Cheng, S.Y.; Wang, S.K.; Duh, C.Y. Secocrassumol, a seco-cembranoid from the Dongsha Atoll soft coral *Lobophytum crassum*. *Mar. Drugs* **2014**, *12*, 6028-6037, doi:10.3390/md12126028.
156. Cheng, W.; Ren, J.; Huang, Q.; Long, H.; Jin, H.; Zhang, L.; Liu, H.; van Ofwegen, L.; Lin, W. Pregnane steroids from a gorgonian coral *Subergorgia suberosa* with anti-flu virus effects. *Steroids* **2016**, *108*, 99-104, doi:10.1016/j.steroids.2016.02.003.
157. Lai, D.; Geng, Z.; Deng, Z.; van Ofwegen, L.; Proksch, P.; Lin, W. Cembranoids from the soft coral *Sinularia rigida* with antifouling activities. *J. Agric. Food Chem.* **2013**, *61*, 4585-4592, doi:10.1021/jf401303q.
158. Zhang, J.; Liang, Y.; Wang, K.L.; Liao, X.J.; Deng, Z.; Xu, S.H. Antifouling steroids from the South China Sea gorgonian coral *Subergorgia suberosa*. *Steroids* **2014**, *79*, 1-6, doi:10.1016/j.steroids.2013.10.007.
159. Wang, J.; Su, P.; Gu, Q.; Li, W.D.; Guo, J.L.; Qiao, W.; Feng, D.Q.; Tang, S.A. Antifouling activity against bryozoan and barnacle by cembrane diterpenes from the soft coral *Sinularia flexibilis*. *Int. Biodeterior. Biodegrad.* **2017**, *120*, 97-103, doi:10.1016/j.ibiod.2017.02.013.
160. Bonnard, I.; Jhaumeer-Laulloo, S.B.; Bontemps, N.; Banaigs, B.; Aknin, M. New lobane and cembrane diterpenes from two comorian soft corals. *Mar. Drugs* **2010**, *8*, 359-372, doi:10.3390/md8020359.
161. Putra, M.Y.; Murniasih, T.; Swasono, R.T.; Wibowo, J.T.; Saputri, A.N.C.; Widhiana, M.R.; Arlyza, I.S. Secondary metabolites and their biological activities in Indonesian soft coral of the genus *Lobophytum*. *Asian Pac. J. Trop. Biomed.* **2016**, *6*, 909-913, doi:10.1016/j.apjtb.2016.08.011.
162. Liang, L.-F.; Kurtán, T.; Mándi, A.; Gao, L.-X.; Li, J.; Zhang, W.; Guo, Y.-W. Sarsolenane and capnosane diterpenes from the Hainan soft coral *Sarcophyton trocheliophorum* Marenzeller as PTP1B Inhibitors. *Eur. J. Org. Chem.* **2014**, *2014*, 1841-1847, doi:10.1002/ejoc.201301683.
163. Ye, F.; Zhu, Z.D.; Gu, Y.C.; Li, J.; Zhu, W.L.; Guo, Y.W. Further new diterpenoids as PTP1B inhibitors from the Xisha soft coral *Sinularia polydactyla*. *Mar. Drugs* **2018**, *16*, doi:10.3390/md16040103.
164. Kodani, S.; Sato, K.; Higuchi, T.; Casareto, B.E.; Suzuki, Y. Montiporic acid D, a new polyacetylene carboxylic acid from scleractinian coral *Montipora digitata*. *Nat. Prod. Res.* **2013**, *27*, 1859-1862, doi:10.1080/14786419.2013.768992.
165. Liu, M.; Zhou, Q.; Wang, J.; Liu, J.; Qi, C.; Lai, Y.; Zhu, H.; Xue, Y.; Hu, Z.; Zhang, Y. Anti-inflammatory butenolide derivatives from the coral-derived fungus *Aspergillus terreus* and structure revisions of aspernolides D and G, butyrolactone VI and 4′,8′′-diacetoxy butyrolactone VI. *RSC Adv.* **2018**, *8*, 13040-13047, doi:10.1039/c8ra01840e.

166. Zheng, C.J.; Shao, C.L.; Guo, Z.Y.; Chen, J.F.; Deng, D.S.; Yang, K.L.; Chen, Y.Y.; Fu, X.M.; She, Z.G.; Lin, Y.C., et al. Bioactive hydroanthraquinones and anthraquinone dimers from a soft coral-derived *Alternaria* sp. fungus. *J. Nat. Prod.* **2012**, *75*, 189-197, doi:10.1021/np200766d.
167. Li, H.J.; Xie, Y.L.; Xie, Z.L.; Chen, Y.; Lam, C.K.; Lan, W.J. Chondrosterins A-E, triquinane-type sesquiterpenoids from soft coral-associated fungus *Chondrostereum* sp. *Mar. Drugs* **2012**, *10*, 627-638, doi:10.3390/md10030627.
168. Fu, P.; Kong, F.; Wang, Y.; Wang, Y.; Liu, P.; Zuo, G.; Zhu, W. Antibiotic metabolites from the coral-associated Actinomycete *Streptomyces* sp. OUCMDZ-1703. *Chin. J. Chem .* **2013**, *31*, 100-104, doi:10.1002/cjoc.201201062.
169. Wang, W.; Liao, Y.; Tang, C.; Huang, X.; Luo, Z.; Chen, J.; Cai, P. Cytotoxic and antibacterial compounds from the coral-derived fungus *Aspergillus tritici* SP2-8-1. *Mar. Drugs* **2017**, *15*, doi:10.3390/md15110348.
170. Zhuang, Y.; Teng, X.; Wang, Y.; Liu, P.; Li, G.; Zhu, W. New quinazolinone alkaloids within rare amino acid residue from coral-associated fungus, *Aspergillus* versicolor LCJ-5-4. *Org. Lett.* **2011**, *13*, 1130-1133.
171. Wei, M.Y.; Li, D.; Shao, C.L.; Deng, D.S.; Wang, C.Y. (±)-Pestalachloride D, an antibacterial racemate of chlorinated benzophenone derivative from a soft coral-derived fungus *Pestalotiopsis* sp. *Mar. Drugs* **2013**, *11*, 1050-1060, doi:10.3390/md11041050.
172. Bao, J.; Sun, Y.L.; Zhang, X.Y.; Han, Z.; Gao, H.C.; He, F.; Qian, P.Y.; Qi, S.H. Antifouling and antibacterial polyketides from marine gorgonian coral-associated fungus *Penicillium* sp. SCSGAF 0023. *J. Antibiot. (Tokyo)* **2013**, *66*, 219-223, doi:10.1038/ja.2012.110.
173. Zheng, C.J.; Shao, C.L.; Wu, L.Y.; Chen, M.; Wang, K.L.; Zhao, D.L.; Sun, X.P.; Chen, G.Y.; Wang, C.Y. Bioactive phenylalanine derivatives and cytochalasins from the soft coral-derived fungus, *Aspergillus elegans*. *Mar. Drugs* **2013**, *11*, 2054-2068, doi:10.3390/md11062054.
174. Shao, C.L.; Xu, R.F.; Wang, C.Y.; Qian, P.Y.; Wang, K.L.; Wei, M.Y. Potent antifouling marine dihydroquinolin-2(1H)-one-containing alkaloids from the Gorgonian coral-derived fungus *Scopulariopsis* sp. *Mar. Biotechnol. (N. Y.)* **2015**, *17*, 408-415, doi:10.1007/s10126-015-9628-x.
175. Raina, J.B.; Tapiolas, D.; Motti, C.A.; Foret, S.; Seemann, T.; Tebben, J.; Willis, B.L.; Bourne, D.G. Isolation of an antimicrobial compound produced by bacteria associated with reef-building corals. *PeerJ* **2016**, *4*, e2275, doi:10.7717/peerj.2275.
176. Brana, A.F.; Sarmiento-Vizcaino, A.; Osset, M.; Perez-Victoria, I.; Martin, J.; de Pedro, N.; de la Cruz, M.; Diaz, C.; Vicente, F.; Reyes, F., et al. Lobophorin K, a new natural product with cytotoxic activity produced by *Streptomyces* sp. M-207 associated with the deep-sea coral *Lophelia pertusa*. *Mar. Drugs* **2017**, *15*, doi:10.3390/md15050144.
177. Shnit-Orland, M.; Sivan, A.; Kushmaro, A. Antibacterial activity of *Pseudoalteromonas* in the coral holobiont. *Microb. Ecol.* **2012**, *64*, 851-859, doi:10.1007/s00248-012-0086-y.
178. Zhang, X.; Sun, Y.; Bao, J.; He, F.; Xu, X.; Qi, S. Phylogenetic survey and antimicrobial activity of culturable microorganisms associated with the South China Sea black coral *Antipathes dichotoma*. *FEMS Microbiol. Lett.* **2012**, *336*, 122-130, doi:10.1111/j.1574-6968.2012.02662.x.
179. Jia, Y.-L.; Guan, F.-F.; Ma, J.; Wang, C.-Y.; Shao, C.-L. Pestalotiolide A, a new antiviral phthalide derivative from a soft coral-derived fungus *Pestalotiopsissp. Nat. Prod. Sci.* **2015**, *21*, 227, doi:10.20307/nps.2015.21.4.227.
180. Hawas, U.W.; El-Desouky, S.; Abou El-Kassem, L.; Elkhateeb, W. Alternariol derivatives from *Alternaria alternata*, an endophytic fungus residing in red sea soft coral, inhibit HCV NS3/4A protease. *Appl. Biochem. Microbiol.* **2015**, *51*, 579-584, doi:10.1134/s0003683815050099.
181. Zhu, M.; Gao, H.; Wu, C.; Zhu, T.; Che, Q.; Gu, Q.; Guo, P.; Li, D. Lipid-lowering polyketides from a soft coral-derived fungus *Cladosporium* sp. TZP29. *Bioorg. Med. Chem. Lett.* **2015**, *25*, 3606-3609, doi:10.1016/j.bmcl.2015.06.072.
182. Liu, Z.; Xia, G.; Chen, S.; Liu, Y.; Li, H.; She, Z. Eurothiocin A and B, sulfur-containing benzofurans from a soft coral-derived fungus *Eurotium rubrum* SH-823. *Mar. Drugs* **2014**, *12*, 3669-3680, doi:10.3390/md12063669.
